# Rapid and Green Anion-Assisted Mechanochemical Peptide Cyclization

**DOI:** 10.1021/acssuschemeng.4c03309

**Published:** 2025-01-03

**Authors:** Mirko Duvnjak, Nikolina Vidović, Krunoslav Užarević, Gordan Horvat, Vladislav Tomišić, Giovanna Speranza, Nikola Cindro

**Affiliations:** † Department of Chemistry, Faculty of Science, University of Zagreb, Horvatovac 102a, 10000 Zagreb, Croatia; ‡ Faculty of Biotechnology and Drug Development, University of Rijeka, R. Matejčić 2, 51000 Rijeka, Croatia; § Ruđer Bošković Institute, Bijenička c. 54, 10000 Zagreb, Croatia; ∥ Department of Chemistry, University of Milan, Via C. Golgi 19, 20133 Milan, Italy

**Keywords:** ball-milling, cyclization, green chemistry, peptides and proteins, solvent-free

## Abstract

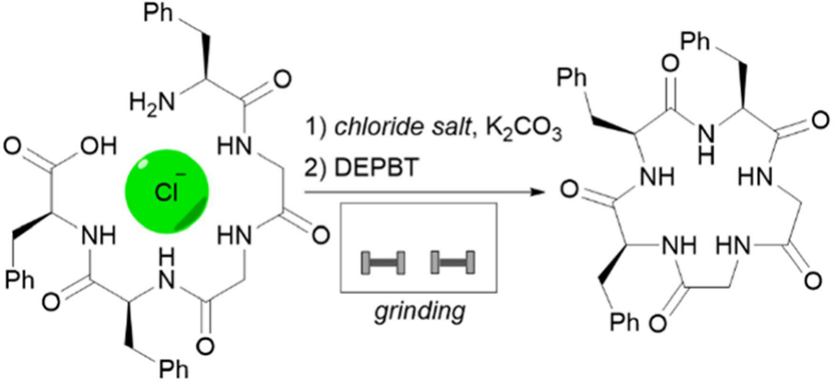

A novel mechanochemical approach is described for chloride-templated head-to-tail macrocyclization of a pentapeptide and a hexapeptide. This straightforward method allows the solvent-free preparation of cyclopeptides with yields comparable to solution-based approaches without the need for high dilution of the reaction mixture and with significantly reduced reaction times and organic waste amount.

## Abstract 2

A straightforward mechanochemical head-to-tail peptide macrocyclization method was developed, offering a solvent-free alternative to solution-based methods.

## Introduction

Since the discovery of Gramicidin S, there have been a lot of research efforts aimed at finding high-yielding experimental conditions for the preparation of cyclic peptides. In the last 2 decades, almost 20 new cyclic peptides were approved for clinical use.^1^ Synthesis of cyclopeptides represents a significant synthetic challenge, especially in the head-to-tail cyclization of small peptides containing up to seven all-L-amino acids. Besides ring size,^2^ the selection of coupling reagents plays an important role.^3^ Additionally, the macrocyclization step requires high dilution to minimize unwanted intermolecular processes such as oligo- and polymerization.^4^ The success of cyclization depends on the ability of the linear precursor to conformationally preorganize its reactive ends, forming in this way entropically unfavorable structures.^5^ In such structures, reactive ends are in spatial proximity, which favors macrocyclization over intermolecular processes. Over the years, many strategies relying on conformational preorganization have been developed. They can be classified under two categories: (1) internal, which requires covalent modification of the peptide chain, and (2) external, based on molecular scaffolds that are neither covalently bound to the peptides nor consumed during the cyclization reaction.^6^ Internal conformational elements include the introduction of turn-inducing elements by the incorporation of Pro in the middle of the peptide chain, D-amino acids, β-amino acids, or *N*-methyl amino acids in the sequence.^7^ Other methods of ring formation require the use of different chemical reactions, such as the creation of lactam bridges or disulfide bridges, which are most commonly used for sequences with two cysteine amino acids,^8,9^ insertion of heterocycle rings such as triazole,^10^ imidazole,^11^ oxazole,^11^ or thiazole rings,^12^ and metathesis.^13^ Also, new methods have been used in the past decade to cyclize linear peptides using enzymes^9,14^ and microwaves.^15^ Most of these methods are sequence-dependent and cannot be applied generally. In addition, the reaction yields are mostly very low. External conformational elements include the design of cavities large enough for only one linear peptide to enter and cyclize at a time^16,17^ or ion-assisted macrocyclization.^18,19^ The inspiration for the last strategy was found in nature; specifically, it relies on the well-known ability of cyclopeptides to form stable complexes with metal ions in vivo.^20,21^ In one of our previous studies, we demonstrated that not only cations but also anions can act as directing agents for promoting the cyclization of linear peptides.^22^ In particular, we found that for cyclization of tetra-, penta-, and hexapeptides, the best results were achieved when the salt containing a weakly coordinating cation [such as tetrabutylammonium (TBA) or tetraethylammonium (TEA)] and a chloride anion was used to assist the macrocyclization reaction. In this way, we prepared several cyclopeptides in moderate to relatively high yields. The described method is simpler and cheaper than most previously mentioned strategies, and more importantly, it does not depend on the peptide’s secondary structure. However, the method has two drawbacks: it requires high dilution (1 mg of linear precursor in 1 mL of DMF), and the reaction takes 3–5 days to yield the desired product.^22^ Besides, linear peptides bearing free NH_2_ and COOH groups at the termini usually suffer from low solubility in organic solvents. Mechanochemistry has emerged as a powerful green synthetic alternative to the conventional methods that promote efficient and rapid chemical reactions between solids,^23,24^ overcoming at the same time solubility and solvation issues from which solution-based peptide synthesis suffers. Combining thermal and mechanical energies by thermo-milling,^25^ the unprotected glycine or alanine with mineral additives afforded linear oligopeptides with up to 11 aa units.^26^ Cutting-edge work in mechanochemical peptide-bond formation has been done by Lamaty et al. using urethane-protected α-amino acid *N*-carboxyanhydride derivatives to afford various dipeptides when coupled with α-amino acid esters.^27^ This strategy was then used for the solvent-free synthesis of the opioid neurotransmitter Leu-enkephalin.^28^ The scope of this method, however, is limited by the low availability of amino acid *N*-carboxyanhydrides. Other developed coupling methods for protected amino acids were either limited to small-scale preparation of dipeptides or required harmful additives like 4-dimethylaminopyridine, PPh_3_, and cyanuric chloride.^29,30^ The other interesting strategy involves direct coupling of commercially available *N*-protected α-amino acids with α-amino acid esters using 1-ethyl-3-(3-(dimethylamino)propyl)carbodiimide as the coupling reagent, oxyma as the epimerization suppressant, and NaH_2_PO_4_ as a base. The deprotection steps were also performed under solvent-free conditions using gaseous HCl. This method afforded a wide range of di-, tri-, and tetrapeptides in good to excellent yields and multigram scales.^31^ However, to the best of our knowledge, mechanochemical peptide macrocyclization has not been described in the literature. As a part of research in peptide cyclization using anions as templating reagents, we envisioned a mechanochemical approach to cyclize oligopeptides to avoid the need for high dilution of reactants in formamide solvents, long reaction time, and complicated workup, from which the solution-based approach suffers.

## Experimental Section

For detailed experimental procedures and characterization data, please see the Supporting Information.

### General Procedure for Macrocyclization in Solution

To a solution of deprotected peptide **1**–**3** (1 mmol) and (3-(diethoxyphosphoryloxy)-1,2,3-benzotriazin-4(3H)-one) (DEPBT, 1.1 mmol) in DMF (500 mL) were added tetraethylammonium chloride (TEACl, 15 mmol) and TEA (2 mmol), and the reaction mixture was stirred at room temperature for 3–5 days. The solvent was removed under reduced pressure, EtOAc (200 mL) and water (200 mL) were added, the mixture was transferred to a separatory funnel, and the layers were separated. The organic layer was dried over Na_2_SO_4_, filtered, and concentrated. Further purification was done by column chromatography (5–10% MeOH in DCM).

### General Procedure for Mechanochemical Macrocyclization

Deprotected peptide **1**–**3** (0.02 mmol), chloride salt (15 equiv), and K_2_CO_3_ (2 equiv) (and in case of LAG 10 μL of liquid) were added to a steel jar (5 mL internal volume), two stainless steel balls (5 mm in diameter) were added, and the contents were ground in a vibratory ball mill for 45 min at a frequency of 30 Hz. DEPBT (1.1 equiv) was added, and the contents were ground again for another 120 min at 30 Hz. Reaction yield was calculated from HPLC (calibration curves constructed from purified cyclopeptides) as described in the SI. On scale up experiment, product was isolated using column chromatography as described in the SI.

## Results and Discussion

**Scheme 1:**
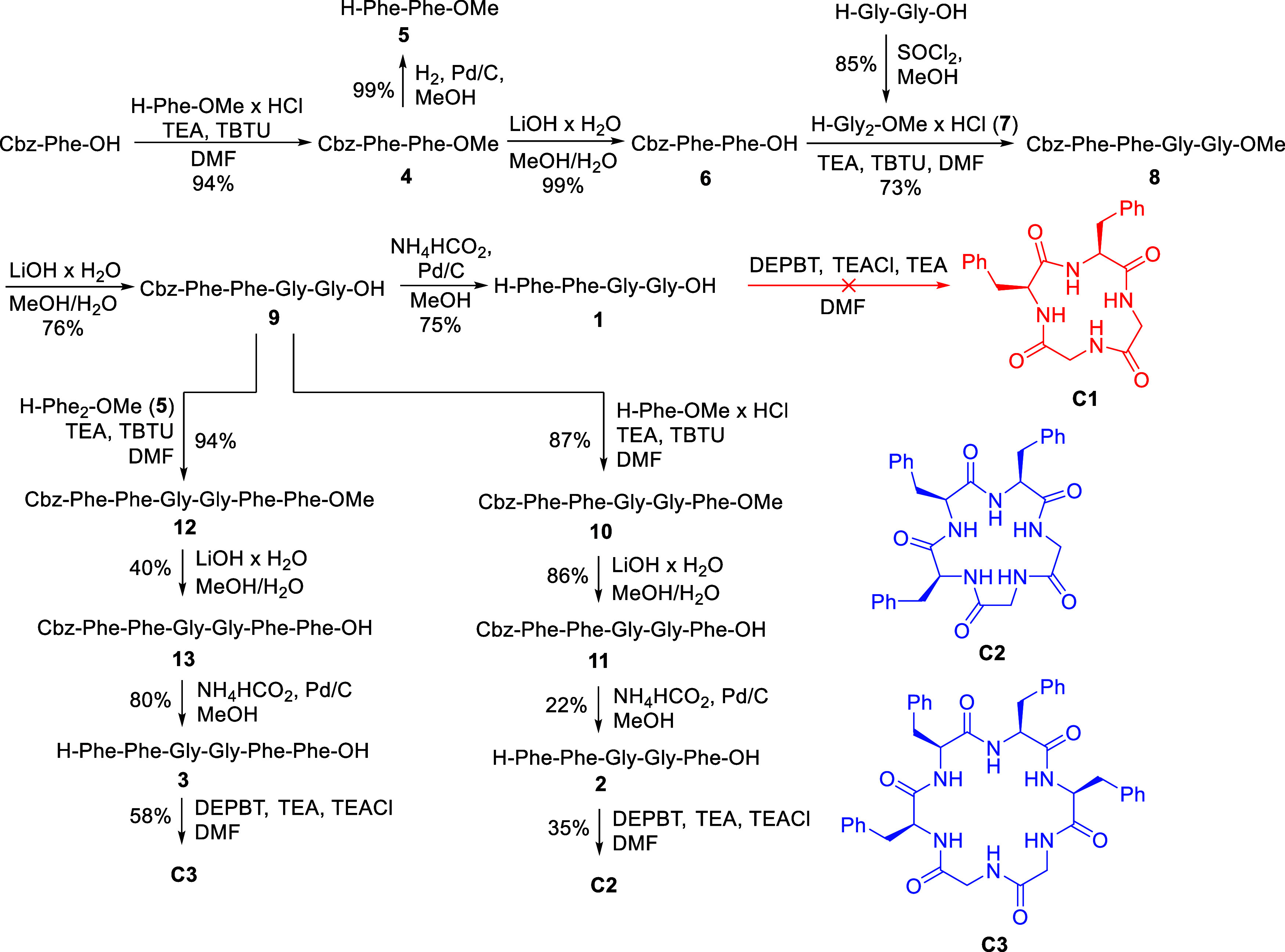
Synthesis of Linear and Cyclic Peptides using a Solution-based Approach

To investigate mechanochemical peptide macrocyclization, we prepared three linear peptides: tetrapeptide **1** (NH_2_–Phe-Phe-Gly-Gly–COOH), pentapeptide **2** (NH_2_–Phe-Phe-Gly-Gly-Phe–COOH), and hexapeptide **3** (NH_2_–Phe-Phe-Gly-Gly-Phe-Phe–COOH) on gram-scale. The synthesis was performed using a solution-based approach starting from protected Phe and deprotected Gly-Gly dipeptide (Scheme 1). First, CbZ-Phe-COOH and NH_2_–Phe–COOMe were condensed using 2-(1H-benzotriazole-1-yl)-1,1,3,3-tetramethylaminium tetrafluoroborate. The prepared fully protected dipeptide **4** was hydrolyzed using LiOH to obtain dipeptide **6**, which was then condensed with dipeptide **7** previously prepared by Fischer esterification of NH_2_-Gly-Gly-COOH. The obtained tetrapeptide **8** was subjected to ester hydrolysis, yielding compound **9**, which was then used to prepare deprotected tetrapeptide **1** by transfer hydrogenation removal of the CbZ protecting group. Compound **9** was also used to prepare deprotected pentapeptide **2** and hexapeptide **3** through the coupling with NH_2_–Phe–COOMe and NH_2_–Phe-Phe–COOMe (**5**), respectively, followed by protecting groups removal in the same fashion as for **1**.

With linear peptides **1–3** in hand, we started exploring the cyclization reaction. As the first approach, we used previously described solution-based synthesis (TEACl as a source of chloride anions and DEPBT as a coupling agent).^22^ Here, we obtained the known cyclic pentapeptide **C2**(32) and new cyclic hexapeptide **C3** in 35 and 58% yields, respectively. Compound **1** failed to cyclize, which was not surprising, as it is known that tetrapeptides are difficult to cyclize due to the ring size. Products **C2** and **C3** were isolated by using column chromatography. After confirming the structure and purity of the compounds, we used them as standards for future HPLC yield determination. It is important to underline that solution-based cyclizations require 1 mL of DMF per 1 mg of peptide, and the reaction is run for 3 to 5 days, followed by solvent removal.

We started the screening of mechanochemical conditions by milling pentapeptide **2** (1 equiv) with potassium carbonate (2 equiv) as a base and sodium chloride (15 equiv) as a source of chloride anions, which can form complexes with linear peptides, as was already proven.^33−36^ Milling was stopped after 45 min, and after DEPBT (1.1 equiv) was added, milling was continued for another 120 min. The conversion was then determined by HPLC (Table 1). Detailed experimental procedures and HPLC analysis are described in the SI. The initial optimization was performed on pentapeptide **2**, as shown in Table 1. To prove that the addition of (chloride) salt plays a key role also in the mechanochemical macrocyclization of peptides, as is the case with the solution-based approach, we performed a cyclization reaction without the addition of chloride salt, and we did not observe a formation of cyclic peptide under these conditions. Next, we tried adding alkaline and alkaline earth chlorides as a cheap and readily available source of chloride ions. Yields in these experiments did not exceed 6%. After several runs with similar outcomes, the focus was turned to quaternary ammonium salts with lower crystalline lattice enthalpy than metal chlorides. The yields obtained in this way were comparable to those observed in the solution approach for pentapeptide **2** (39% mechanochemical vs 35% solution-based approach) and lower for hexapeptide **3** (25% mechanochemical vs 58% solution-based approach). The effect of the cation was further investigated, and the results are presented later.

**Table 1:**
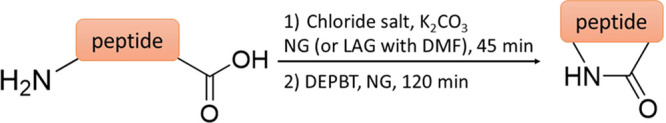
Optimization of the Mechanochemical Cyclation of Linear Peptides^a^

peptide^b^	Salt^c^	eq K_2_CO_3_	LAG/NG^d^	conversion (HPLC)^e^
**2**	No salt added	2	NG	0%
**2**	NaCl (15 equiv)	2	LAG (DMF)	4%
**2**	KCl (15 equiv)	2	NG	6%
**2**	KCl (15 equiv)	2	LAG (DMF)	3%
**2**	CaCl_2_ (15 equiv)	2	NG	5%
**2**	CaCl_2_ (15 equiv)	4	NG	3%
**2**	TEACl (15 equiv)	2	LAG (DMF)	18%
**2**	TEACl (15 equiv)	2	NG	24%
**2**	TEACl (5 equiv)	2	NG	28%
**2**	TEACl (10 equiv)	2	NG	30%
**2**	TEACl (20 equiv)	2	NG	27%
**2**	TEACl (25 equiv)	2	NG	27%
**2**	TEACl (30 equiv)	2	NG	30%
**2**	BTEACl (15 equiv)	2	NG	39%
**2**	BTEACl (15 equiv)	4	NG	31%
**2**	BTEACl (15 equiv)	6	NG	33%
**2**	BTEACl (15 equiv)	2	LAG (EtOAc)	18%
**2**	BTEACl (15 equiv)	2	LAG (H_2_O)	2%
**2**	BTEACl (15 equiv)	2	LAG (DMSO)	11%
**2**	BTEACl (15 equiv)	2	LAG (dioxane)	14%
**3**	TEACl (15 equiv)	2	NG	20%
**3**	TEACl (15 equiv)	4	NG	23%
**3**	BTEACl (15 equiv)	2	NG	22%
**3**	BTEACl (15 equiv)	4	NG	25%

a The conventional solution-based approach requires 1 mL of DMF per mg of peptide and multiday reactions.

b In all reactions, 0.02 mmol of linear peptides was used, corresponding to 11.5 mg of pentapeptide 2 and 14.5 mg of hexapeptide 3, respectively.

c All reactions were performed in a steel jar (5 mL internal volume) with two 0.5 g stainless steel balls at a 30 Hz operating frequency.

d LAG (liquid-assisted grinding) refers to adding 10 μL of solvent to the reaction mixture before milling. NG stands for neat grinding.

e HPLC conversions were in agreement with isolated yields see SI.

TEACl was used first. It was observed that amounts higher than 10 equiv do not profoundly impact the yield, so we fixed the amount to 15 equiv, which was sufficient for good rheology of the mixture during milling. Interestingly, the addition of a small volume of DMF as an additive in liquid-assisted grinding (LAG) experiments, as well as the increase in added base (K_2_CO_3_), led to decreased yields. Benzyltriethylammonium chloride (BTEACl) was next tried as a source of the chloride anions, and nearly 40% yield was obtained which was the highest in the scope of this optimization. BTEACl, together with TEACl and TBACl, gave the best results in terms of reaction yield and also in the solution-based approach.^22^ After the described optimization, the mechanochemical cyclization reaction of hexapeptide **3** using both TEACl and BTEACl was carried out, and yields of 23 and 25% were obtained, respectively. The observed yields were lower than those corresponding to the solution-based approach in which the same starting material was used together with TEACl. Given that the mechanochemical reaction is run without a solvent and requires less than 2 h, the E-factor for the synthetic step is reduced from 2968 in a solution-based approach to only 21 in a mechanochemical approach (detailed description of E-factor calculation is reported in SI). Both procedures require column separation. However, the solution procedure has an additional step because the large volume of DMF must be carefully evaporated before the crude mixture is subjected to the column step. We also tried to prepare the cyclic tetrapeptide **C1** using mechanochemical synthesis, but, similar to the solution approach, the reaction did not yield the product.

**Figure 1:**
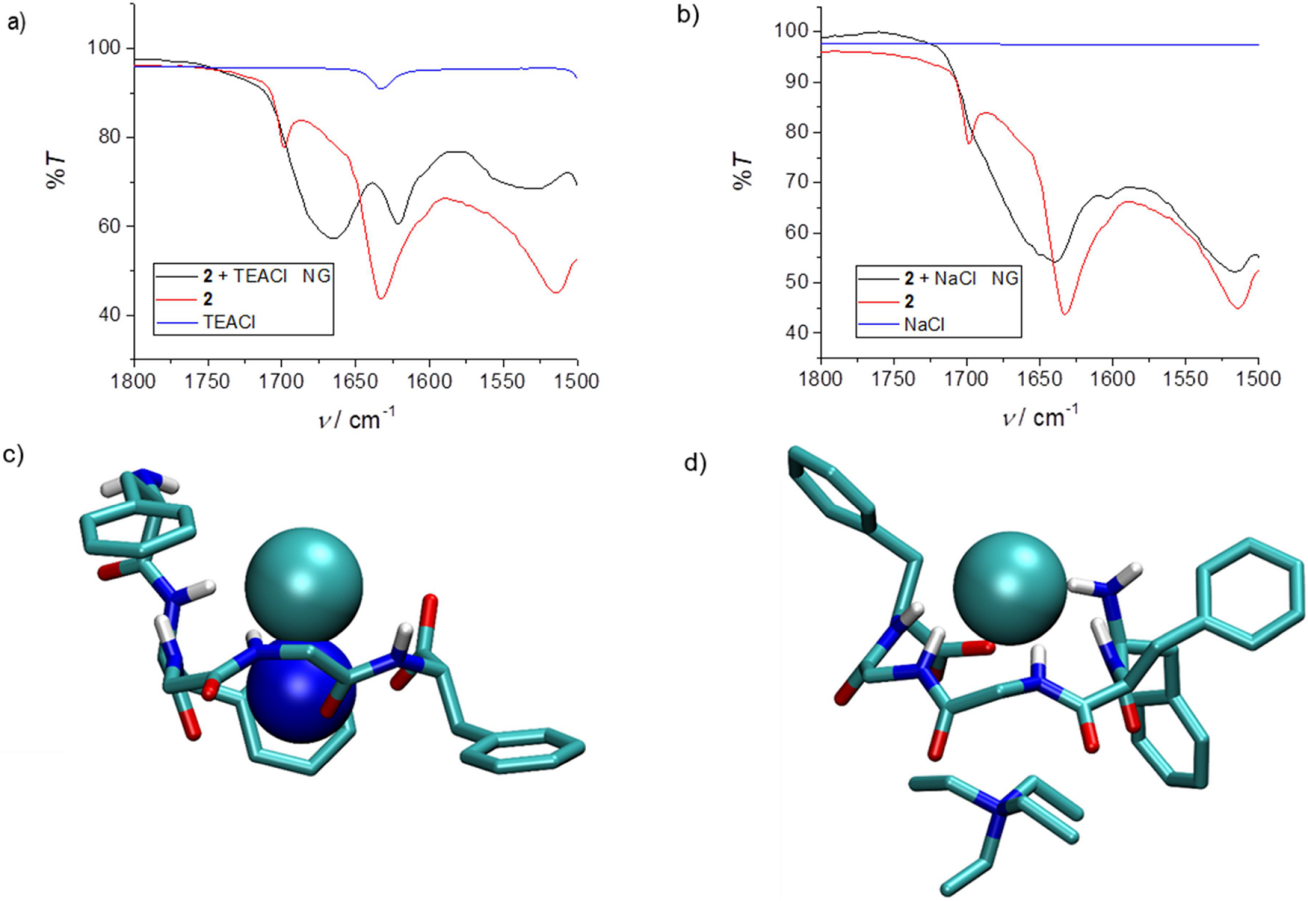
(a) IR spectra of linear peptide **2** (red), TEACl (blue), and a mixture of peptide and salt in a 1:1 molar ratio after milling for 30 min (black). (b) IR spectra of linear peptide **2** (red), NaCl (blue), and a mixture of peptide and salt in a 1:1 molar ratio after milling for 30 min (black). (c) Representative structure of the complex of **2** with Na (blue sphere)–Cl (green sphere) ion pair. (d) Representative structure of **2** complex with a TEACl ion pair obtained by MD simulations.

To further understand the role of quaternary ammonium salts, we compared the IR spectra of pentapeptide **2** and a mixture of **2** and TEACl after milling (DEPBT was not added). In this experiment, changes in the carbonyl region were observed, which indicates the complexation of chloride anion by a linear precursor, as shown in Figure 1a. After the same procedure was repeated using sodium chloride instead of TEACl, the shifts in the carbonyl region of IR spectra were significantly lower, indicating a different type of interaction (Figure 1b). The origin of this effect could lie in the limited availability of free chloride anions originating from sodium chloride due to the strong lattice interaction. The lattice energy of sodium chloride crystal^37^ is about 360 kJ mol^–1^ higher than for the crystalline tetraethylammonium chloride,^38^ suggesting that the exclusion of the chloride anion is more feasible when the latter salt is used. The reported values correspond to the bulk of the crystal, whereby, in the case of sodium chloride, the lattice enthalpy decreases strongly with the size reduction of the crystalline particles.^37^ This is most probably the case with tetraethylammonium chloride as well. The milling process results in a microcrystalline powder when either salt is used. In the case of very small crystals, lattice enthalpies approach ion-pair interaction values.^37^ In the milling process, the crystal lattice chloride anion is complexed by the peptide receptor, and the counterion is most probably close to the chloride complex, thus forming an ion pair. The specific interactions of sodium and tetraethylammonium cations with the chloride-peptide complex could be responsible for the outcome of the cyclization step. We conducted molecular dynamics simulations of the complexes under vacuum at room temperature to elucidate this effect. The results of these simulations indicate that the sodium cation forms an intimate ion pair with the complexed chloride anion (Figure 1c). The overall conformation is quasi-cyclic in which the ion pair is perpendicular to the ring, and the amine protons do not participate in anion coordination. On the other hand, the tetraethylammonium cation does not interact specifically with the complexed chloride anion, and the complex structure is more suitable for the cyclization reaction (Figure 1d).

The reaction was attempted in a planetary ball mill to test the possibility of mechanochemical peptide macrocyclization in different mechanochemical reactors and on a larger scale. Around 350 mg of pentapeptide, ca. 30x the quantity commonly used for the vibrational mill, was mixed with BTEACl and K_2_CO_3_, followed by the addition of DEPBT. After 30 min of milling at 650 rpm, HPLC analysis indicated 20% conversion. Column chromatography was performed to establish a further correlation with HPLC conversions. The mixture was purified on a silica gel column using a dry loading technique to yield 61 mg (18%) of pure product. The drawback of using a planetary ball mill was that the reaction mixture tended to heat up, and after less than 30 min, the temperature reached around 70 °C. The reaction was also run stepwise by milling and cooling the jar in a freezer, but this approach resulted in even smaller yields when compared to those obtained without cooling. Developing a planetary ball mill with temperature regulation for reactions with thermally sensitive materials would be beneficial.

## Conclusions

For the first time, macrocyclization of linear peptides was performed using a mechanochemical approach. We demonstrated here that templated mechanochemical peptide macrocyclization can be run completely without a solvent, thus avoiding the need for high dilution in DMF and reducing reaction times from days to minutes. The linear precursors NH_2_–Phe-Phe-Gly-Gly-Phe–COOH and NH_2_–Phe-Phe-Gly-Gly-Phe-Phe–COOH were prepared and then successfully cyclized in a head-to-tail fashion by milling linear peptides with potassium carbonate as a base and DEPBT as the coupling agent in the presence of different salts. Among these, the best results were achieved when quaternary ammonium salts, particularly benzyltriethylammonium chloride, were used as a source of chloride anions for templating cyclization reactions. The observed yields were lower than those corresponding to the solution-based approach. However, the purification of cyclopeptides from the mill was significantly more straightforward and greener since the need for removal of DMF was avoided entirely. Both procedures require a column separation step, as usual in cyclopeptide chemistry. On eco scales, the E-factor for the synthetic step was reduced from 2968 for the solution-based approach to a mere 21 for the mechanochemical approach. Finally, the possibility of running reactions on a larger scale was examined as well. Future research will focus on optimizing large-scale procedures and developing environmentally benign mechanochemical cyclization of industrially interesting linear peptide precursors.

## Supporting Information Available

Detailed experimental procedures, ^1^H and ^13^C NMR spectra, HRMS spectra, and HPLC chromatograms (PDF)
