# Supplementary material for: Rapid and Green Anion-Assisted Mechanochemical Peptide Cyclization
Source: ACS Sustain Chem Eng. 2025 Jan 3;13(1):30–5. doi: 10.1021/acssuschemeng.4c03309 (PMC11734432; doi:10.1021/acssuschemeng.4c03309)
Supplement: Supplementary file 1 — sc4c03309_si_001.pdf [file sc4c03309_si_001.pdf]

Supplementary Information

Rapid and green anion-assisted mechanochemical peptide cyclization

Mirko Duvnjak[a‡], Nikolina Vidović[b‡], Krunoslav Užarević[c], Gordan Horvat[a], Vladislav Tomišić[a], Giovanna Speranza[d], Nikola Cindro[a]*

Email: ncindro.chem@pmf.hr

a) Department of Chemistry, Faculty of Science, University of Zagreb, Horvatovac 102a, 10000 Zagreb, Croatia
b) Faculty of Biotechnology and Drug Development, University of Rijeka, R. Matejčić 2, 51000 Rijeka, Croatia
c) Ruđer Bošković Institute, Bijenička c. 54, Zagreb 10000, Croatia
d) Department of Chemistry, University of Milan, Via C. Golgi 19, 20133 Milan, Italy

‡These authors contributed equally as co-first authors.

number of pages: 57
number of tables: 3
number of figures: 74

Table of contents

1. Solution based synthesis and cyclizations of linear peptides **1**–**3** 3

1.1. General methods and materials 4

1.2. Synthesis of tetrapeptide H-Phe-Phe-Gly-Gly-OH (**1**) 4

1.3. Synthesis of pentapeptide H-Phe-Phe-Gly-Gly-Phe-OH (**2**) 7

1.4. Synthesis of hexapeptide H-Phe-Phe-Gly-Gly-Phe-Phe-OH (**3**) 9

1.5. Chloride-assisted macrocyclizations of peptides **2** and **3** – solution-based approach 12

2. Mechanochemical macrocyclizations of oligopeptides 14

2.1. General procedure for the mechanochemical macrocyclizations of oligopeptides 14

2.2. Synthesis of cyclo-L-phenylalanyl-L-phenylalanyl-L-phenylalanyl-glycylglycine (**C2**) 18

2.3. Synthesis of cyclo-L-phenylalanyl-L-phenylalanyl-L-phenylalanyl-L-phenylalanyl - glycylglycine (**C3**) 19

2.4. Calculation of E-factor for macrocyclization step 19

2.5. Procedure for the scaled-up mechanochemical cyclization of peptide **2** 20

3. Procedure for the molecular dynamics simulations of **C2** complexes 20

4. References 22

5. NMR and HRMS spectra 23

6. HPLC chromatograms of samples from grinding experiments 44

1. Solution based synthesis and cyclizations of linear peptides 1–3

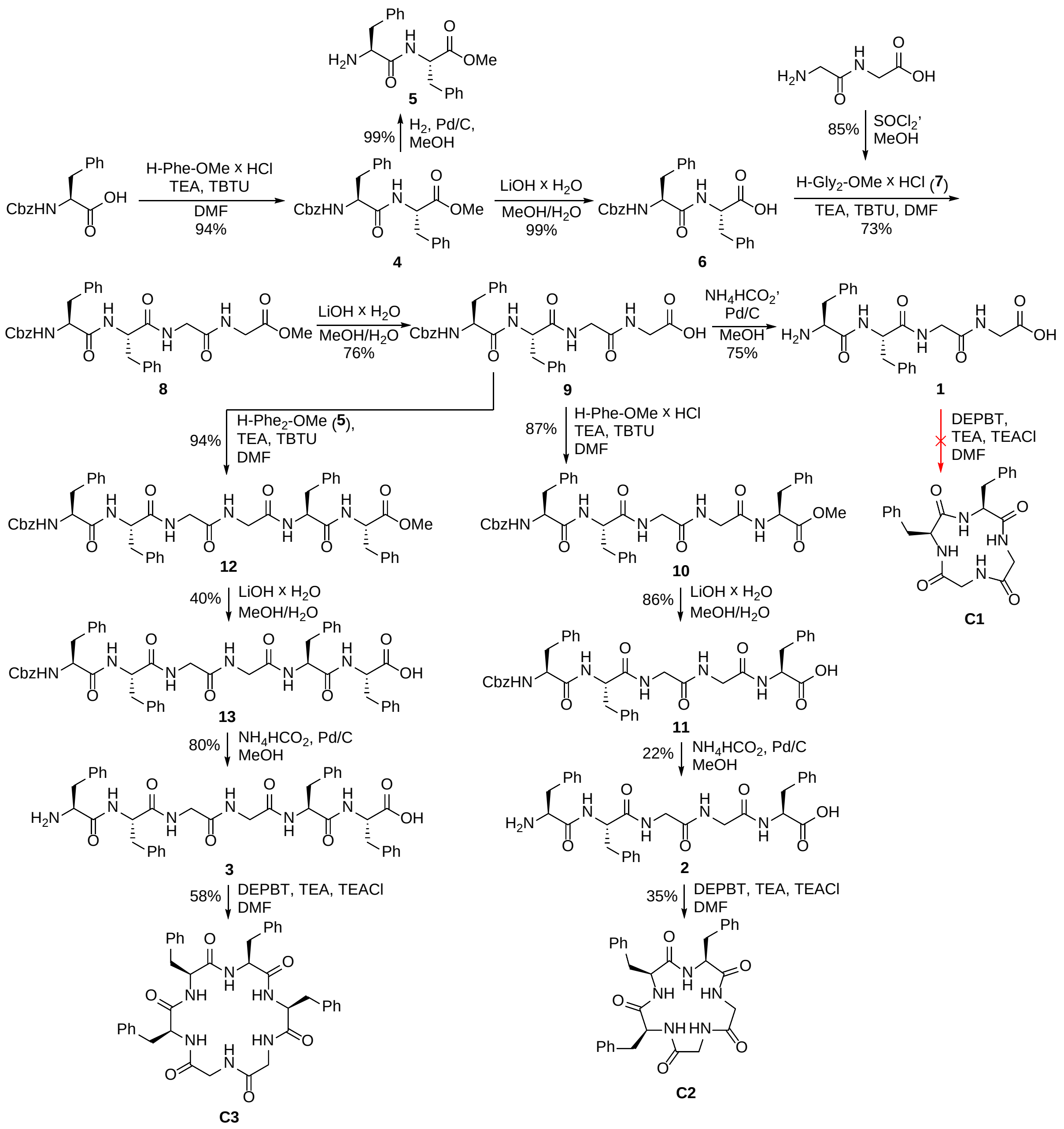

Figure S1. Liquid phase synthesis and chloride-assisted macrocyclizations of linear peptides **1 – 3**

1.1. General methods and materials

All solvents and reagents were purchased from Sigma-Aldrich, Carbolution or Merck and were used without further purification. Analytical Thin Layer Chromatography (TLC) was performed on silica gel 60 F254 precoated aluminum sheets with a fluorescent indicator (0.2 mm layer; Merck, Darmstadt, Germany) and the components were detected under a UV lamp (λ 254 nm). Silica gel chromatography was performed using Merck silica gel (60, particle size 0.040 – 0.063 mm). ^{1}H and ^{13}C NMR spectra were acquired by means of a Bruker Ascend 400 MHz spectrometer (Bruker, Carlsruhe, Germany). Solutions of compounds were prepared in $CDCl_3$ or DMSO-*d6* with tetramethylsilane (TMS) as the internal standard. Chemical shifts (δ) are given in ppm and are referenced to TMS in ^{1}H NMR spectra and to solvent signals in ^{13}C NMR spectra. The high-resolution mass spectra (HRMS) were recorded on a Thermo Fisher Scientific Q Exactive ESI Orbitrap mass spectrometer. Mechanochemical reactions were performed using IST 500 mixer mill (InSolido Technologies) or Retsch PM 200 planetary ball mill.

1.2. Synthesis of tetrapeptide H-Phe-Phe-Gly-Gly-OH (1)

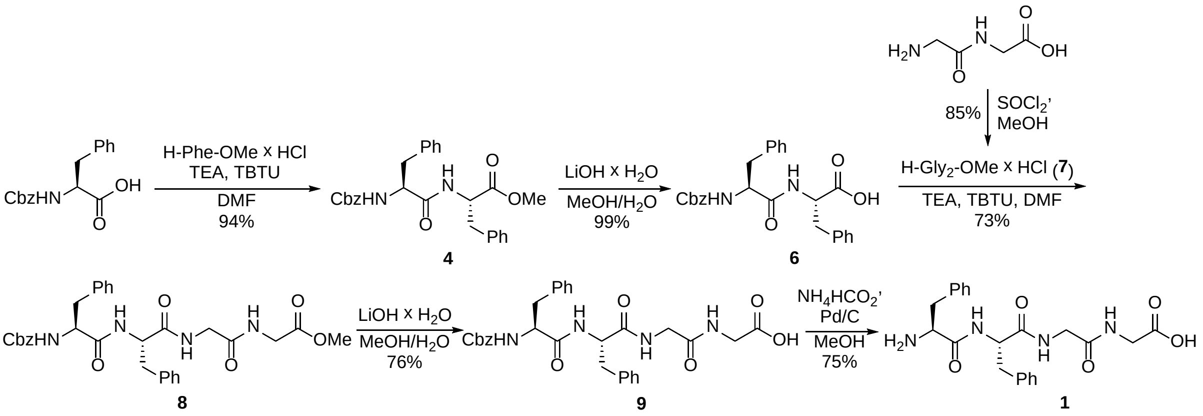

Synthesis of *N*-(carbobenzyloxy)-L-phenylalanyl-L-phenylalanine methyl ester (4)

N-(carbobenzyloxy)-L-phenylalanine (6.94 g, 23.2 mmol, 1 equiv) and L-phenylalanine methyl ester hydrochloride (5.00 g, 23.2 mmol, 1 equiv) were dissolved in DMF (150 mL). To the cooled mixture (0 °C) were added TEA (6.40 mL, 45.9 mmol, 2 equiv), then TBTU (8.11 g, 25.3 mmol, 1.1 equiv) in small portions and the reaction mixture was stirred at room temperature overnight. The mixture was cooled to 0 °C and water (300 mL) was added dropwise to precipitate the product. The precipitate was filtered off under reduced pressure, washed with DMF:H_2O (1:2),

water and dried in air. 10.08 g (94 %) of **4** was obtained as a white powder and was used in the next step without further purification. ^{1}H and ^{13}C NMR spectra are in accordance with previously recorded spectra.[1]

Synthesis of *N*-(carbobenzyloxy)-L-phenylalanyl-L-phenylalanine (6)

Compound **4** (6.37 g, 13.8 mmol, 1 equiv) was suspended in methanol (175 mL) and to the stirred mixture LiOH · H_2O (0.64 g, 15.2 mmol, 1.1 equiv) and water (50 mL) were added. The reaction mixture was stirred at room temperature for 48 h. Methanol was evaporated under reduced pressure, the mixture was cooled to 0 °C and HCl (18.0 mL, c = 1 mol dm^{-3}) was added dropwise. The resulting mixture was strongly stirred for 10 minutes, transferred to a separatory funnel and extracted with EtOAc (100 mL + 2×50 mL). The organic extracts were combined, washed with brine (2×50 mL), dried over Na_2SO_4, filtered and concentrated *in vacuo*. 6.13 g (99 %) of **6** was obtained as a white powder and was used in the next step without further purification. ^{1}H and ^{13}C NMR spectra are in accordance with previously recorded spectra.[2]

Synthesis of glycylglycine methyl ester hydrochloride (7)

Glycylglycine (5.00 g, 37.9 mmol, 1 equiv) was suspended in dry methanol (150 mL), the mixture was cooled to 0 °C and thionyl chloride (5.00 mL, 68.9 mmol, 1.8 equiv) was slowly added. The reaction mixture was stirred overnight at room temperature and concentrated *in vacuo*. The crude solid was washed with benzene or toluene and again concentrated *in vacuo*. The crude product was recrystallized from isopropanol. 5.88 g (85 %) of pure **7** was obtained as a white powder.

^{1}H NMR (400 MHz, DMSO-d_6) δ/ppm: 9,01 (t, J = 5,8 Hz, 1H), 8,30 (s, 3H), 3,95 (d, J = 5,8 Hz, 2H), 3,65 (s, 3H), 3,60 (s, 2H).

^{13}C NMR (100 MHz, DMSO-d_6) δ/ppm: 169,9, 166,6, 51,9, 40,5, 39,9.

HRMS (ESI) *m/z* $[M + H]^+$ – calculated ($C_5H_{11}N_2O_3^+$) – 147,0725, found 147,0767.

Synthesis of *N*-(carbobenzyloxy)-L-phenylalanyl-L-phenylalanylglycylglycine methyl ester (8)

Compounds **6** (2.42 g, 13.2 mmol, 1 equiv) and **7** (5.91 g, 13.2 mmol, 1 equiv) were dissolved in DMF (150 mL). To the cooled mixture (0 °C) were added TEA (3.70 mL, 26.5 mmol, 2 equiv), then TBTU (4.67 g, 14.6 mmol, 1.1 equiv) in small portions and the reaction mixture

was stirred at room temperature overnight. The mixture was cooled to 0 °C and water (150 mL) was added dropwise. EtOAc (150 mL) was added, the bilayer system was stirred strongly for 10 minutes and the precipitate was filtered off under reduced pressure. 2.42 g of pure **8** was obtained as a white powder. The bilayer system was transferred to a separatory funnel and the layers were separated. The organic layer was washed with brine (50 mL), dried over Na_2SO_4, filtered and concentrated *in vacuo*. The crude solid was recrystallized from isopropanol (50 mL) and diethyl ether (100 mL) which afforded another 3.14 g of nearly pure **8**. A total of 5.56 g (73 %) of **8** was obtained as a white powder and was used in the next step without further purification.

^{1}H NMR (400 MHz, DMSO-d_6) δ/ppm: 8,32 (t, J = 5,7 Hz, 1H), 8,23–8,18 (m, 2H), 7,47 (d, J = 8,8 Hz, 1H), 7,35–7,03 (m, 15H), 4,96–4,78 (m, 2H), 4,59–4,54 (m, 1H), 4,28–4,22 (m, 1H), 3,88 (d, J = 5,9 Hz, 2H), 3,83–3,70 (m, 2H), 3,62 (s, 3H), 3,07 (dd, J_1 = 13,9 Hz, J_2 = 4,8 Hz, 1H), 2,94–2,83 (m, 2H), 2,66 (dd, J_1 = 13,8 Hz, J_2 = 10,8 Hz, 1H).

^{13}C NMR (100 MHz, DMSO-d_6) δ/ppm: 171,6, 171,2, 170,2, 169,2, 155,7, 138,0, 137,6, 137,0, 129,3, 129,2, 128,3, 128,1, 128,0, 127,7, 127,4, 126,3, 126,2, 65,2, 56,0, 54,0, 51,7, 41,8, 40,5, 37,5, 37,4.

HRMS (ESI) *m/z* $[M + H]^+$ – calculated ($C_{31}H_{35}N_4O_7^+$) – 575,2461, found 575,2512.

Synthesis of *N*-(carbobenzyloxy)-L-phenylalanyl-L-phenylalanylglycylglycine (9)

Compound **8** (6.68 g, 11.6 mmol, 1 equiv) was suspended in methanol (200 mL) and added to the stirred solution LiOH · H_2O (0.54 g, 12.8 mmol, 1.1 equiv) and water (60 mL). The reaction mixture was stirred at room temperature for 48 h. Methanol was evaporated under reduced pressure, the mixture was cooled to 0 °C and HCl (15.0 mL, c = 1 mol dm^{-3}) was added dropwise. The precipitate was filtered off under reduced pressure, washed with water and dried in air. 4.97 g (76 %) of pure **9** was obtained as a white powder.

^{1}H NMR (400 MHz, DMSO-d_6) δ/ppm: 8,30 (t, J = 5,7 Hz, 1H), 8,18 (d, J = 7,9 Hz, 1H), 8,08 (t, J = 5,8 Hz, 1H), 7,47 (d, J = 8,8 Hz, 1H), 7,35–7,02 (m, 15H), 4,96–4,78 (m, 2H), 4,59–4,54 (m, 1H), 4,27–4,22 (m, 1H), 3,82–3,73 (m, 4H), 3,07 (dd, J_1 = 13,9 Hz, J_2 = 4,7 Hz, 1H), 2,95–2,83 (m, 2H), 2,66 (dd, J_1 = 13,7 Hz, J_2 = 10,7 Hz, 1H).

^{13}C NMR (100 MHz, DMSO-d_6) δ/ppm: 171,5, 171,2, 171,1, 169,0, 155,7, 138,0, 137,6, 137,0, 129,3, 129,2, 128,3, 128,1, 128,0, 127,7, 127,4, 126,3, 126,2, 65,2, 56,1, 54,0, 41,8, 40,7, 37,5, 37,4.

HRMS (ESI) *m*/*z* $[M + H]^+$ – calculated ($C_{30}H_{33}N_4O_7^+$) – 561,2305, found 561,2354.

Synthesis of L-phenylalanyl-L-phenylalanylglycylglycine (1)

Compound **9** (1.96 g, 3.5 mmol, 1 equiv) and ammonium formate (2.20 g, 35.0 mmol, 10 equiv) were dissolved in methanol, Pd/C was added and the mixture was refluxed for 3 hours. The reaction mixture was cooled to room temperature, filtered over a celite pad and concentrated *in vacuo*. To the crude solid water (50 mL) was added and the solid was filtered off under reduced pressure, washed with water and dried in air. 1.11 g (75 %) of pure **1** was obtained as a white powder.

^{1}H NMR (400 MHz, DMSO-d_6) δ/ppm: 8,42–8,38 (m, 2H), 7,88 (t, J = 5,4 Hz, 1H),7,31–7,02 (m, 10H), 4,55–4,56 (m, 1H), 3,81–3,62 (m, 4H), 3,54 (dd, J_1 = 13,6 Hz, J_2 = 4,8 Hz, 1H), 3,06 (dd, J_1 = 13,8 Hz, J_2 = 4,8 Hz, 1H), 2,92–2,83 (m, 2H), 2,58 (dd, J_1 = 13,6 Hz, J_2 = 8,5 Hz, 1H)

^{13}C NMR (100 MHz, DMSO-d_6) δ/ppm: 172,3, 171,3, 171,0, 168,6, 137,7, 137,5, 129,4, 129,3, 128,2, 128,1, 126,4, 126,3, 55,4, 53,7, 42,0, 41,5, 39,4, 37,5

HRMS (ESI) *m*/*z* $[M + H]^+$ – calculated ($C_{22}H_{27}N_4O_5^+$) – 427,1937, found 427,1983.

1.3. Synthesis of pentapeptide H-Phe-Phe-Gly-Gly-Phe-OH (2)

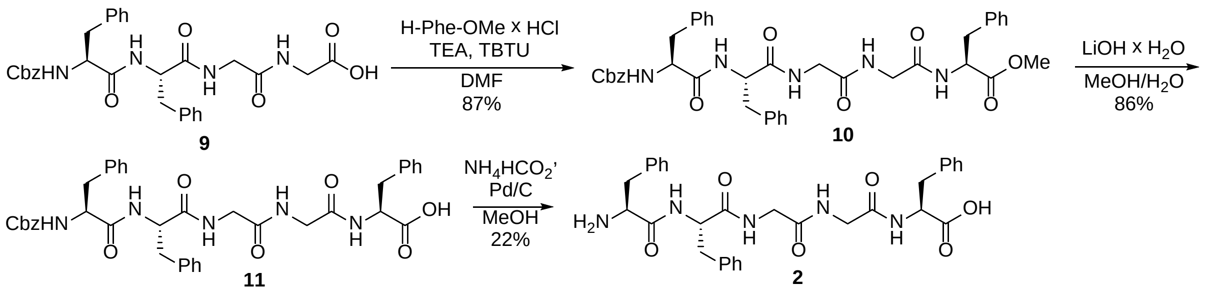

Synthesis of *N*-(carbobenzyloxy)-L-phenylalanyl-L-phenylalanylglycylglycyl-L-phenylalanine methyl ester (10)

Compound **9** (2.25 g, 4.0 mmol, 1 equiv) and L-phenylalanine methyl ester hydrochloride (0.87 g, 4.0 mmol, 1 equiv) were dissolved in DMF (75 mL). To the cooled mixture (0 °C) were added TEA (1.12 mL, 8.0 mmol, 2 equiv), then TBTU (1.42 g, 4.4 mmol, 1.1 equiv) and the reaction mixture was stirred at room temperature overnight. The mixture was cooled to 0 °C and water (75 mL) was added dropwise to precipitate the product. The precipitate was filtered

off under reduced pressure, washed with DMF:H_2O (1:1), water and dried in air. 2.53 g (87 %) of pure **10** was obtained as a white powder.

^{1}H NMR (400 MHz, DMSO-*d*$_6$) δ/ppm: 8,33 (d, J = 7,7 Hz, 1H), 8,27 (t, J = 5,5 Hz, 1H), 8,16 (d, J = 8,0 Hz, 1H), 8,00 (t, J = 5,7 Hz, 1H), 7,46 (d, J = 8,7 Hz, 1H), 7,34–7,01 (m, 20H), 4,96–4,78 (m, 2H), 4,60–4,54 (m, 1H), 4,50–4,45 (m, 1H), 4,27–4,21 (m, 1H), 3,79–3,65 (m, 4H), 3,57 (s, 3H), 3,09–2,63 (m, 6H)

^{13}C NMR (100 MHz, DMSO-*d*$_6$) δ/ppm: 171,8, 171,4, 171,3, 168,8, 168,7, 155,7, 138,1, 137,6, 137,03, 136,98, 129,3, 129,2, 129,1, 128,3, 128,04, 128,01, 127,7, 127,4, 126,6, 126,3, 126,2, 65,2, 56,1, 53,9, 53,6, 51,9, 42,0, 41,5, 37,6, 37,4, 36,8

HRMS (ESI) *m/z* $[M + H]^+$ – calculated ($C_{40}H_{44}N_5O_8^+$) – 722,3145, found 722,3195.

Synthesis of *N*-(carbobenzyloxy)-L-phenylalanyl-L-phenylalanylglycylglycyl-L-phenylalanine (11)

Compound **10** (3.40 g, 4.7 mmol, 1 equiv) was suspended in methanol (200 mL) and added to the stirred solution LiOH · H_2O (0.24 g, 5.7 mmol, 1.2 equiv) and water (50 mL). The reaction mixture was stirred at room temperature overnight. The mixture was then heated until everything dissolved and stirred at room temperature for another 24 h. Methanol was evaporated under reduced pressure, the mixture was cooled to 0 °C and HCl (10.0 mL, c = 1 mol dm^{-3}) was added dropwise. The precipitate was filtered off under reduced pressure, washed with water and dried in air. 2.86 g (86 %) of **11** was obtained as a white powder and was used in the next step without further purification.

^{1}H NMR (400 MHz, DMSO-*d*$_6$) δ/ppm: 8,28 (t, J = 5,6 Hz, 1H), 8,21 (d, J = 8,0 Hz, 1H), 8,09 (d, J = 7,9 Hz, 1H), 8,00 (t, J = 5,5 Hz, 1H), 7,48 (d, J = 8,7 Hz, 1H), 7,34–7,15 (m, 20H), 4,96–4,75 (m, 2H), 4,60–4,55 (m, 1H), 4,42–4,37 (m, 1H), 4,26–4,21 (m, 1H), 3,75–3,73 (m, 2H), 3,67 (dd, J_1 = 16,7 Hz, J_2 = 5,7 Hz, 2H), 3,09–3,03 (m, 2H), 2,94–2,82 (m, 3H), 2,67 (dd, J_1 = 13,4 Hz, J_2 = 11,1 Hz, 1H)

^{13}C NMR (100 MHz, DMSO-*d*$_6$) δ/ppm: 172,8, 171,4, 171,3, 168,8, 168,4, 155,7, 138,1, 137,73, 137,65, 137,0, 129,3, 129,2, 128,3, 128,1, 128,0, 127,7, 127,4, 126,33, 126,26, 126,2, 65,2, 56,1, 53,9, 53,8, 42,1, 41,7, 37,6, 37,4, 37,0

HRMS (ESI) *m/z* $[M + H]^+$ – calculated ($C_{39}H_{42}N_5O_8^+$) – 708,2989, found 708,3043.

Synthesis of L-phenylalanyl-L-phenylalanylglycylglycyl-L-phenylalanine (2)

Compound **2** (2.80 g, 4.0 mmol, 1 equiv) and ammonium formate (2.49 g, 39.6 mmol, 10 equiv) were dissolved in methanol (250 mL), Pd/C was added and the mixture was refluxed for 3 hours. The reaction mixture was cooled to room temperature, filtered over a celite pad and concentrated *in vacuo*. To the crude solid water (100 mL) was added and the solid was filtered off under reduced pressure, washed with water and dried in air. The crude product was recrystallized from methanol which afforded 508 mg (22 %) of pure **2** as a white powder.

^{1}H NMR (400 MHz, DMSO-d_6) δ/ppm: 8,42 (d, J = 7,5 Hz, 1H), 8,37 (t, J = 5,6 Hz, 1H), 8,04 (t, J = 5,7 Hz, 1H), 7,97 (d, J = 7,9 Hz, 1H), 7,28–7,14 (m, 15H), 4,58–4,57 (m, 1H), 4,37–4,32 (m, 1H), 3,79–3,62 (m, 4H), 3,54 (dd, J_1 = 8,4 Hz, J_2 = 4,9 Hz, 1H), 3,09–3,03 (m, 2H), 2,91–2,83 (m, 3H), 2,58 (dd, J_1 = 13,7 Hz, J_2 = 8,5 Hz, 1H).

^{13}C NMR (100 MHz, DMSO-d_6) δ/ppm: 173,1, 172,3, 171,2, 168,8, 168,3, 138,0, 137,6, 129,4, 129,3, 129,2, 128,2, 128,1, 126,5, 126,3, 126,2, 55,5, 54,2, 53,6, 42,1, 41,8, 39,4, 37,6, 37,1.

HRMS (ESI) m/z $[M + H]^+$ – calculated ($C_{31}H_{35}N_5O_6^+$) – 574,2621, found 574,2670.

1.4. Synthesis of hexapeptide H-Phe-Phe-Gly-Gly-Phe-Phe-OH (3)

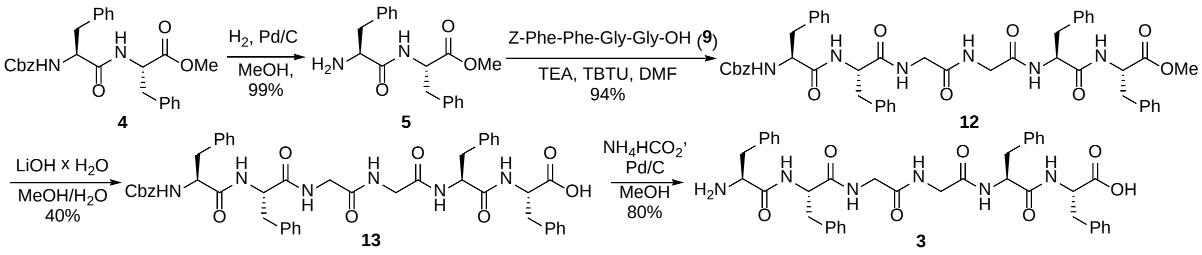

Synthesis of L-phenylalanyl-L-phenylalanine methyl ester (5)

Compound **4** (2.80 g, 6.1 mmol, 1 equiv) was dissolved in methanol (250 mL), Pd/C was added and the reaction mixture was stirred under an atmosphere of H_2 at room temperature for 48 h. The reaction mixture was heated, filtered over a celite pad and concentrated *in vacuo*. 1.98 g (> 99 %) of **5** was obtained as a pale-yellow solid and was used in the next step without further purification. ^{1}H NMR spectrum is in accordance with the previously reported spectrum of the compound.[3]

Synthesis of *N*-(carbobenzyloxy)-L-phenylalanyl-L-phenylalanylglycylglycyl-L-phenylalanyl- L-phenylalanine methyl ester (12)

Compounds **9** (2.35 g, 4.2 mmol, 1 equiv) and **5** (1.37 g, 4.2 mmol, 1 equiv) were dissolved in DMF (75 mL). To the cooled mixture (0 °C) were added TEA (1.17 mL, 8.4 mmol, 2 equiv), then TBTU (1.48 g, 4.6 mmol, 1.1 equiv) and the reaction mixture was stirred at room temperature overnight. The mixture was cooled to 0 °C and water (200 mL) was added dropwise. The mixture was transferred to a separatory funnel and extracted with DCM (3×100 mL). The organic extracts were combined, washed with HCl (100 mL, c = 1 mol dm^{-3}), brine (100 mL), saturated $NaHCO_3$ solution (100 mL), and finally with brine (100 mL). The organic phase was dried over Na_2SO_4, filtered and the volume was reduced under reduced pressure. The precipitate was filtered, washed with DCM and dried in air. 1.03 g of pure **12** was obtained as a white powder. The filtrate was concentrated *in vacuo*, dissolved in MeOH/DCM, SiO_2 was added and the mixture was concentrated *in vacuo*. The mixture was dry-loaded and purified by column chromatography (2,5 % to 5 % MeOH in DCM). Another 2.40 g was obtained, amounting to a total of 3.43 g (94%) of **12**.

^{1}H NMR (400 MHz, DMSO-d_6) δ/ppm: 8,50 (d, J = 7,5 Hz, 1H), 8,25 (t, J = 5,2 Hz, 1H), 8,14 (d, J = 7,9 Hz, 1H), 8,05 (d, J = 8,4 Hz, 1H), 7,93 (t, J = 5,0 Hz, 1H), 7,45 (d, J = 8,7 Hz, 1H), 7,34–7,01 (m, 25H), 4,99–4,77 (m, 2H), 4,57–4,45 (m, 3H), 4,26 – 4,21 (m, 1H), 3,75 3,70 (m, 3H), 3,56 (s, 3H), 3,08 – 2,63 (m, 8H).

^{13}C NMR (100 MHz, DMSO-d_6) δ/ppm: 171,7, 171,4, 171,2, 171,1, 168,8, 168,2, 155,7, 138,0, 137,6, 137,0, 129,3, 129,2, 129,1, 128,3, 128,0, 127,6, 127,4, 126,6, 126,3, 126,2, 65,2, 56,1, 53,8, 53,7, 53,5, 51,8, 42,0, 41,7, 37,6, 37,4, 36,6.

HRMS (ESI) m/z [M + H]$^+$ – calculated ($C_{49}H_{53}N_6O_9^+$) – 722,3145, found 722,3195.

Synthesis of *N*-(carbobenzyloxy)-L-phenylalanyl-L-phenylalanylglycylglycyl-L-phenylalanyl- L-phenylalanine (13)

Compound **12** (2.23 g, 2.6 mmol, 1 equiv) was suspended in methanol (150 mL), the mixture was heated until everything dissolved and added to the stirred solution LiOH · H_2O (130 mg, 3.1 mmol, 1.2 equiv) and water (40 mL). The reaction mixture was stirred at room temperature for 48 h. More LiOH · H_2O (108 mg, 2.6 mmol, 1 equiv) was added, the reaction mixture was heated until everything dissolved and stirred at room temperature for another 48 h. Methanol was evaporated under reduced pressure, the mixture was cooled to 0 °C and HCl(10,0 mL, c = 1 mol dm^{-3}) was added dropwise. Contents were transferred to a separatory funnel and extracted with EtOAc (300 mL). The organic extract was washed with brine (100 mL) and

concentrated *in vacuo*. The crude product was dissolved in EtOAc, SiO_2 was added, concentrated *in vacuo*, dry-loaded and purified by column chromatography (5 % to 10 % MeOH in DCM). 885 mg (40 %) of nearly pure **13** was obtained as a white powder and was used in the next step without further purification.

^{1}H NMR (400 MHz, DMSO-d_6) δ/ppm: 12,74 (brs, 1H), 8,34 (d, J = 7,8 Hz, 1H), 8,25 (t, J = 5,5 Hz, 1H), 8,17 (d, J = 7,5 Hz, 1H), 8,04 (d, J = 8,5 Hz, 1H), 7,92 (t, J = 5,5 Hz, 1H), 7,46 (d, J = 8,7 Hz, 1H), 7,34–7,03 (m, 25H), 4,96–4,77 (m, 2H), 4,60–4,52 (m, 2H), 4,46–4,40 (m, 1H), 4,27–4,21 (m, 1H), 3,75–3,70 (m, 3H), 3,59 (dd, J_1 = 16,7 Hz, J_2 = 5,5 Hz, 1H), 3,09–2,63 (m, 8H).

^{13}C NMR (100 MHz, DMSO-d_6) δ/ppm: 172,7, 171,4, 171,2, 171,0, 168,8, 168,2, 155,7, 138,0, 137,7, 137,6, 137,4, 137,0, 129,3, 129,22, 129,16, 129,1, 128,3, 128,2, 128,0, 127,6, 127,4, 126,4, 126,2, 65,2, 56,1, 53,9, 53,6, 42,0, 41,7, 37,6, 37,4, 36,7.

HRMS (ESI) *m/z* [M + H]$^+$ – calculated ($C_{48}H_{51}N_6O_9^+$) – 855,3673, found 855,3727.

Synthesis of L-phenylalanyl-L-phenylalanylglycylglycyl-L-phenylalanyl- L-phenylalanine (3)

Compound **13** (727 mg, 0.85 mmol, 1 equiv) and ammonium formate (536 mg, 8.5 mmol, 10 equiv) were dissolved in methanol (150 mL), Pd/C was added and the mixture was refluxed for 2.5 hours. The hot reaction mixture was filtered over a celite pad and concentrated *in vacuo*. To the crude mixture, water (50 mL) was added and the solid was filtered off under reduced pressure, washed with water and dried in air. The crude product was triturated in methanol. 493 mg (80 %) of pure **3** was obtained as a white powder.

^{1}H NMR (400 MHz, DMSO-d_6) δ/ppm: 9,26 (brs, 1H), 8,42 (t, J = 5,5 Hz, 1H), 8,27 (d, J = 8,3 Hz, 1H), 7,99 (t, J = 5,7 Hz, 1H), 7,94 (d, J = 7,0 Hz, 1H), 7,29–7,14 (m, 20H), 4,53–4,47 (m, 1H), 4,43–4,37 (m, 1H), 4,29 (m, 1H), 3,79 (dd, J_1 = 16,5 Hz, J_2 = 6,3 Hz, 1H), 3,69–3,58 (m, 4H), 3,15 (dd, J_1 = 13,5 Hz, J_2 = 5,2 Hz, 1H), 3,07–2,90 (m, 5H), 2,75–2,63 (m, 2H).

^{13}C NMR (100 MHz, DMSO-d_6) δ/ppm: 173,5, 171,3, 171,2, 170,5, 168,9, 168,6, 138,1, 138,0, 137,9, 136,9, 129,42, 129,39, 129,3, 129,1, 128,3, 128,1, 128,0, 126,5, 126,3, 126,25, 126,15, 55,1, 54,7, 54,6, 54,3, 42,2, 41,9, 37,3, 37,0.

HRMS (ESI) *m/z* [M + H]$^+$ – calculated ($C_{40}H_{45}N_6O_7^+$) – 721,3305, found 721,3358.

1.5. Chloride-assisted macrocyclizations of peptides 2 and 3 – solution-based approach

Synthesis of cyclo-L-phenylalanyl-L-phenylalanyl-L-phenylalanyl-glycylglycine (C2)

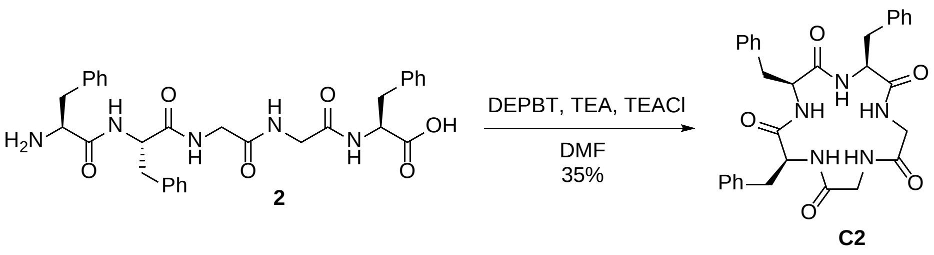

To a solution of **2** (284 mg, 0.5 mmol, 1 equiv) and DEPBT (163 mg, 0.55 mmol, 1.1 equiv) in DMF (300 mL), TEACl (1.23 g, 7.4 mmol, 15 equiv) and TEA (140 µL, 1.00 mmol, 2 equiv) were added and the reaction mixture was stirred at room temperature for 4 days. The mixture was concentrated *in vacuo*, then EtOAc (100 mL) and water (100 mL) were added and the bilayer system was stirred for 10 minutes. The precipitate was filtered off, washed with water and dried. The filtrate was transferred to a separatory funnel, the layers were separated and the organic layer was dried over Na_2SO_4, filtered and concentrated *in vacuo*. The precipitate and extract were combined, dry-loaded and purified by column chromatography (5 % to 10 % MeOH in DCM). 96 mg (35 %) of pure **C2** was obtained as a white powder. The ^{1}H NMR spectrum is in accordance with the previously reported spectrum of the compound.[4]

^{1}H NMR (400 MHz, DMSO-d_6) δ/ppm: 8,65 (t, J = 5,8 Hz, 1H), 8,46 (d, J = 7,6 Hz, 1H), 8,11 (d, J = 7,5 Hz, 1H), 7,99 (d, J = 8,7 Hz, 1H), 7,67 (m, 1H), 7,29–7,17 (m, 11H), 7,12–7,06 (m, 4H), 4,36–4,24 (m, 2H), 4,19 (m, 1H), 3,90 (dd, J_1 = 15,6 Hz, J_2 = 6,6 Hz, 1H), 3,72 (dd, J_1 = 14,8 Hz, J_2 = 6,7 Hz, 1H), 3,72 (dd, J_1 = 14,8 Hz, J_2 = 6,7 Hz, 1H), 3,60 (dd, J_1 = 14,8 Hz, J_2 = 4,6 Hz, 1H), 3,40 (dd, J_1 = 15,6 Hz, J_2 = 5,2 Hz, 1H), 3,08 (dd, J_1 = 13,6 Hz, J_2 = 6,9 Hz 1H), 2,98–2,88 (m, 3H), 2,82–2,72 (m, 2H).

^{13}C NMR (100 MHz, DMSO-d_6) δ/ppm: 171,1, 170,5, 169,3, 169,0, 137,8, 137,4, 137,3, 129,15, 129,10, 128,8, 128,24, 128,18, 126,4, 126,3, 56,4, 56,1, 54,9, 43,2, 42,7, 37,5, 36,7, 36,5.

HRMS (ESI) m/z $[M + Na]^+$ – calculated ($C_{31}H_{33}N_5O_5Na^+$) – 578,2374, found 578,2386.

Synthesis of cyclo-L-phenylalanyl-L-phenylalanyl-L-phenylalanyl-L-phenylalanyl-glycylglycine (C3)

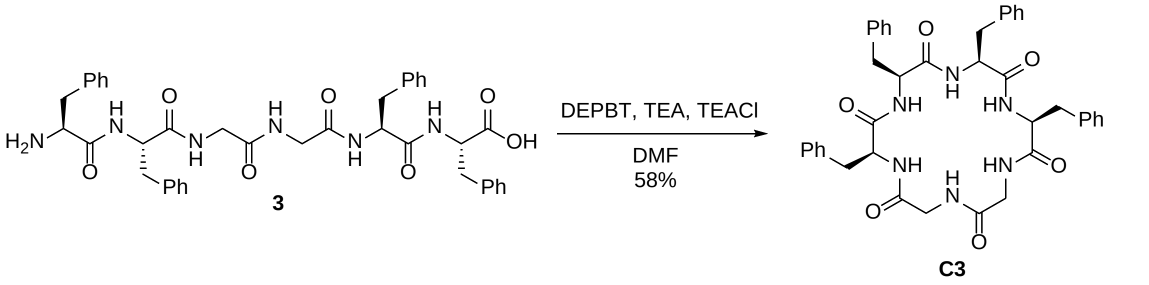

To a solution of **3** (300 mg, 0.42 mmol, 1 equiv) and DEPBT (137 mg, 0.46 mmol, 1.1 equiv) in DMF (300 mL), TEACl (1.03 g, 6.2 mmol, 15 equiv) and TEA (120 µL, 0.86 mmol, 2 equiv) were added and the reaction mixture was stirred at room temperature for 3 days. The mixture was concentrated *in vacuo*, then EtOAc (100 mL) and water (100 mL) were added and the bilayer system was stirred for 15 minutes, transferred to a separatory funnel and the layers were separated. The water layer was extracted with EtOAc (50 mL), combined organic extracts were washed with brine (3×50 mL), dried over Na_2SO_4, filtered and concentrated *in vacuo*. The crude solid was triturated in MeOH which afforded 112 mg of pure **C3**. The filtrate was treated with an anion-exchange resin, filtered, concentrated *in vacuo* and purified by column chromatography (5 % to 10 % MeOH in DCM) which afforded another 58 mg of pure **C3**. A total of 170 mg (58 %) of pure **C3** was obtained as a white powder.

^{1}H NMR (400 MHz, DMSO-d_6) δ/ppm: 8,35–8,32 (m, 1H), 8, 27 (d, J = 7,9 Hz, 1H), 8,20–8,18 (m, 1H), 8,03–7,95 (m, 3H), 7,30–7,06 (m, 20H), 4,37–4,30 (m, 3H), 4,22–4,17 (m, 1H), 3,94 (dd, J_1 = 16,6 Hz, J_2 = 7,0 Hz, 1H), 3,69 (dd, J_1 = 15,5 Hz, J_2 = 7,2 Hz, 1H), 3,60 (dd, J_1 = 15,5 Hz, J_2 = 4,0 Hz, 1H), 3,42 (dd, J_1 = 16,5 Hz, J_2 = 3,8 Hz, 1H), 3,12–2,58 (m, 8H)

^{13}C NMR (100 MHz, DMSO-d_6) δ/ppm: 171,3, 171,2, 170,9, 170,7, 169,3, 168,8, 137,9, 137,5, 137,1, 129,3, 129,1, 129,0, 128,7, 128,3, 128,2, 128,1, 126,5, 126,3, 55,3, 54,9, 54,8, 42,64, 42,59, 37,3, 36,5, 35,8

HRMS (ESI) m/z [M + Na]$^+$ – calculated ($C_{40}H_{42}N_6O_6Na^+$) – 725,3058, found 725,3073.

2. Mechanochemical macrocyclizations of oligopeptides

2.1. General procedure for the mechanochemical macrocyclizations of oligopeptides

Peptide **A** (1 mmol), chloride salt **B** (15 mmol), K_2CO_3 (2 mmol) (and DMF (10 µL) in case of LAG) were added to a steel jar (5 mL internal volume), two stainless steel balls (5 mm in diameter) were added and the contents were ground in a vibratory ball mill 45 min at a frequency of 30 Hz. DEPBT (1,1 mmol) was added and the contents were ground again for another 120 min at 30 Hz. A sample of known mass m_s was taken directly from the jar, UV/VIS grade DMSO (500 µL) and HPLC grade MeOH (500 µL) were added, the sample was sonicated, filtered into an HPLC vial and analysed by reversed phase HPLC. The HPLC utilized a gradient method (Table 1) using a 10 % HPLC grade ACN solution in miliQ water (**S1**) and HPLC grade ACN (**S2**). The prepared samples (10 µL) were analysed on a Waters Symmetry C8 5 µm column (4,6 x 150 mm) set at a constant temperature of 25 °C. Calibration curves were made by HPLC analysis using standard samples of the pure corresponding cyclopeptides (Figure S2). The concentration *c* of the corresponding cyclopeptide was determined by linear regression from the calibration curves and the grinding experiment yield was calculated as:

$$\eta = \frac{cVM(A)}{m(A)} \times \frac{m}{m_s}$$

Where *V* is the volume of the prepared sample (1 mL), *m*(A) and *M*(A) are the mass and molar mass, respectively, of the linear peptide **A**, *m* is the sum of masses of the contents added to the jar and m_s is the mass of the sample taken from the jar after the grinding experiment.

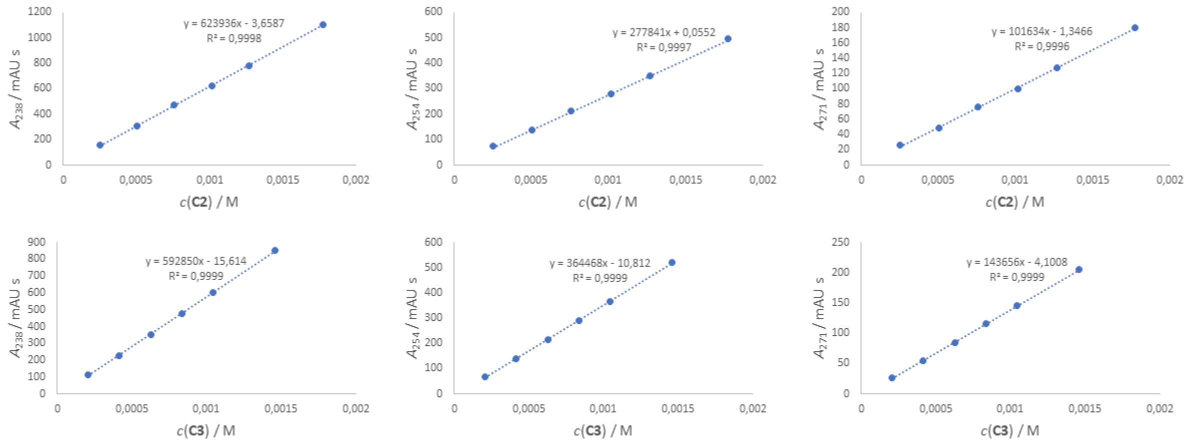

Figure S2. The calibration curves obtained by HPLC analysis of standard samples of C2 (retention time 7.991 min) and C3 (retention time 8.692 min) shown as area *A* of the signal on the detector over concentration *c* of the cyclopeptide. Calibration was done at three different wavelengths: 238 nm, 254 nm and 271 nm.

Table S1. The gradient HPLC method used to analyse samples of the grinding experiments shown as the volume fraction of **S2** (ACN) in the mobile phase comprised of a mixture of 9:1 H_2O:ACN (**S1**) and ACN (**S2**).

t/min	% **S2**
0	10
12	90
18	90
22	10

Table S2. Optimization of reaction time after addition of DEPBT monitored by HPLC in ball-milling experiment according to general procedure described in 2.1 with peptide **2**, and salt benzyltriethylammonium chloride.

t/min	Yield (HPLC)
30	13 %
60	20 %
90	21 %
120	23 %
150	23 %
180	23 %

Table S3. Experimental conditions for the grinding experiments of peptides **2** and **3** and yields of the corresponding cyclopeptides **C2** and **C3** respectively determined by HPLC

Entry	Peptide	Salt	eq K_2CO_3	LAG/NG	Yield (HPLC)
E1	**2**	TEACl (15 eq)	2	LAG (DMF)	18 %
E2	**2**	NaCl (15 eq)	2	LAG (DMF)	4 %
E3	**2**	TEACl (15 eq)	2	NG	24 %
E4	**2**	KCl (15 eq)	2	NG	6 %
E5	**2**	KCl (15 eq)	2	LAG (DMF)	3 %
E6	**2**	$BnNEt_3Cl$ (15 eq)	2	NG	39 %
E7	**2**	$TBAHSO_4$ (15 eq)	2	NG	16 %
E8	**2**	$BnNEt_3Cl$ (15 eq)	4	NG	31 %
E9	**2**	$BnNEt_3Cl$ (15 eq)	6	NG	33 %
E10	**2**	$CaCl_2$ (15 eq)	2	NG	5 %
E11	**2**	$CaCl_2$ (15 eq)	4	NG	3 %
E12	**2**	TEACl (20 eq)	2	NG	27 %
E13	**2**	TEACl (25 eq)	2	NG	27 %
E14	**2**	TEACl (30 eq)	2	NG	30 %
E15	**2**	TEACl (5 eq)	2	NG	28 %
E16	**2**	TEACl (10 eq)	2	NG	30 %
E17	**3**	TEACl (15 eq)	2	NG	20 %
E18	**3**	TEACl (15 eq)	4	NG	23 %
E19	**3**	$BnNEt_3Cl$ (15 eq)	2	NG	22 %
E20	**3**	$BnNEt_3Cl$ (15 eq)	4	NG	25 %
E21	**2**	$BnNEt_3Cl$ (15 eq)	2	LAG (EtOAc)	18 %
E22	**2**	$BnNEt_3Cl$ (15 eq)	2	LAG (H_2O)	2 %
E23	**2**	$BnNEt_3Cl$ (15 eq)	2	LAG (DMSO)	11 %
E24	**2**	$BnNEt_3Cl$ (15 eq)	2	LAG (dioxane)	14 %

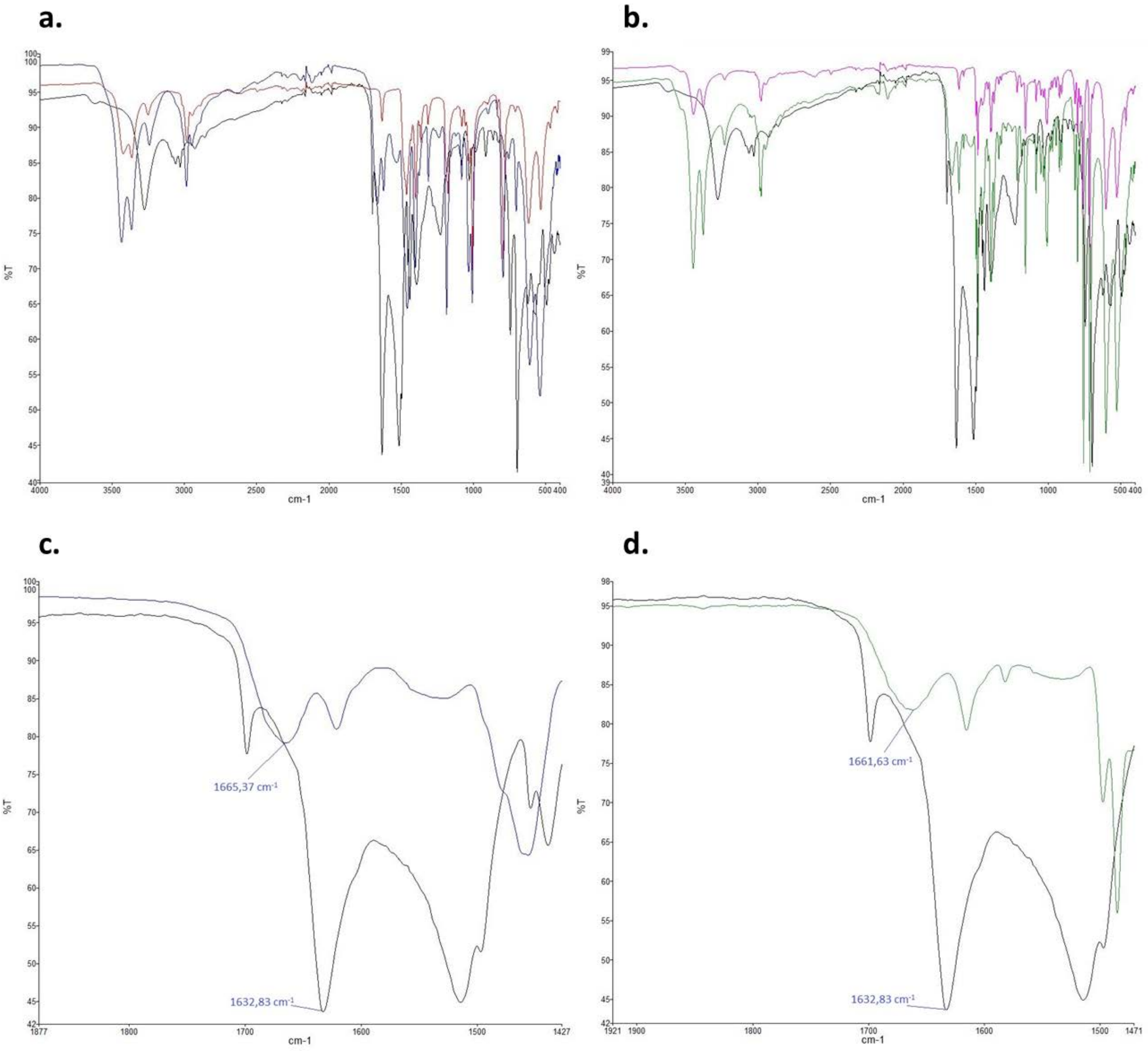

Figure S3. IR spectra of **a.** compound **2** (black), TEACl (red) and a sample after grinding **2** with TEACl (blue), **b.** compound **2** (black), benzyltriethylammonium chloride (magenta) and a sample after grinding **2** with benzyltriethylammonium chloride (green). **c.** Carbonyl group stretch bands in **2** (black) and sample after grinding **2** with TEACl (black) and **d.** carbonyl group stretch bands in **2** (black) and sample after grinding **2** with benzyltriethylammonium chloride (green).

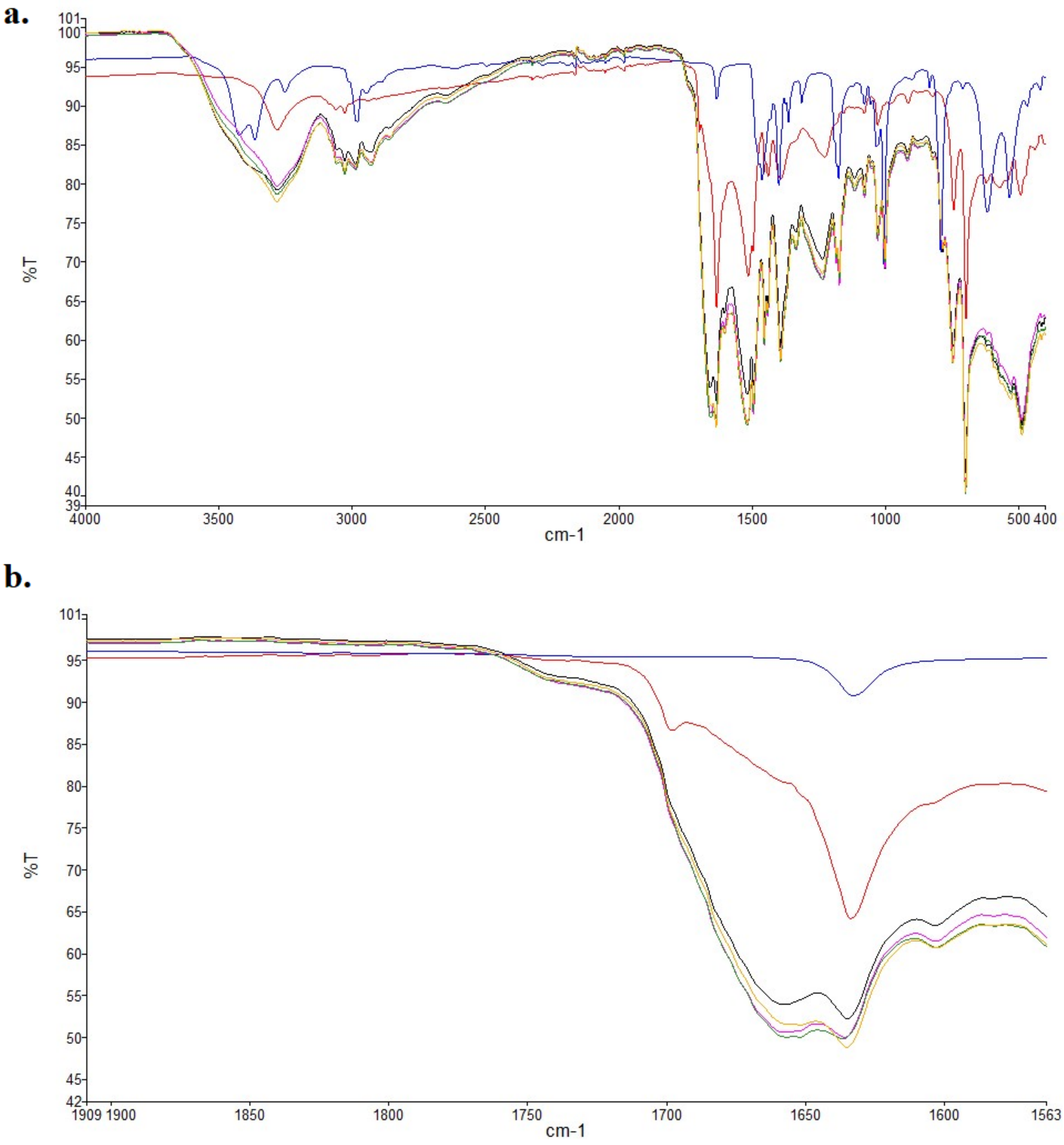

Figure S4. Optimization of complexation time monitored by recording IR spectra of **a.** TEACl (blue), peptide **2** (red), samples after grinding **2** with **1 equiv. TEACl** for 15 min (black), 30 min (magenta), 45 min (green) and 60 min (yellow), **b.** Carbonyl group stretch band region of the IR spectra shown in **a**.

2.2. Synthesis of cyclo-L-phenylalanyl-L-phenylalanyl-L-phenylalanyl-glycylglycine (C2)

Linear peptide **2** (14.5 mg, 0.02 mmol, 1 equiv), benzyltriethylammonium chloride (68 mg, 0.3 mmol, 15 equiv) and K_2CO_3 (6 mg, 0.04 mmol, 2 equiv) were added to a steel jar (5 mL internal volume), two stainless steel balls (5 mm in diameter) were added and the contents were ground in a vibratory ball mill 45 min at a frequency of 30 Hz. DEPBT (7 mg, 0.022 mmol, 1.1 equiv) was added and the contents were ground again for another 120 min at 30 Hz. A sample was

analyzed by reversed phase HPLC, as described in general method, and the yield of 39 % was calculated which corresponds to 4.3 mg (0.008 mmol) of **C2**.

2.3. Synthesis of cyclo-L-phenylalanyl-L-phenylalanyl-L-phenylalanyl-L-phenylalanyl -glycylglycine (C3)

Linear peptide **3** (11.5 mg, 0.02 mmol, 1 equiv), benzyltriethylammonium chloride (68 mg, 0.3 mmol, 15 equiv) and K_2CO_3 (11 mg, 0.08 mmol, 4 equiv) were added to a steel jar (5 mL internal volume), two stainless steel balls (5 mm in diameter) were added and the contents were ground in a vibratory ball mill 45 min at a frequency of 30 Hz. DEPBT (7 mg, 0.022 mmol, 1.1 equiv) was added and the contents were ground again for another 120 min at 30 Hz. A sample was analyzed by reversed phase HPLC, as described in general method, and the yield of 25 % was calculated which corresponds to 3.5 mg (0.005 mmol) of **C3**.

2.4. Calculation of E-factor for macrocyclization step

Calculation of the environmental impact factor (E-factor) was performed as described in other publications.[5,6]

$$\mathrm{E-factor} = \frac{\text{total mass of all reactants and solvents used} - \text{mass of desired product}}{\text{mass of desired product}}$$

**Solution-based approach**			**Mechanochemical approach**		
Mass of reactants and solvents		*Mass of the desired product*	*Mass of reactants*		*Mass of the desired product*
Linear precursor	284 mg		Linear precursor	14.5 mg	
DEPBT	163 mg		DEPBT	7 mg	
TEACl	1230 mg		$BnNEt_3Cl$	68 mg	
TEA	140 mg		K_2CO_3	6 mg	
DMF	283 200 mg		-		
**Total**	**285 017 mg**	**96 mg**	**Total**	**95.5 mg**	**4.3 mg**
***E-factor 2968**			***E-factor 21**		

*E-factor is calculated for the macrocyclization of linear precursor **2**. No work-up nor purification step are taken into account.

2.5. Procedure for the scaled-up mechanochemical cyclization of peptide 2

2 (0.35 g, 0.61 mmol, 1 equiv), benzyltriethylammonium chloride (2.08 g, 9.15 mmol, 15 equiv) and K_2CO_3 (0.17 g, 1.2 mmol, 2 equiv) were added to a steal jar (50 mL internal volume), 10 stainless steel balls (15 mm in diameter) were added and the contents were ground 30 min (650 rpm) in a Retsch PM 200 planetary ball mill. The mixture was allowed to cool to room temperature and DEPBT (0.20 g, 0.67 mmol, 1.1 equiv) was added and the contents were ground for another 30 minutes (650 rpm). HPLC analysis of a sample of the mixture showed a 20 % yield. The mixture was cooled to room temperature, SiO_2 was added, ground for 15 minutes, the contents were dry-loaded on a silica plug and eluted with 10% MeOH in DCM. The cyclopeptide was purified by column chromatography (5 % to 10 % MeOH in DCM). 61 mg (18 %) of **C2** was obtained as a white powder.

3. Procedure for the molecular dynamics simulations of C2 complexes

The MD simulations of **C2** chloride complexes with sodium or tetraethylammonium counterions in vacuum were performed by GROMACS package (version 2022.5).[7-13] Optimized Parameters for Liquid Simulations-All Atoms (OPLS-AA) force field was used for modelling of intramolecular and nonbonded intermolecular interactions.[14] Tetraethylammonium cation force field parameters were obtained by LigParGen webserver.[15-17] The cyclopeptide **C2** chloride complexes along with appropriate counterion in their proximity were situated at the centre of cubical boxes (side length of 70 Å), with periodic boundary conditions. In all simulations an energy minimization procedure was performed followed by a MD simulation in NVT conditions for 25.5 ns, where the first 0.5 ns was not used in data analysis. The propagation of positions and momenta was performed by the Verlet algorithm[18] with a time step of 1 fs. The cut-off radius for nonbonded van der Waals and short-range Coulomb interactions was 16 Å. Ewald method implemented in the Particle Mesh Ewald (PME) procedure was used for the description of Long-range Coulomb interactions.[19] The simulation temperature was kept constant at 298 K with the help of Nosé-Hoover algorithm[20,21] using a time constant of 0.2 ps. Representative molecular structures of cyclopeptide-anion complexes presented on Figure S4 were obtained by Principle Component Analysis (PCA) on coordination matrix whose rows contained distances between chloride anion and amide protons and angles between chloride anion and amide proton donor atoms. In the case of sodium

chloride, the coordination matrix also contained distances between sodium cation and carbonyl oxygen atoms and angles between the cation and carbonyl groups. The representative structures were those closest to the centroids of the most populous clusters in space defined by the first three principal components. Figures of molecular structures were rendered using VMD software.[22]

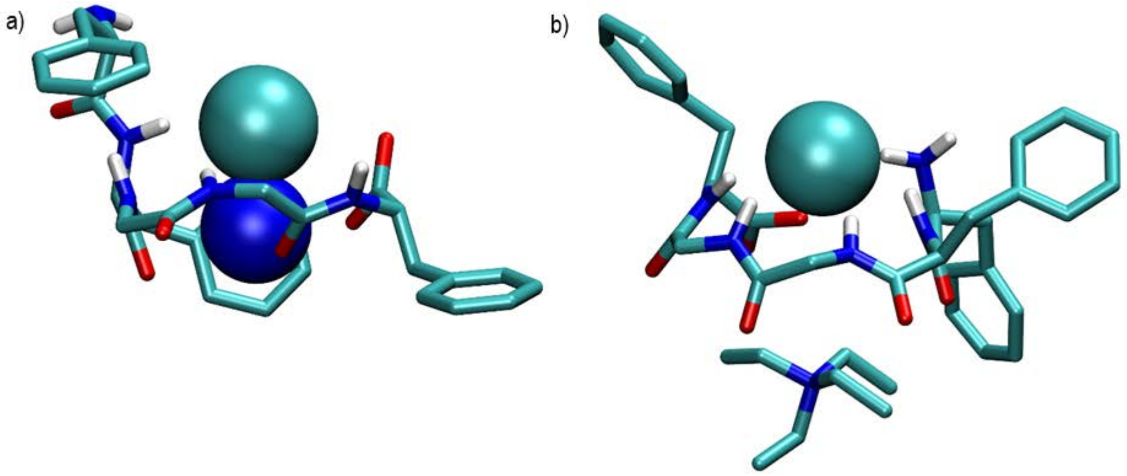

Figure S5. Representative structures of **C2** complexes with a) Na (blue sphere) – Cl (green sphere) ion pair, b) TEA-Cl ion pair obtained by vacuum MD simulations.

4. References

1. R. R. Hill, D. Birch, G. E. Jeffs and M. North, *Org. Biomol. Chem.*, 2003, **1**, 965–972.
2. A. R. Katritzky, K. Suzuki and S. K. Singh, *Synth.*, 2004, **16**, 2645–2652.
3. S. Bayryamov, D. Danalev and N. Vassilev, *Phosphorus Sulfur Silicon Relat. Elem.*, 2011, **186**, 338–344.
4. Y. A. Bara, A. Friedrich, W. Hehlein, H. Kessler, P. Kondor, M. Molter and H.-J. Veith, *Chem. Ber.*, 1978, **111**, 1029–1044.
5. D. J. C. Constable, A. D. Curzons and V. L. Cunnigham, *Green Chem.*, 2002, **4**, 521–527.
6. E. Martinez-Guerra and V. G. Gude, *Appl. Sci.*, 2017, **7**, 869.
7. H. J. C. Berendsen, D. van der Spoel and R. van Drunen, *Comput. Phys. Commun.*, 1995, **91**, 43–56.
8. E. Lindahl, B. Hess and D. van der Spoel, *J. Mol. Model.*, 2001, **7**, 306–317.
9. D. Van Der Spoel, E. Lindahl, B. Hess, G. Groenhof, A. E. Mark and H. J. C. Berendsen, *J. Comput. Chem.*, 2005, **26**,1701–1718.
10. B. Hess, C. Kutzner, D. van der Spoel and E. Lindahl, *J. Chem. Theory Comput.*, 2008, **4**, 435–447.
11. S. Pronk, S. Páll, R. Schulz, P. Larsson, P. Bjelkmar, R. Apostolov, M. R. Shirts, J. C. Smith, P. M. Kasson, D. van der Spoel, B. Hess and E. Lindahl, *Bioinformatics*, 2013, **29**, 845–854.
12. M. J. Abraham, T. Murtola, R. Schulz, S. Páll, J. C. Smith, B. Hess and E. Lindahl, *SoftwareX*, 2015, **1–2**, 19–25.
13. S. Páll, M. J. Abraham, C. Kutzner, B. Hess and E. Lindahl, Tackling Exascale Software Challenges in Molecular Dynamics Simulations with GROMACS, *Lect. Notes Comput. Sci.*, 2015, 3–27.
14. W. L. Jorgensen, D. S. Maxwell and J. Tirado-Rives, *J. Am. Chem. Soc.*, 1996, **118**, 11225–11236.
15. W. L. Jorgensen and J. Tirado-Rives, *Proc. Natl. Acad. Sci.*, 2005, **102**, 6665–6670.
16. L. S. Dodda, I. Cabeza de Vaca, J. Tirado-Rives and W. L. Jorgensen, *Nucleic Acids Res.*, 2017, **45**, W331–W336.
17. L. S. Dodda, J. Z. Vilseck, J. Tirado-Rives and W. L. Jorgensen, *J. Phys. Chem. B*, 2017, **121**, 3864–3870.
18. W. C. Swope, H. C. Andersen, P. H. Berens and K. R. Wilson, *J. Chem. Phys.*, 1982, **76**, 637–649.
19. T. Darden, D. York and L. Pedersen, *J. Chem. Phys.*, 1993, **98**, 10089–10092.
20. S. Nosé, *Mol. Phys.*, 1984, **52**, 255–268.
21. W. Hoover, *Phys. Rev. A*, 1985, **31**, 1695–1697.
22. W. Humphrey, A. Dalke and K. Schulten, *J. Mol. Graph.*, 1996, **14**, 33–38.

5. NMR and HRMS spectra

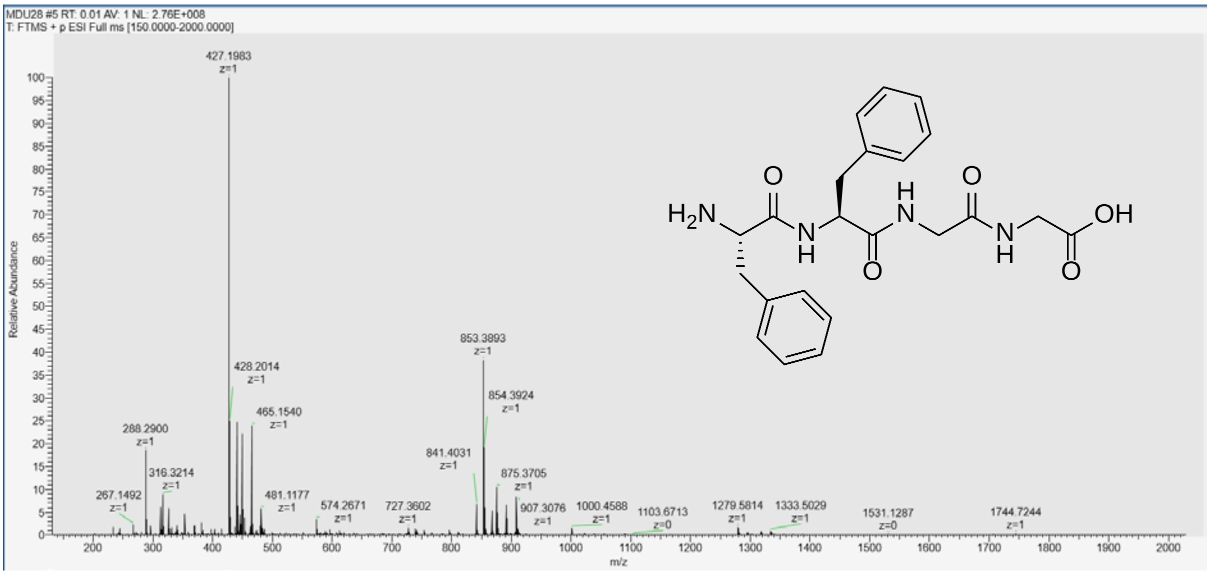

Figure S6. HRMS spectrum of compound **1**.

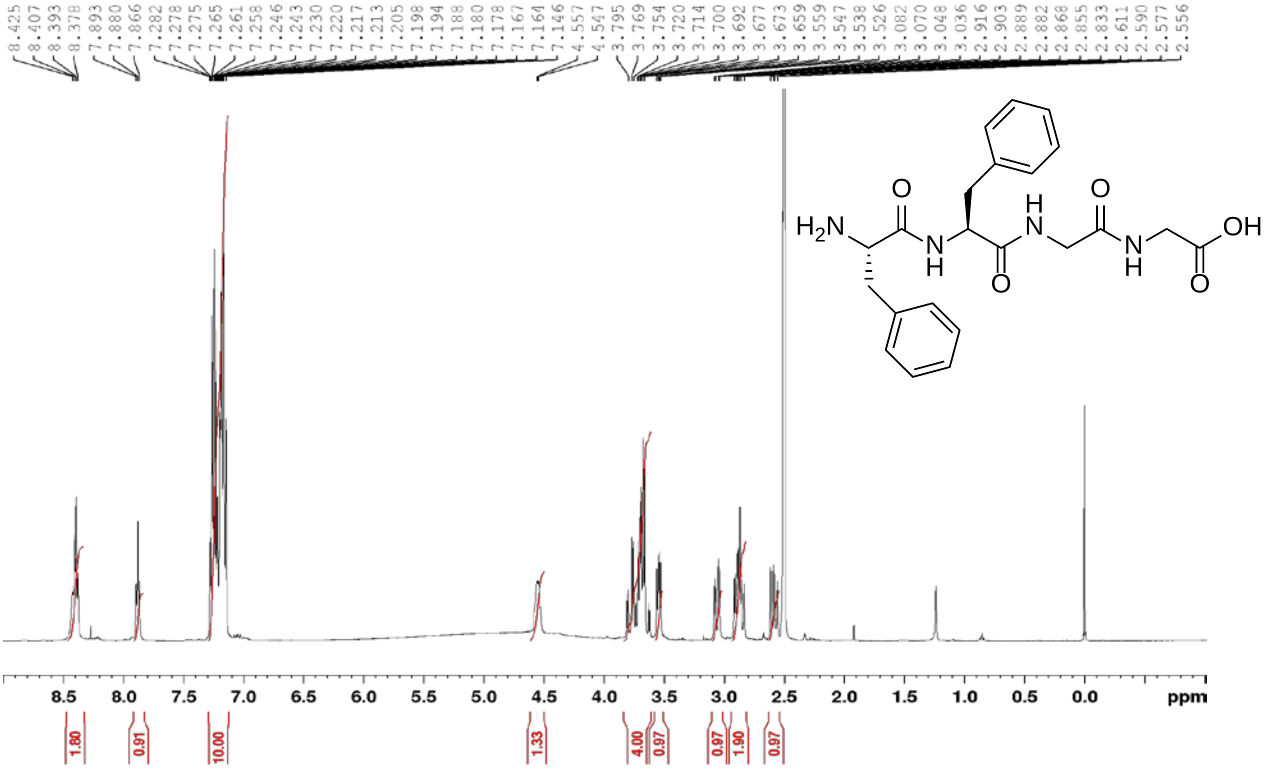

Figure S7. ^{1}H NMR spectrum of compound **1** in DMSO-d_6.

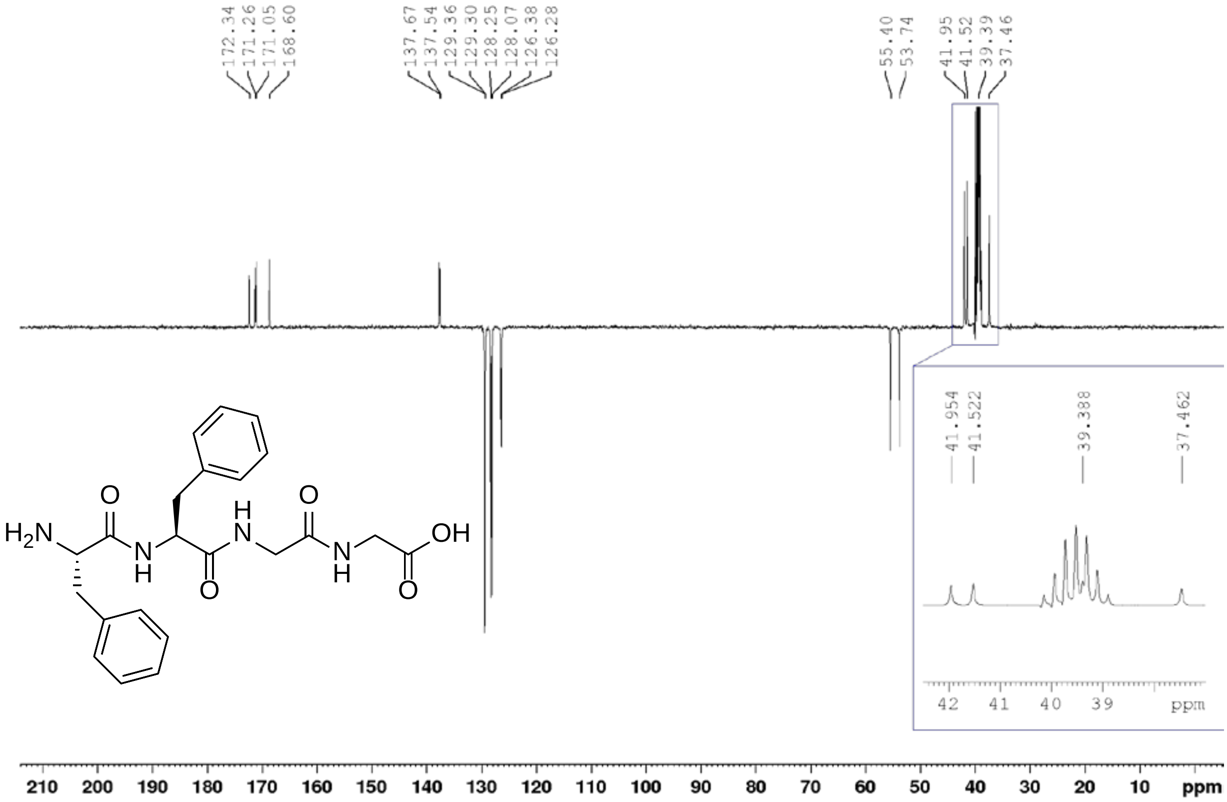

Figure S8. ^{13}C NMR spectrum of compound **1** in DMSO-d_6.

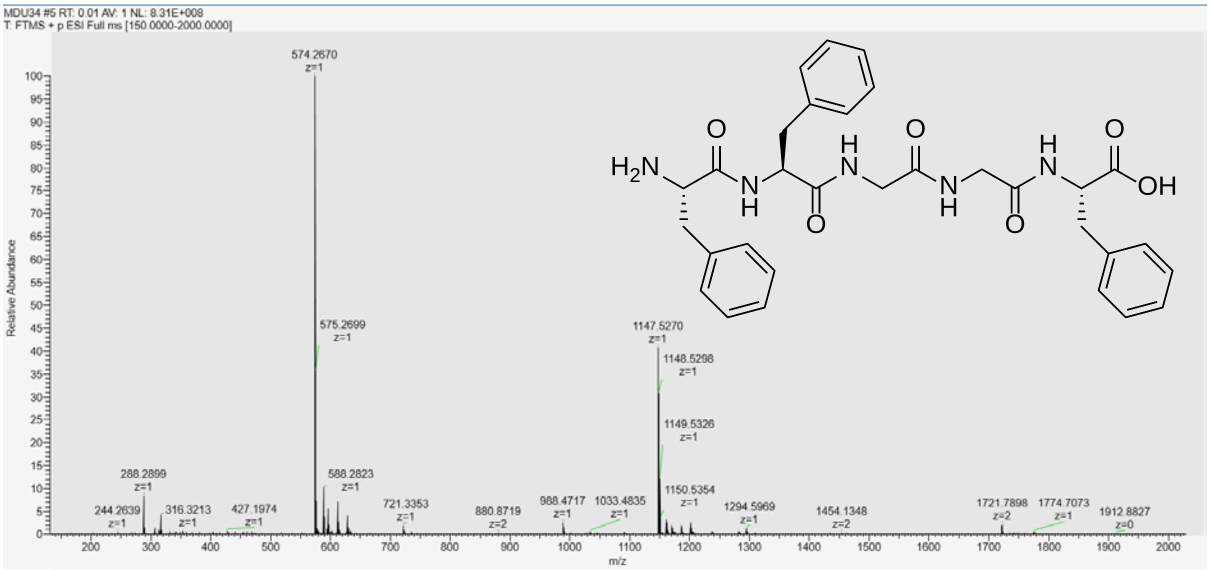

Figure S9. HRMS spectrum of compound **2**.

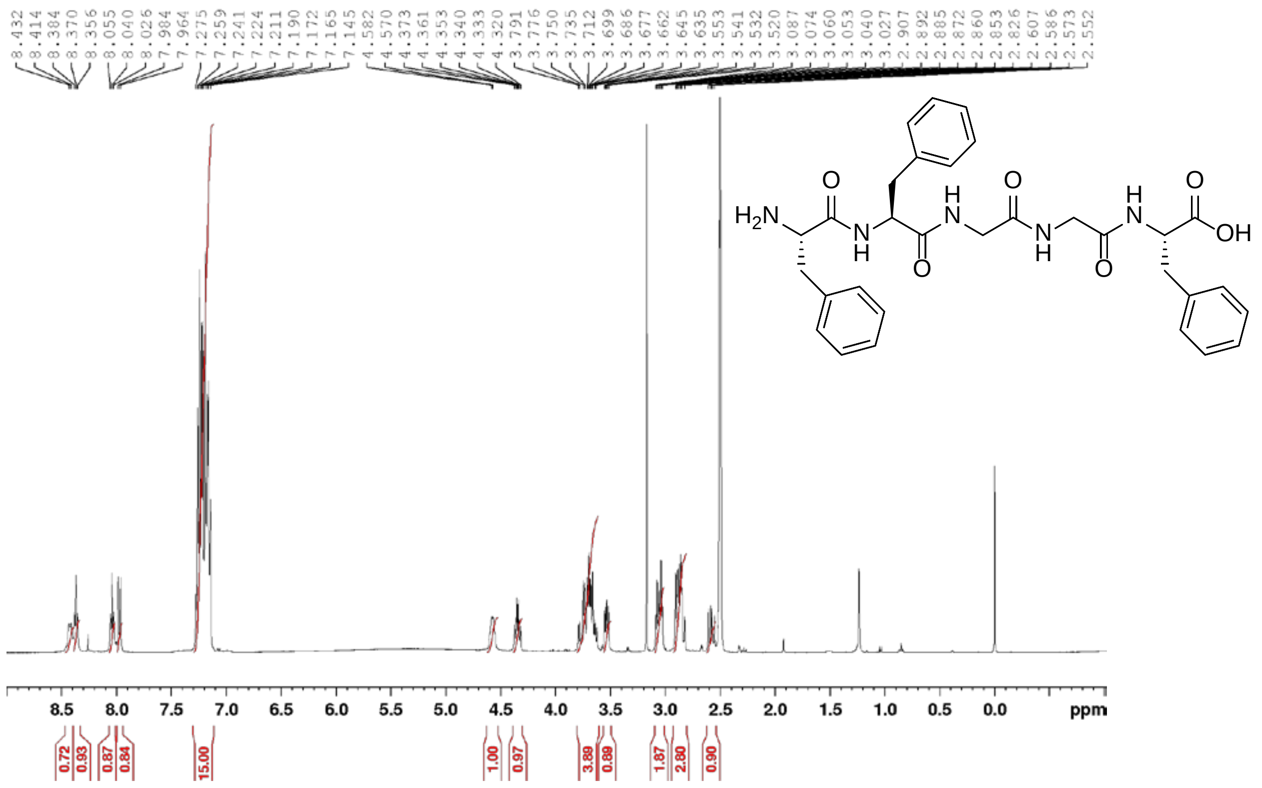

Figure S10. ^{1}H NMR spectrum of compound **2** in DMSO-d_6.

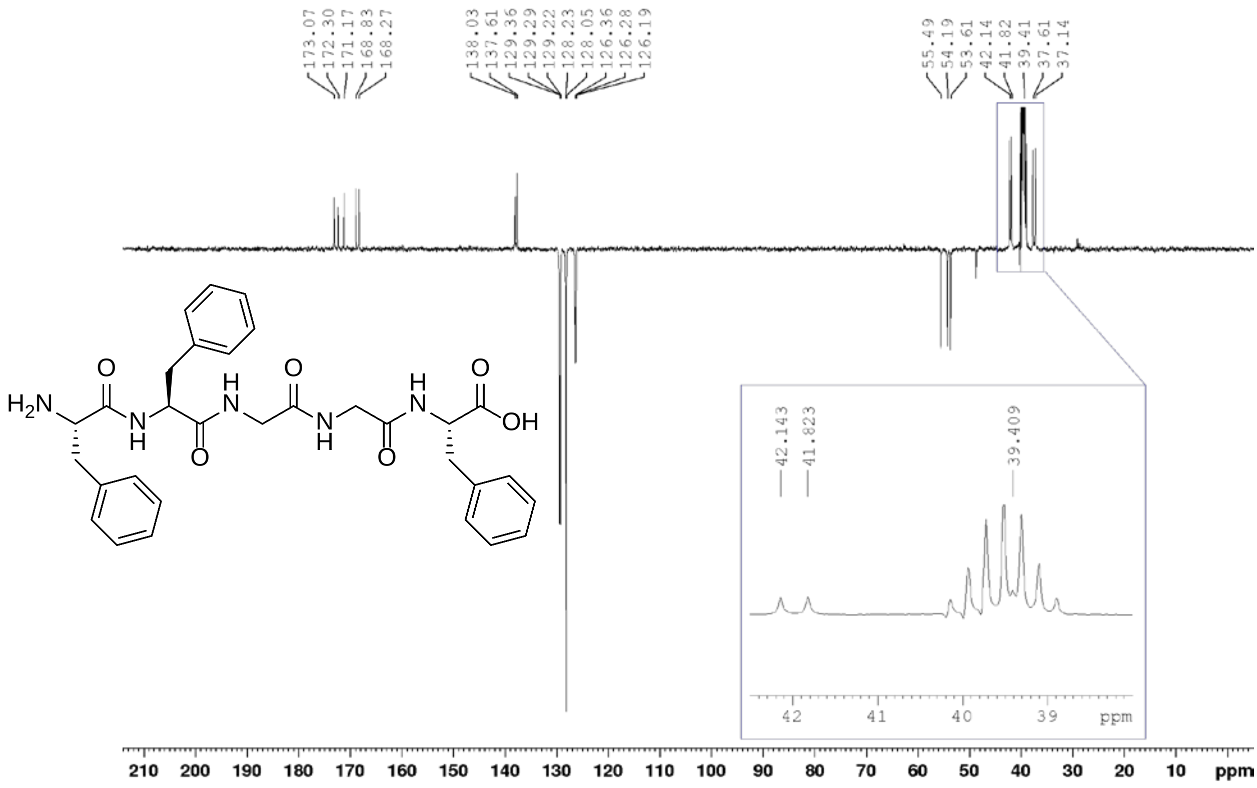

Figure S11. ^{13}C NMR spectrum of compound **2** in DMSO-d_6.

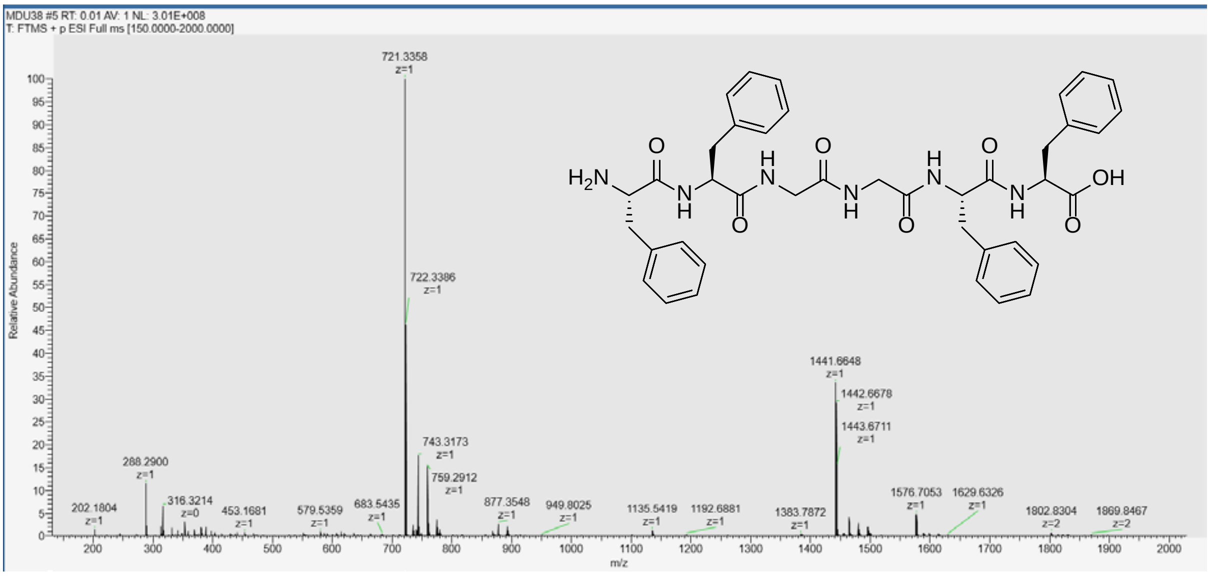

Figure S12. HRMS spectrum of compound **3**.

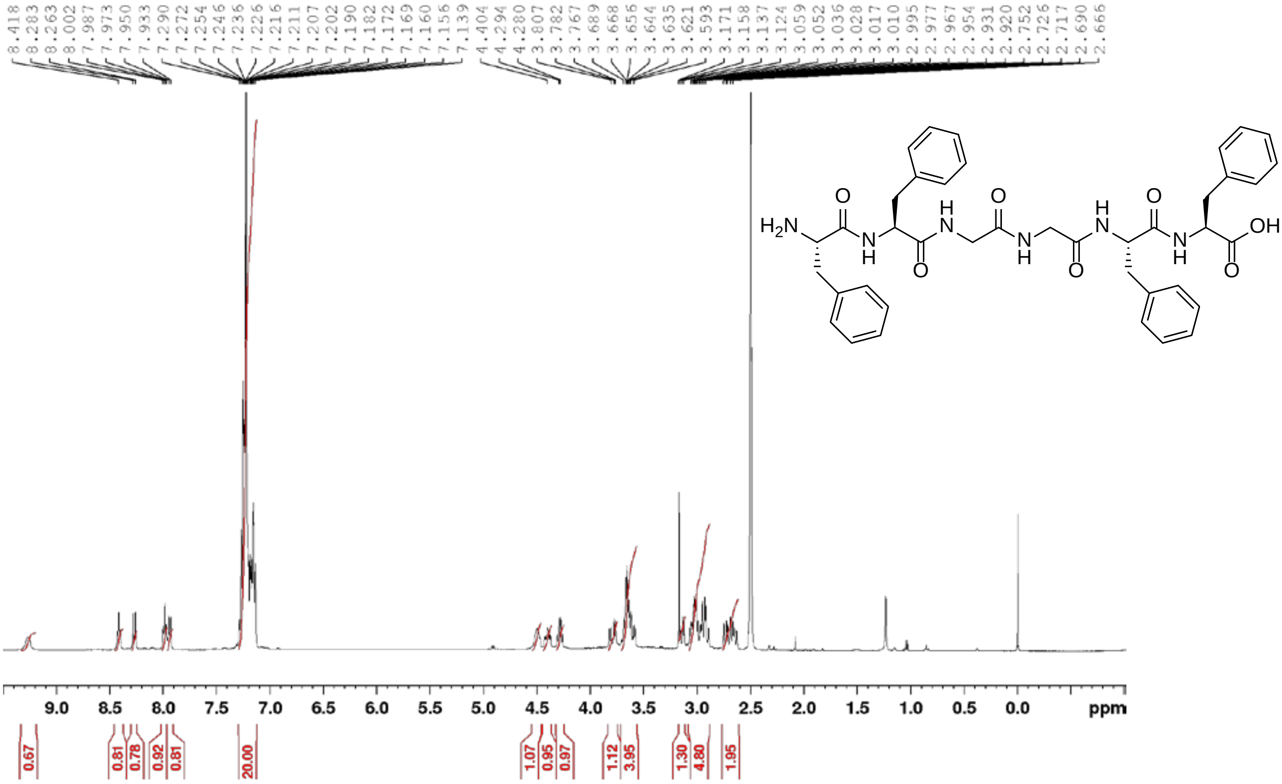

Figure S13. ^{1}H NMR spectrum of compound **3** in DMSO-d_6.

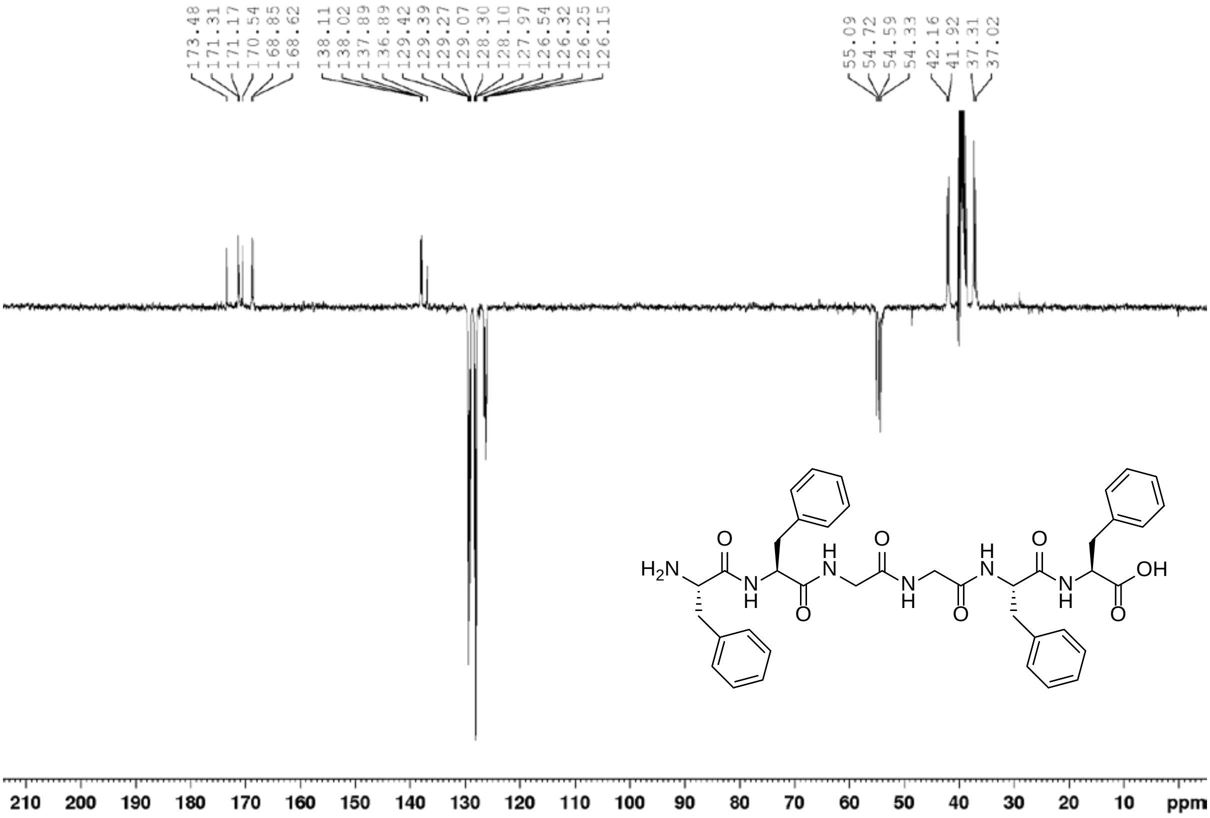

Figure S14. ^{13}C NMR spectrum of compound **3** in DMSO-d_6.

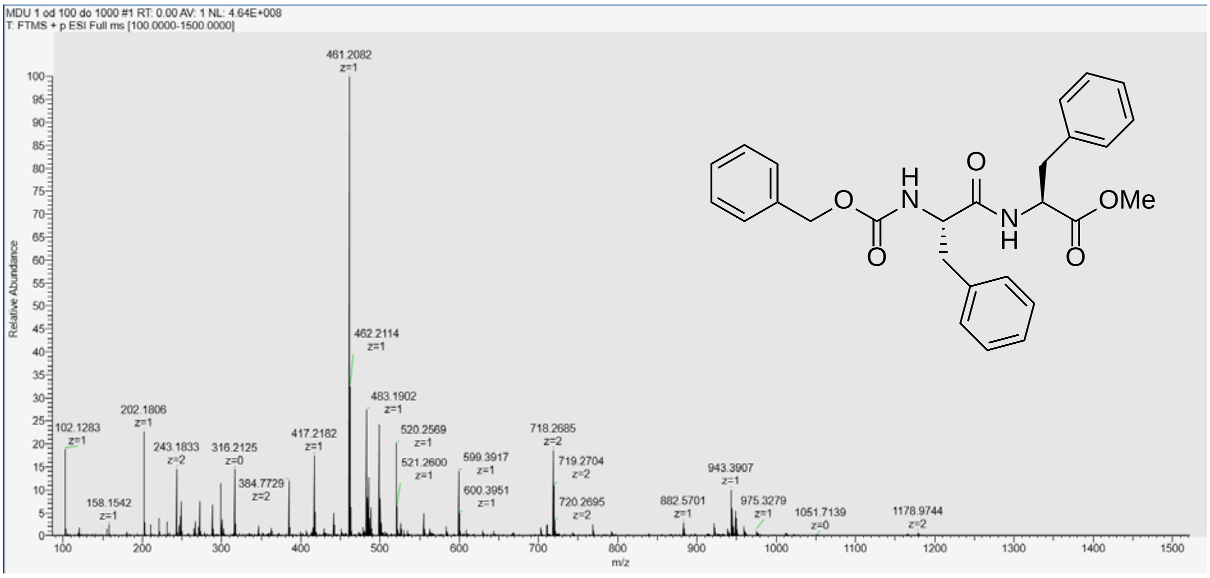

Figure S15. HRMS spectrum of compound **4**.

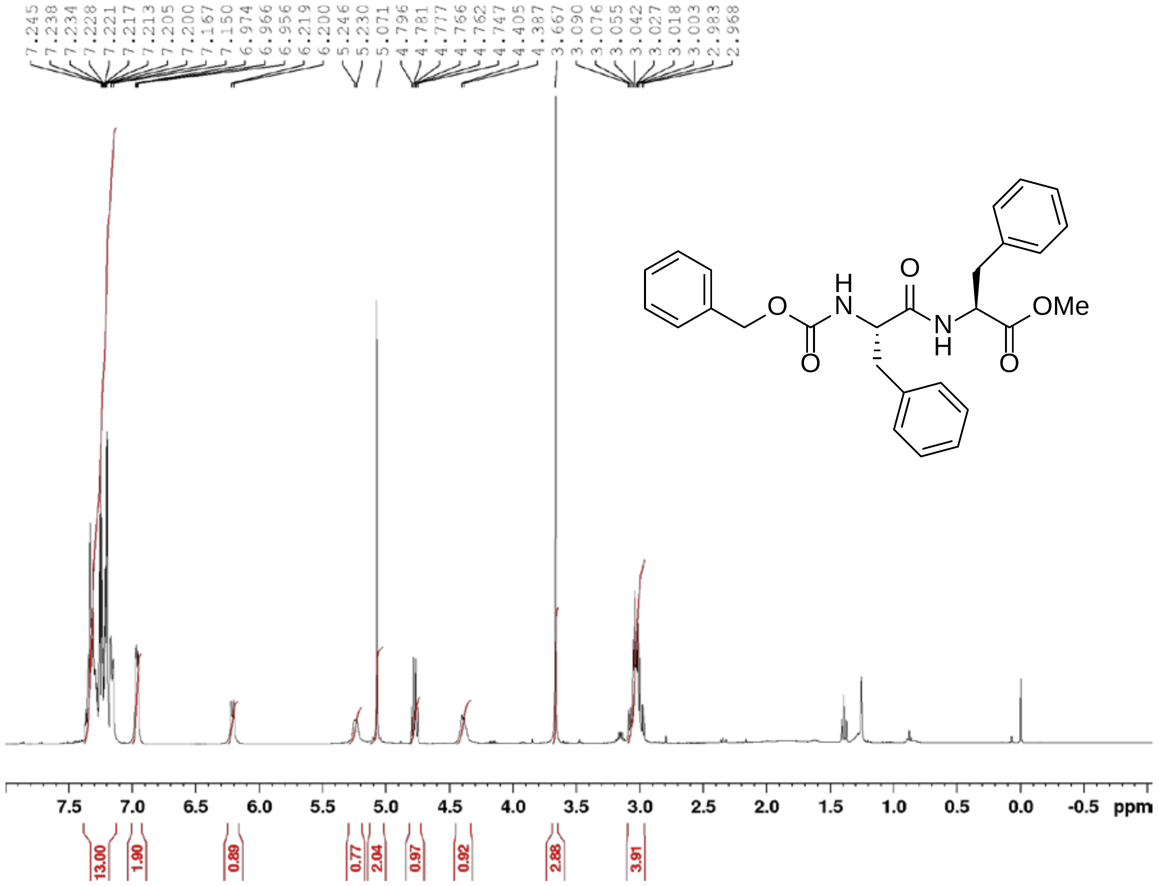

Figure S16. ^{1}H NMR spectrum of compound **4** in $CDCl_3$.

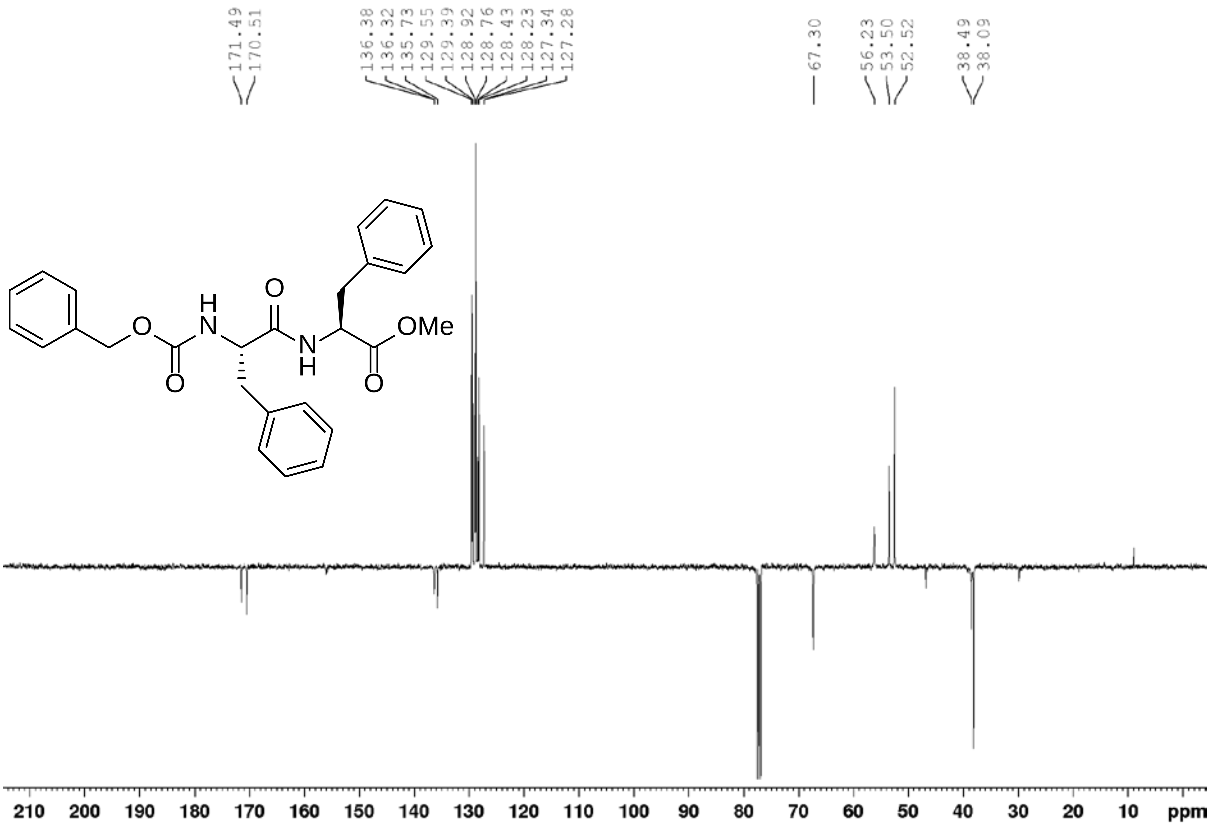

Figure S17. ^{13}C NMR spectrum of compound **4** in $CDCl_3$.

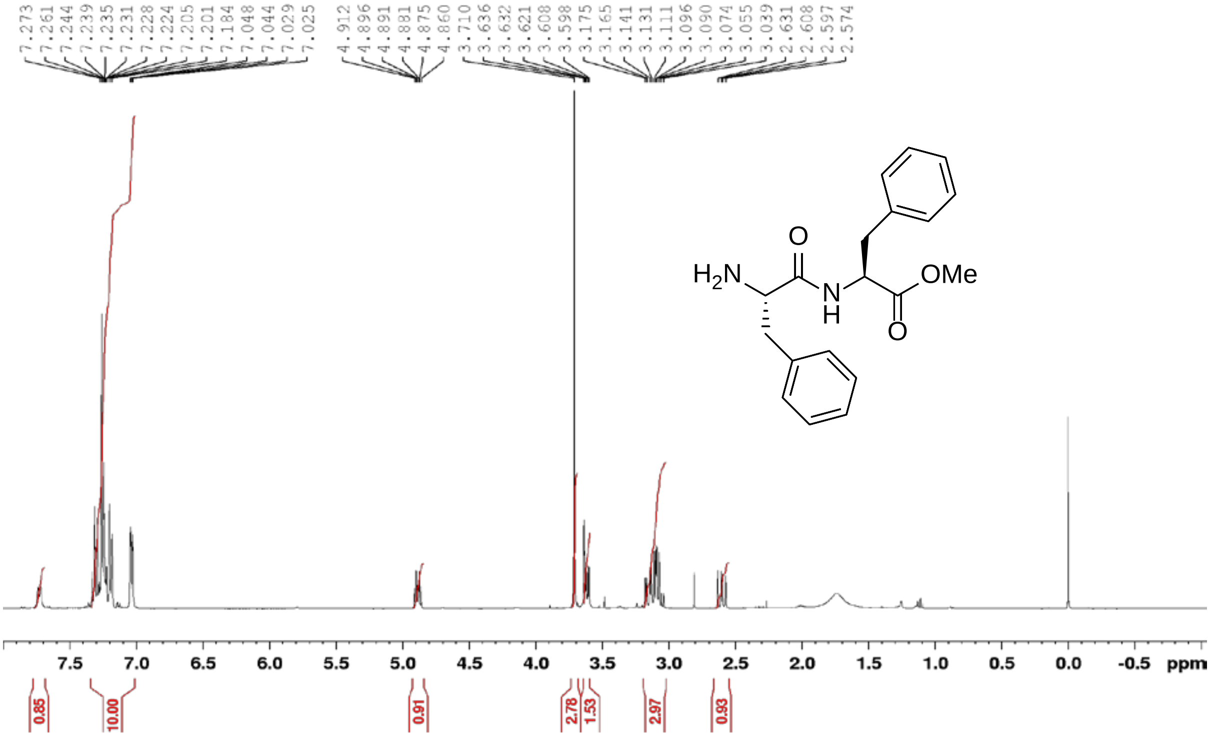

Figure S18. ^{1}H NMR spectrum of compound **5** in $CDCl_3$.

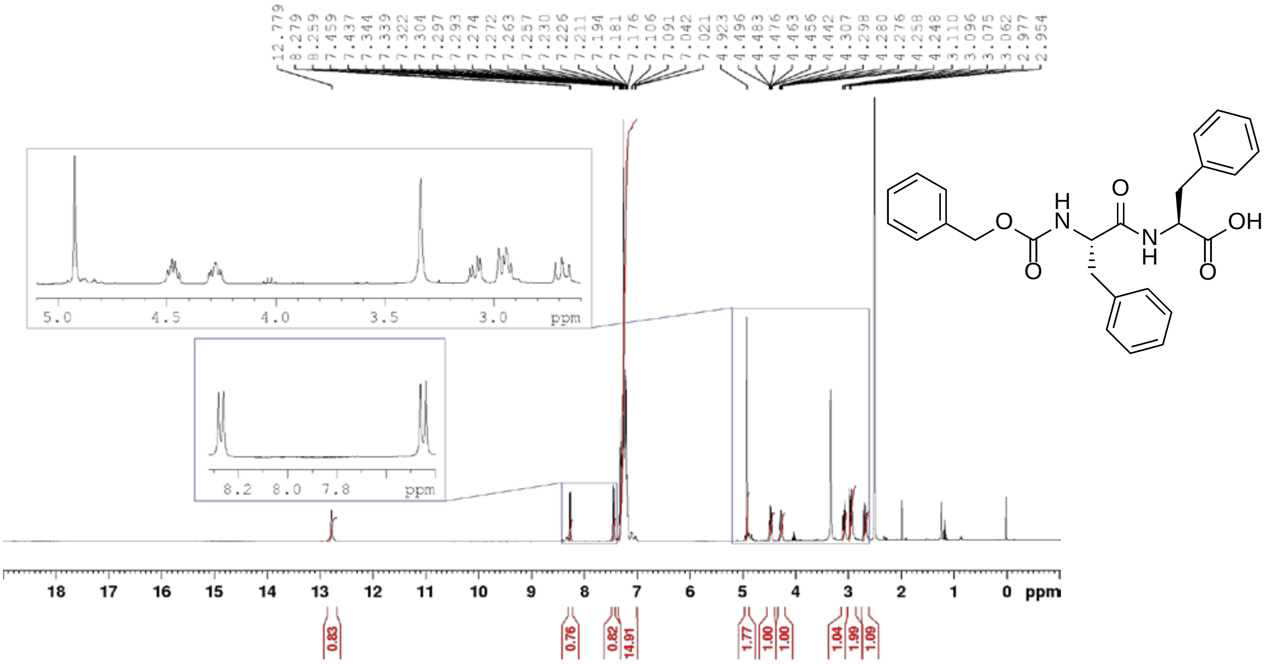

Figure S19. ^{1}H NMR spectrum of compound **6** in DMSO-d_6.

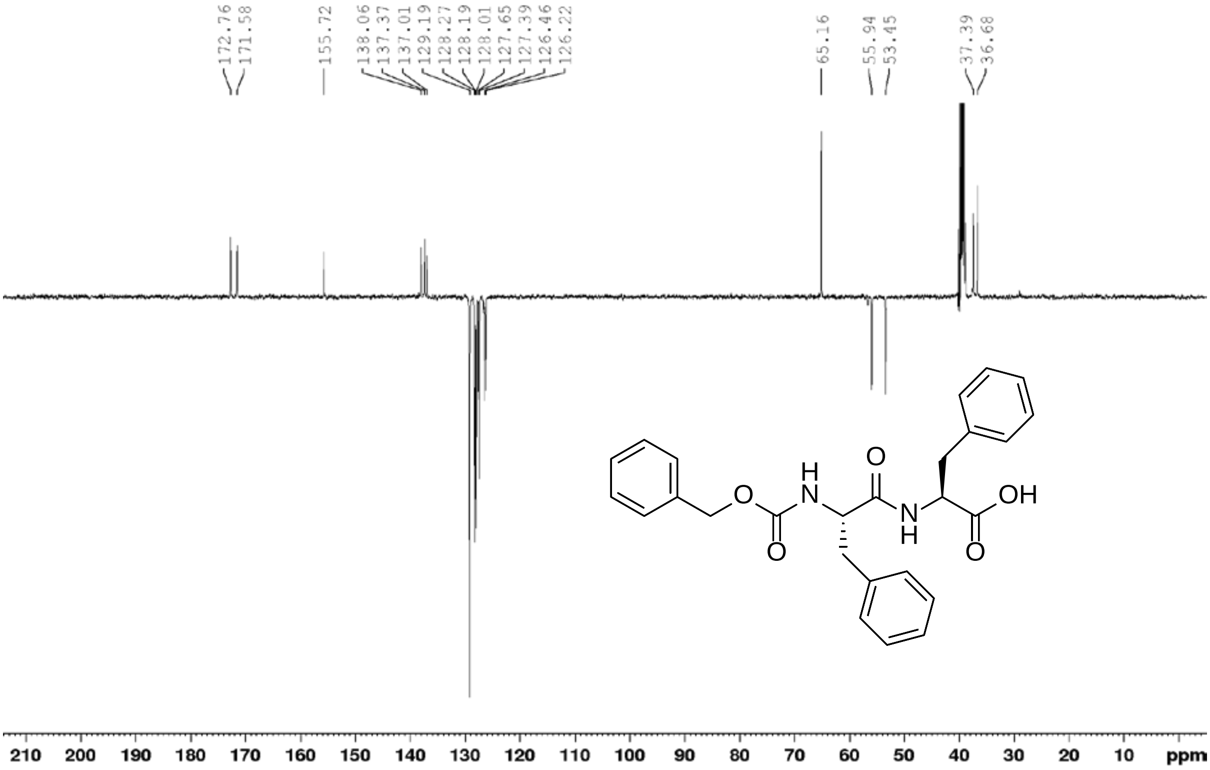

Figure S20. ^{13}C NMR spectrum of compound **6** in DMSO-d_6.

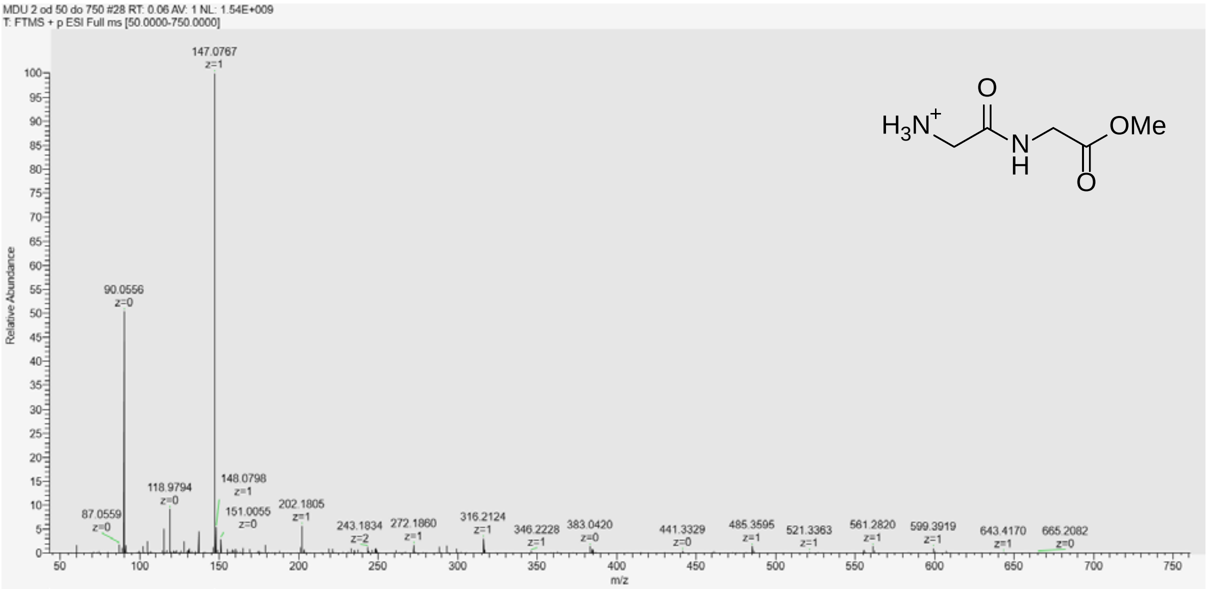

Figure S21. HRMS spectrum of compound **7**.

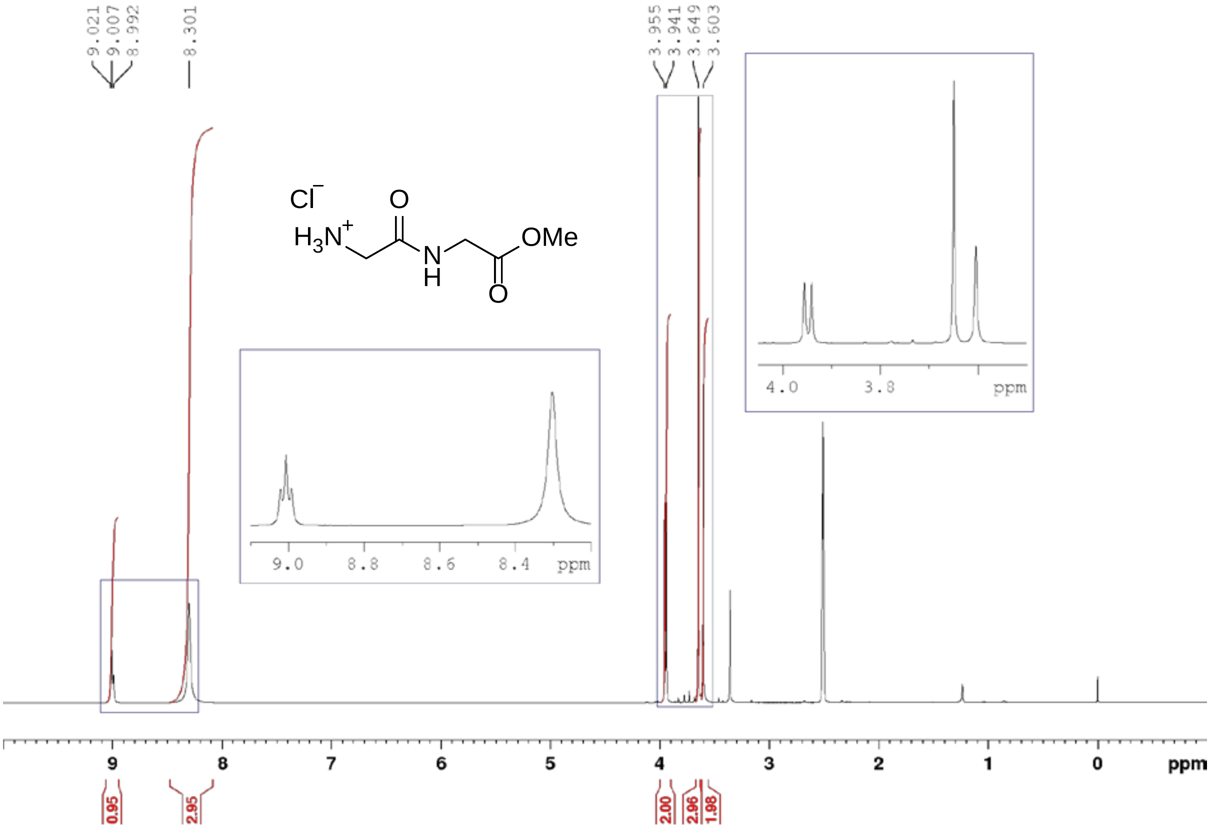

Figure S22. ^{1}H NMR spectrum of compound **7** in DMSO-d_6.

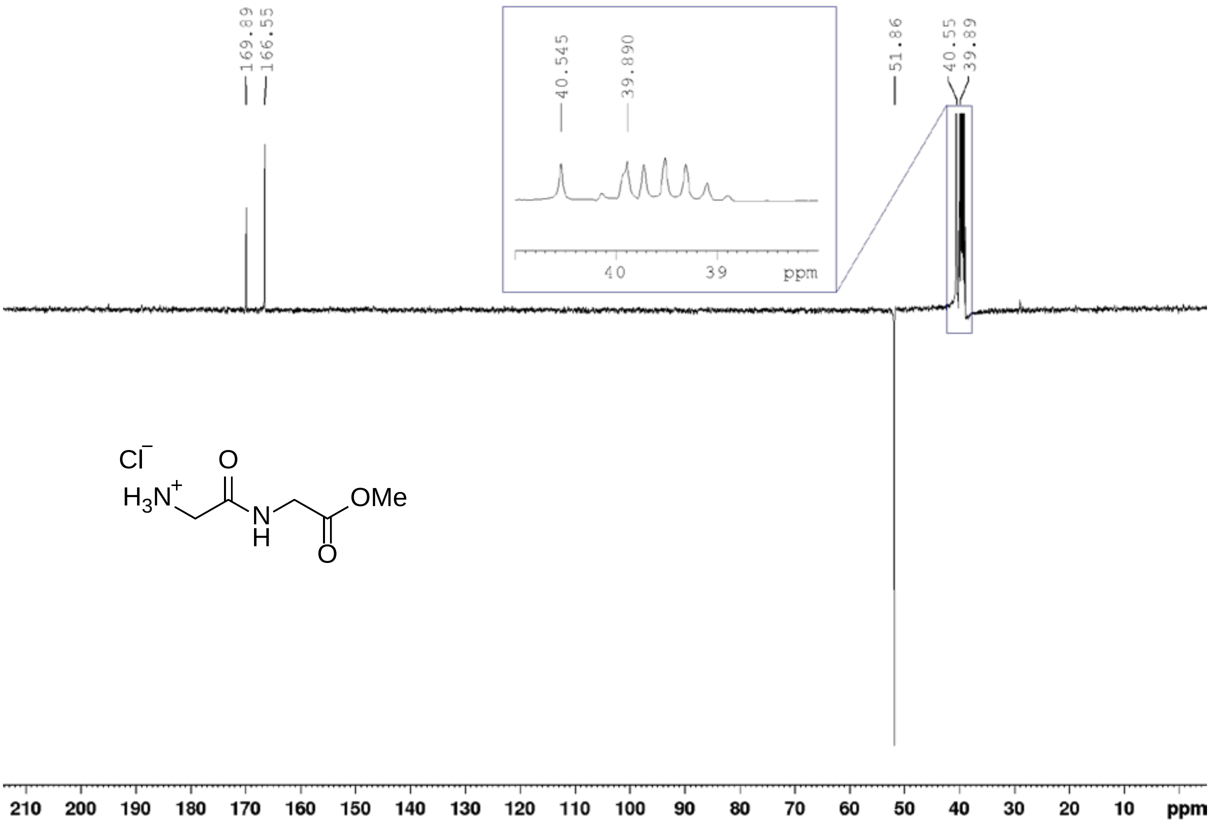

Figure S23. ^{13}C NMR spectrum of compound **7** in DMSO-d_6.

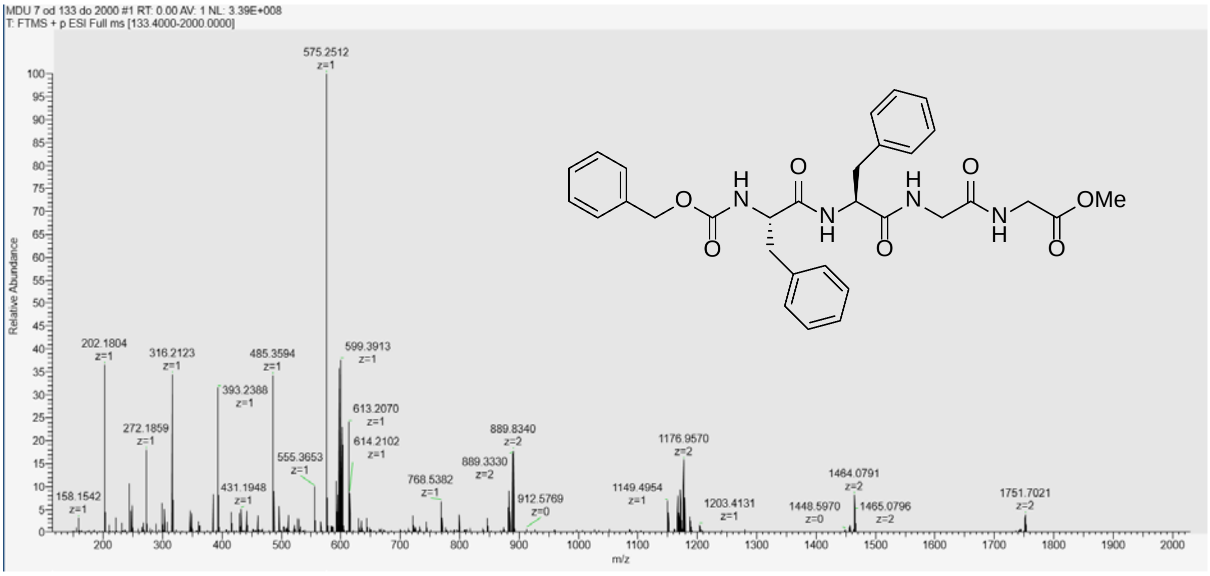

Figure S24. HRMS spectrum of compound **8**.

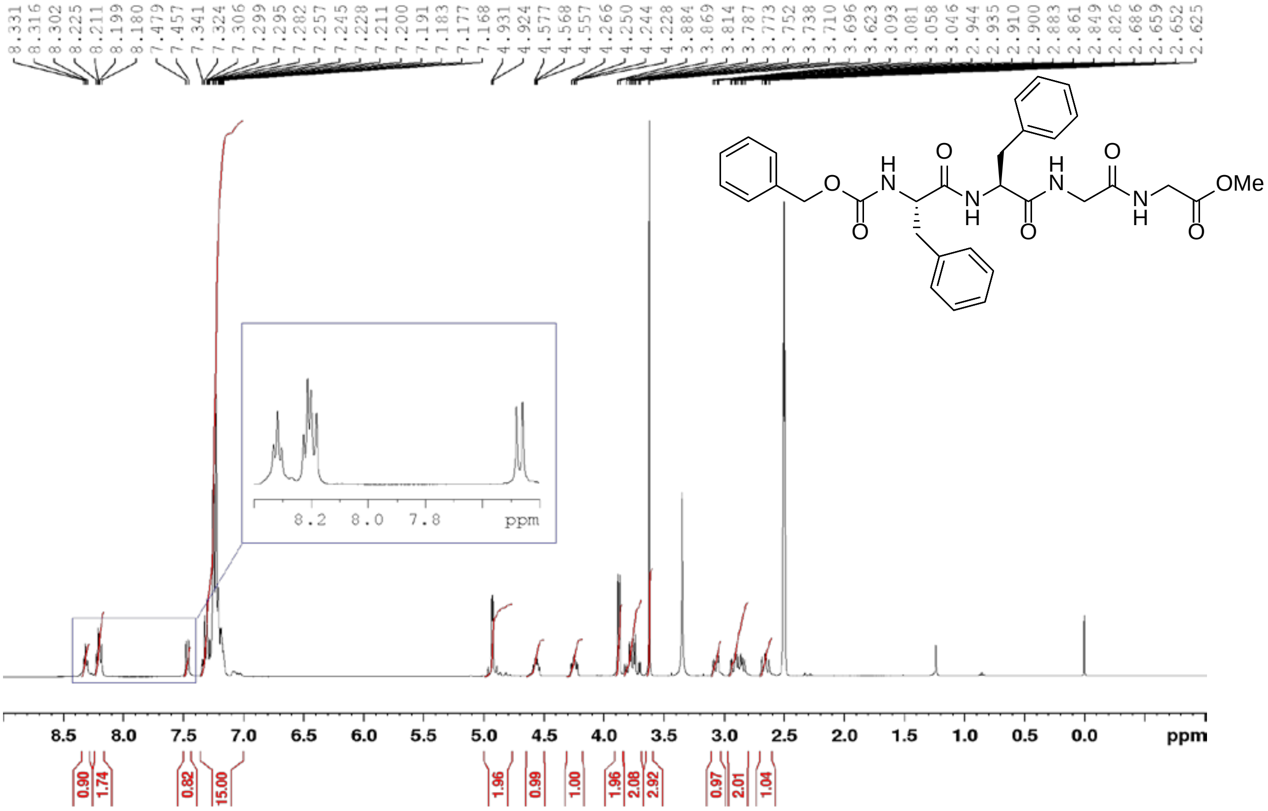

Figure S25. ^{1}H NMR spectrum of compound **8** in DMSO-d_6.

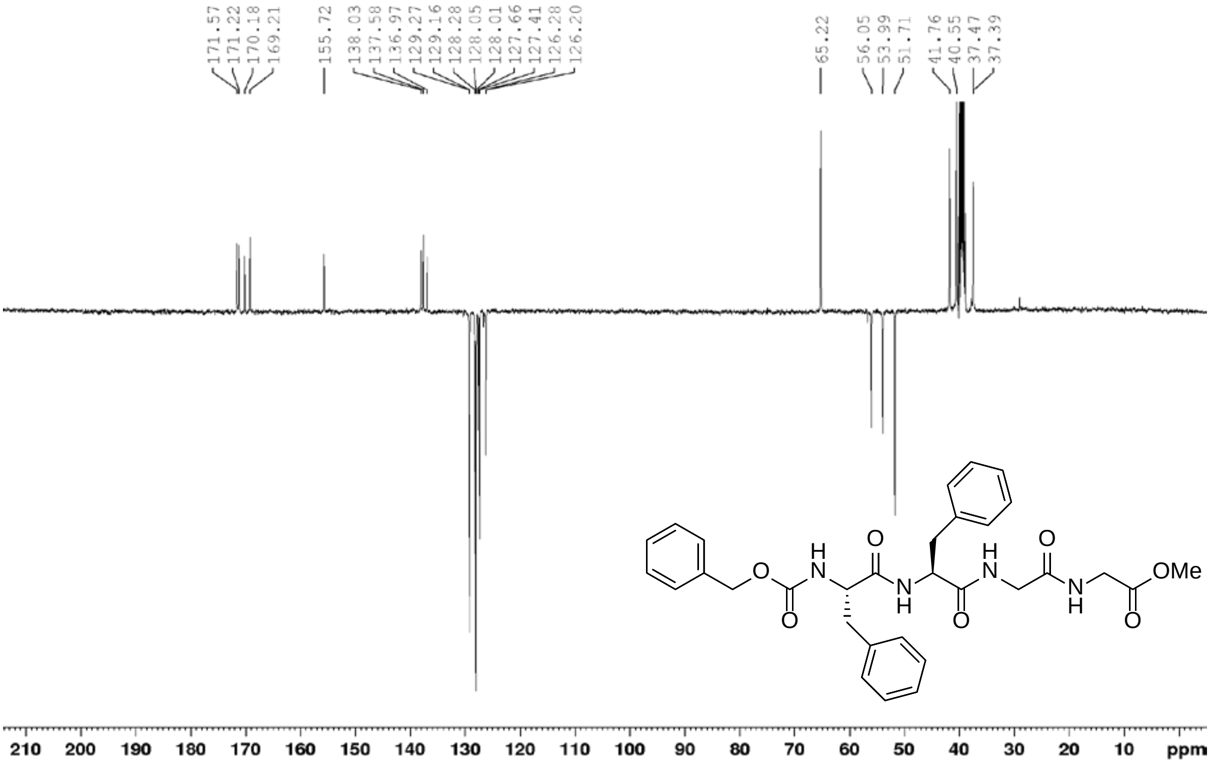

Figure S26. ^{13}C NMR spectrum of compound **8** in DMSO-d_6.

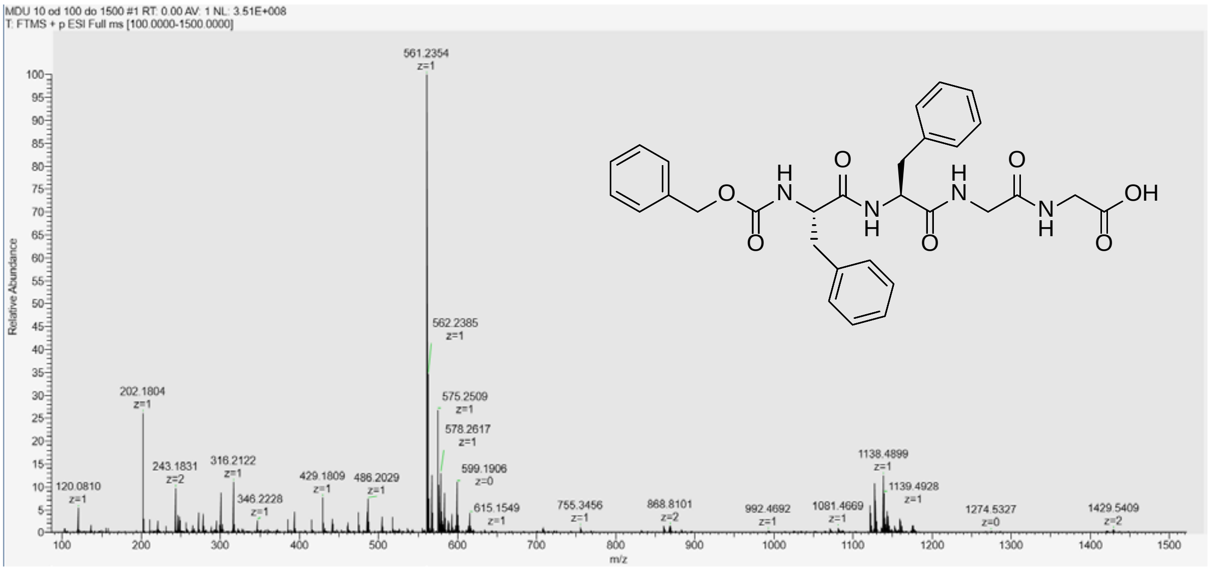

Figure S27. HRMS spectrum of compound **9**.

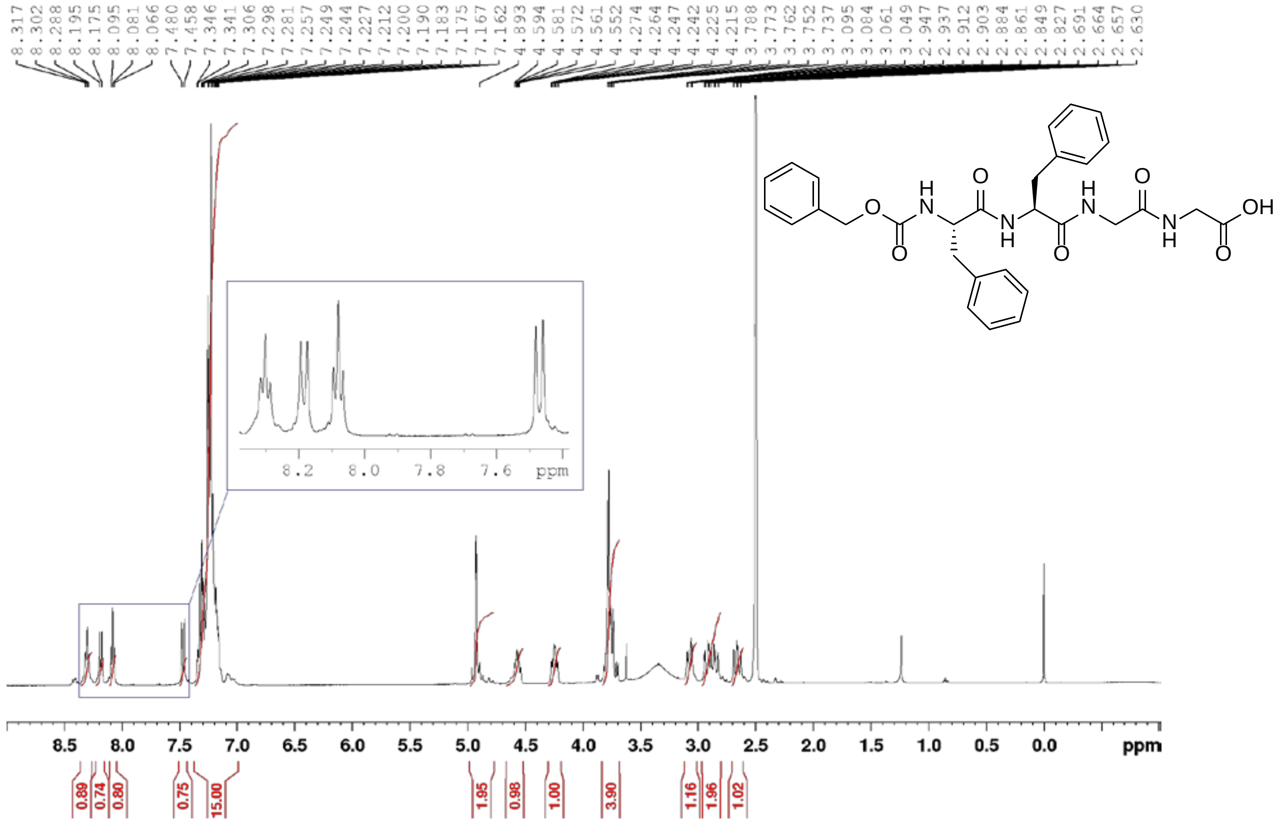

Figure S28. ^{1}H NMR spectrum of compound **9** in DMSO-d_6.

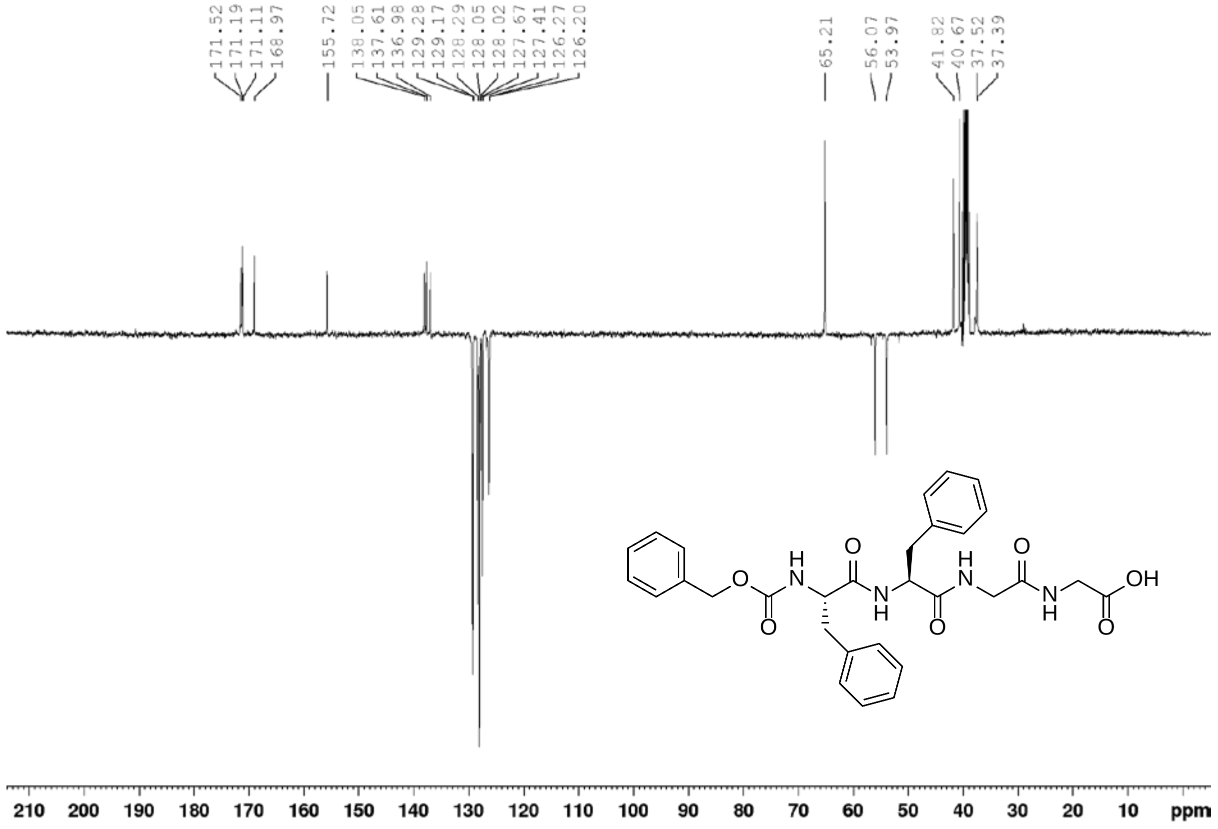

Figure S29. ^{13}C NMR spectrum of compound **9** in DMSO-d_6.

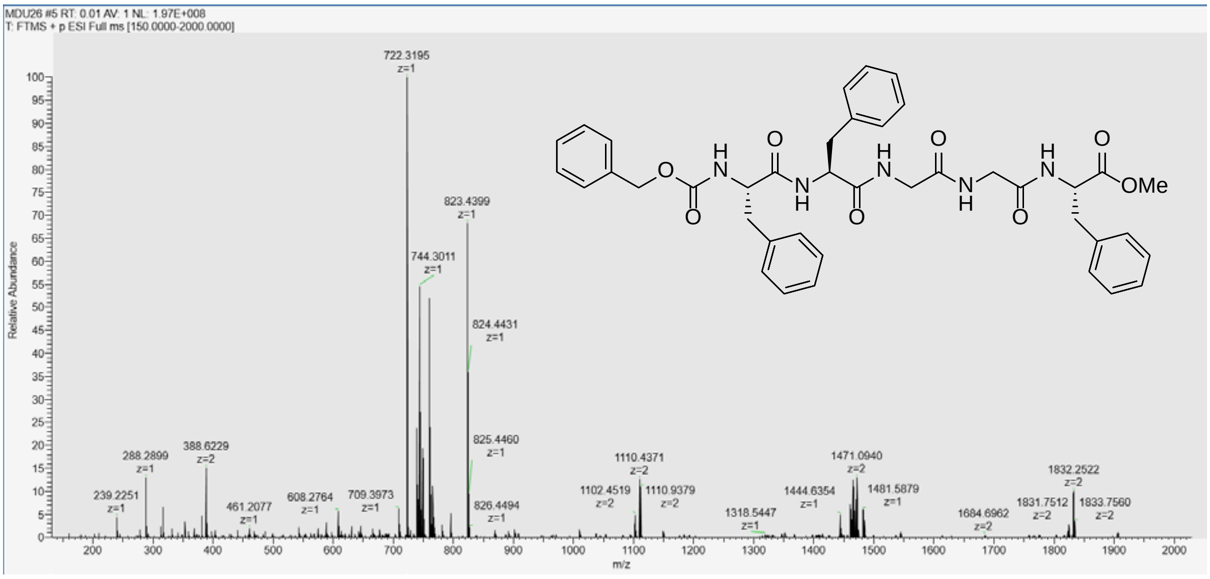

Figure S30. HRMS spectrum of compound **10**.

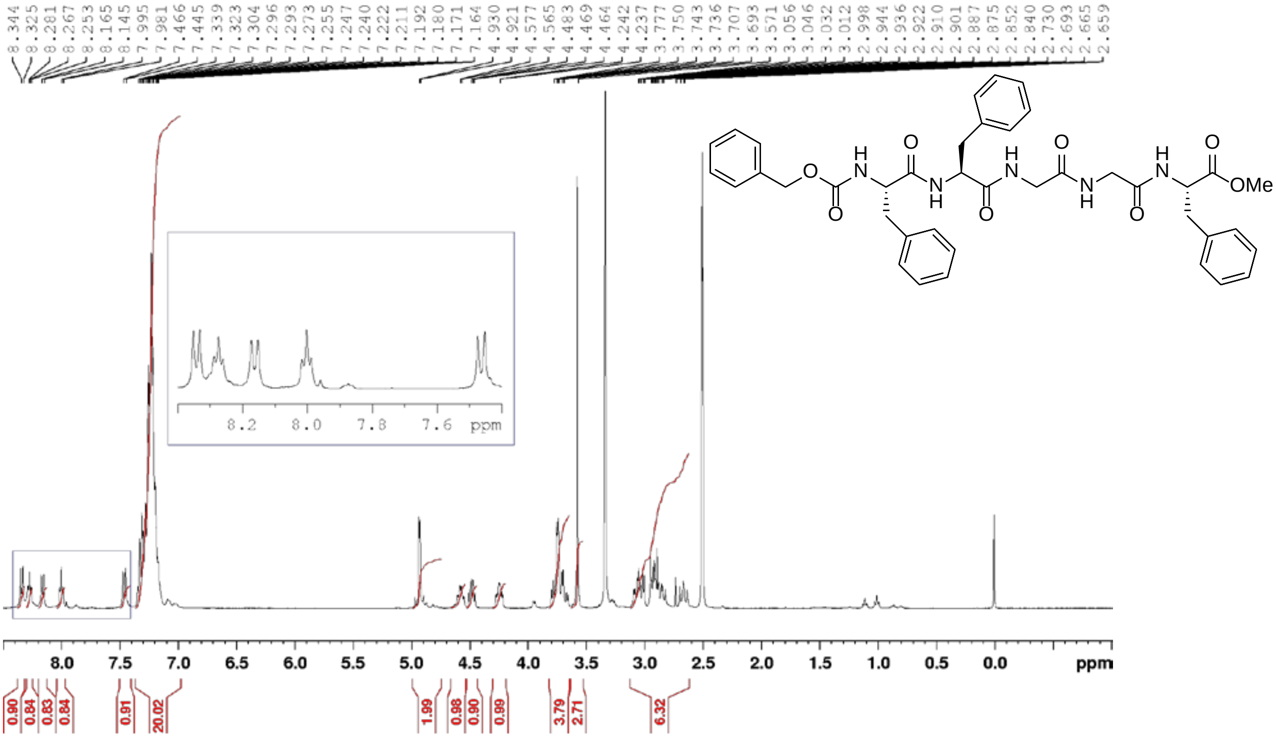

Figure S31. ^{1}H NMR spectrum of compound **10** in DMSO-d_6.

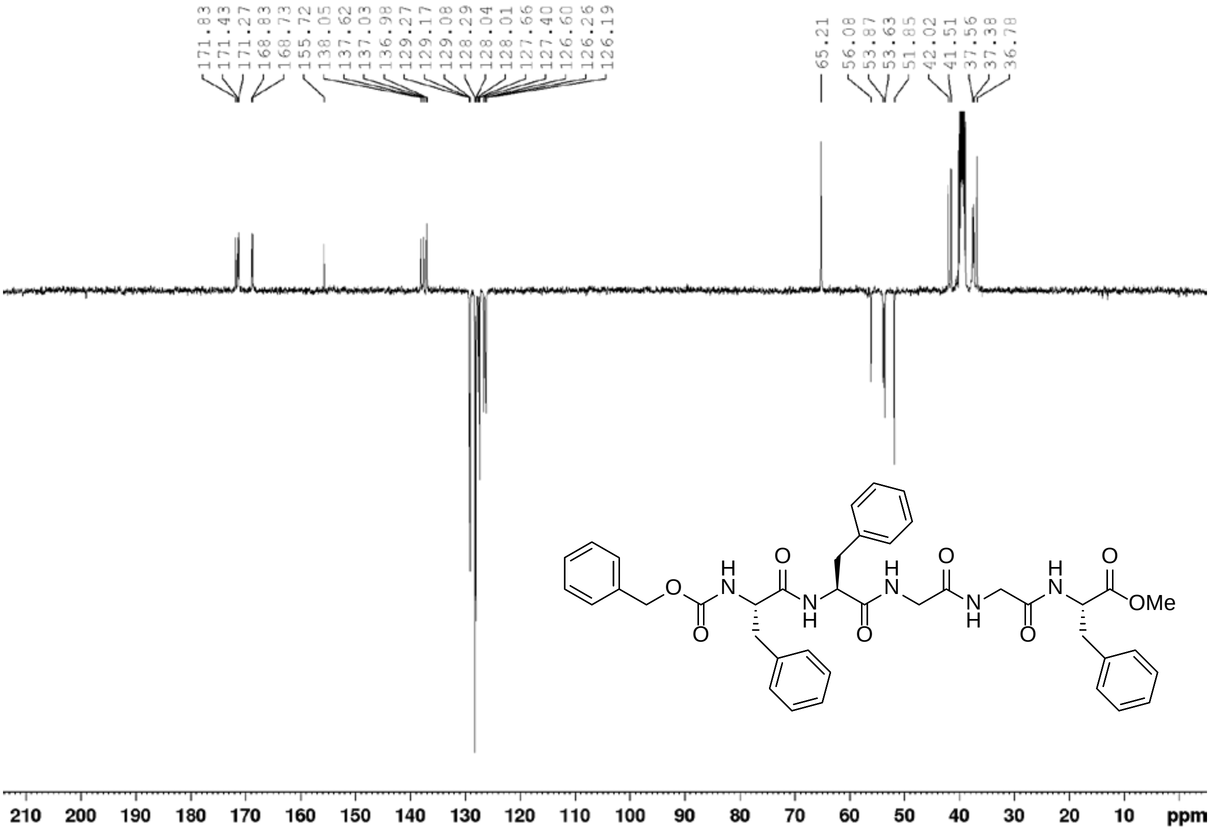

Figure S32. ^{13}C NMR spectrum of compound **10** in DMSO-d_6.

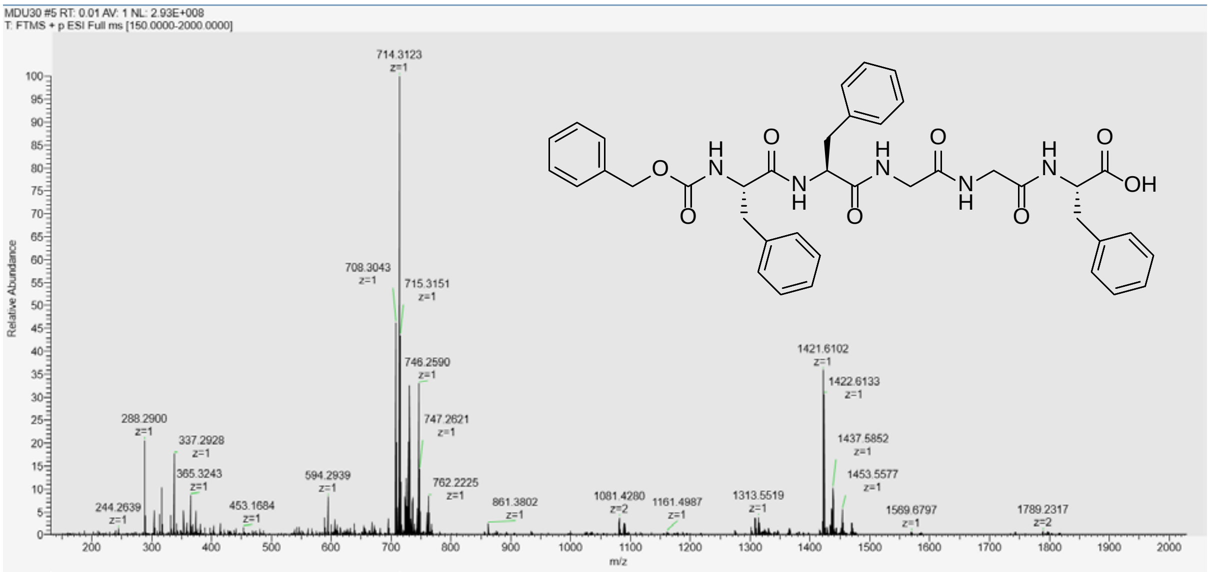

Figure S33. HRMS spectrum of compound **11**.

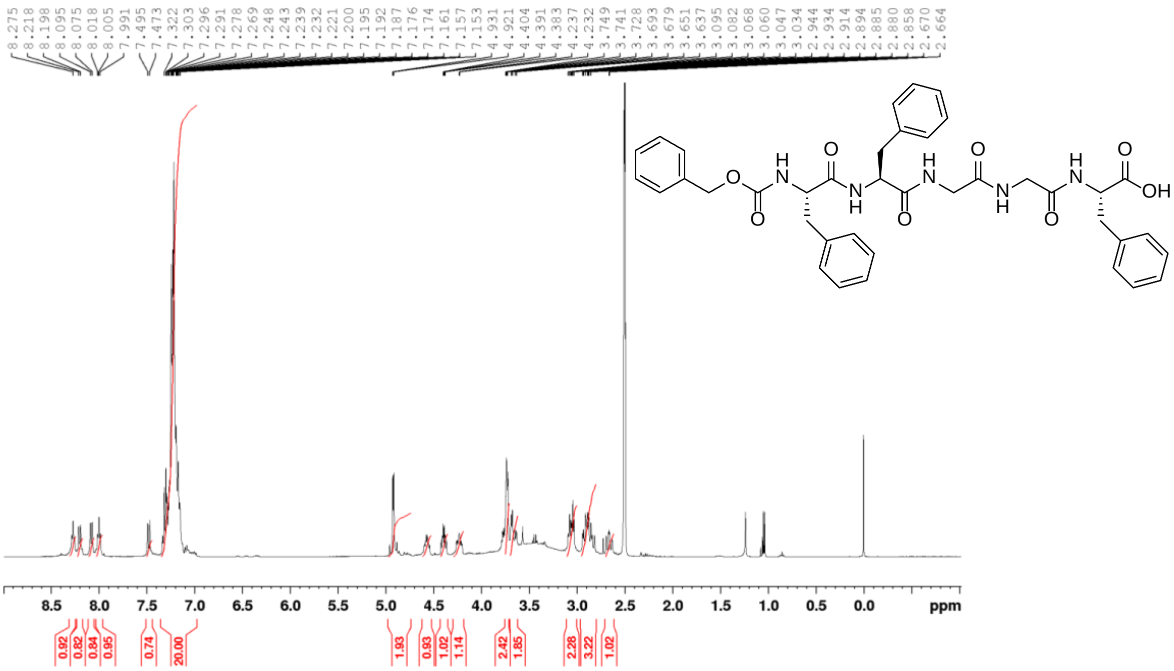

Figure S34. ^{1}H NMR spectrum of compound **11** in DMSO-d_6.

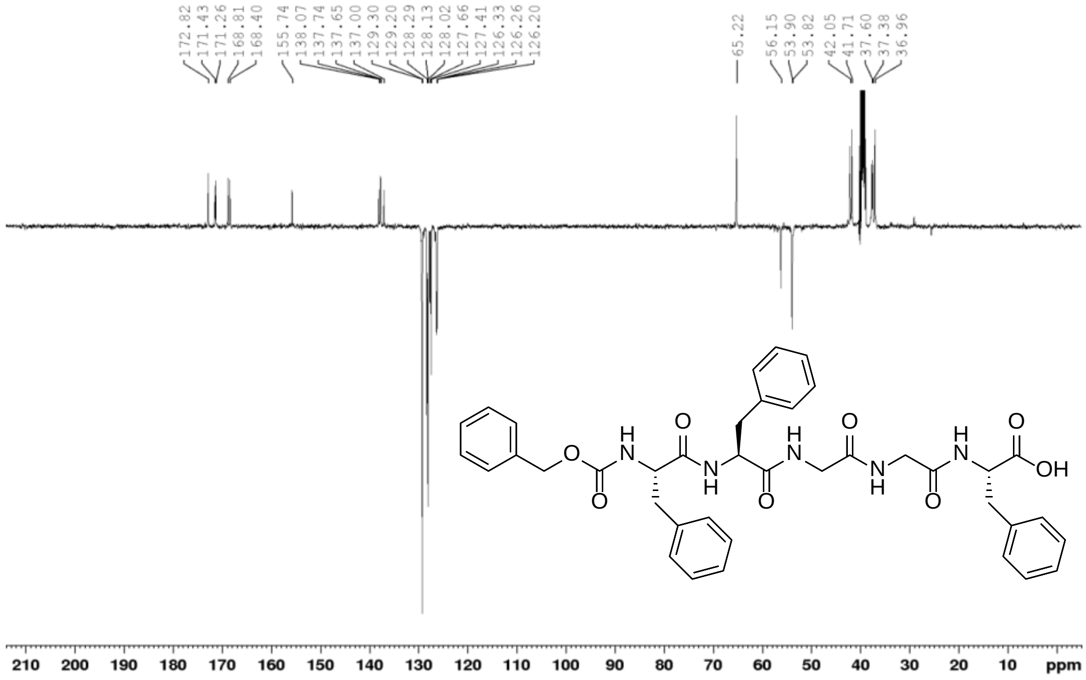

Figure S35. ^{13}C NMR spectrum of compound **11** in DMSO-d_6.

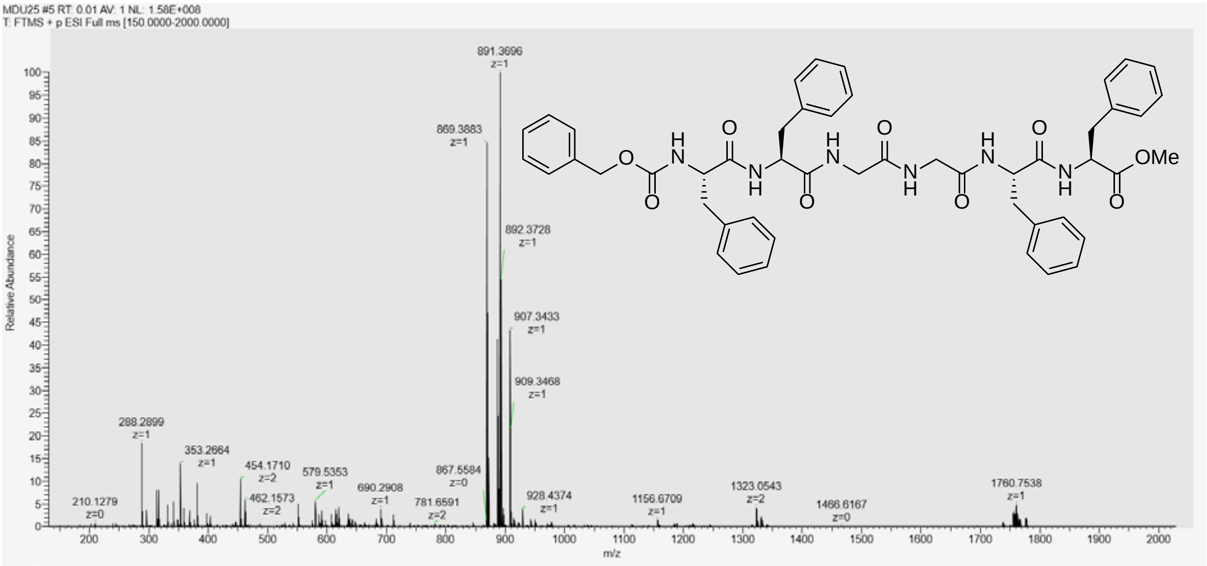

Figure S36. HRMS spectrum of compound **12**.

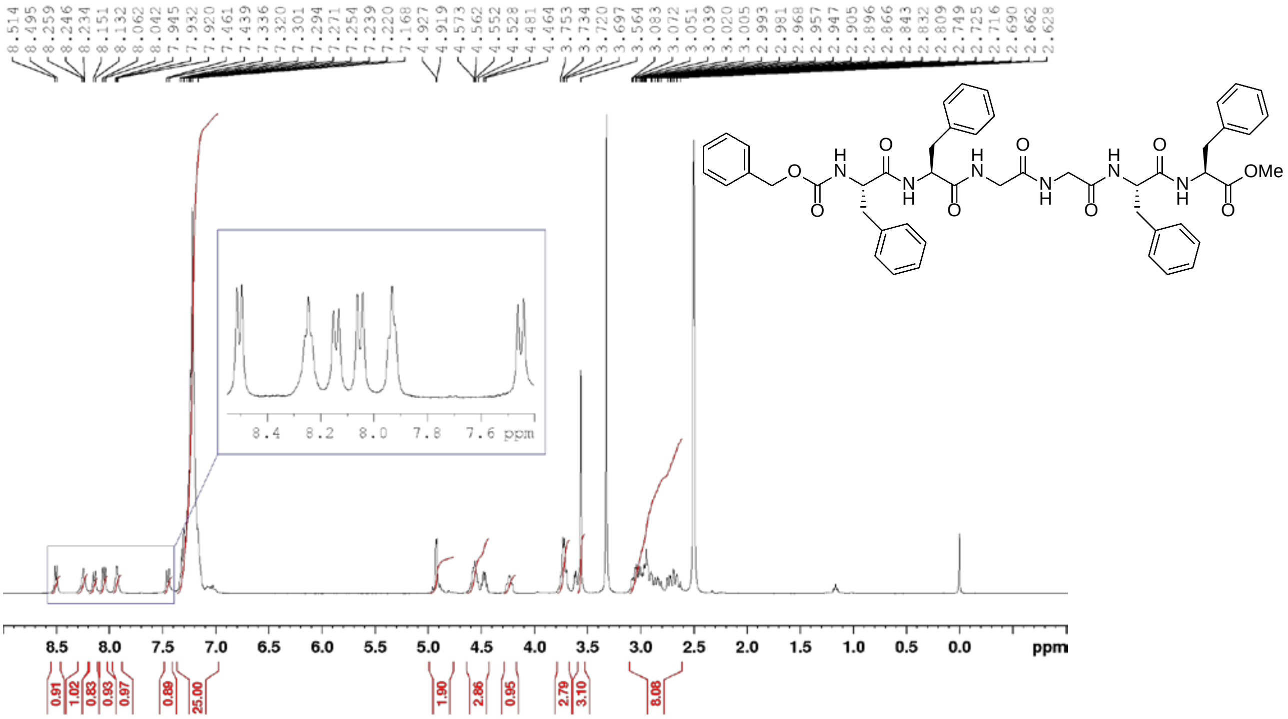

Figure S37. ^{1}H NMR spectrum of compound **12** in DMSO-d_6.

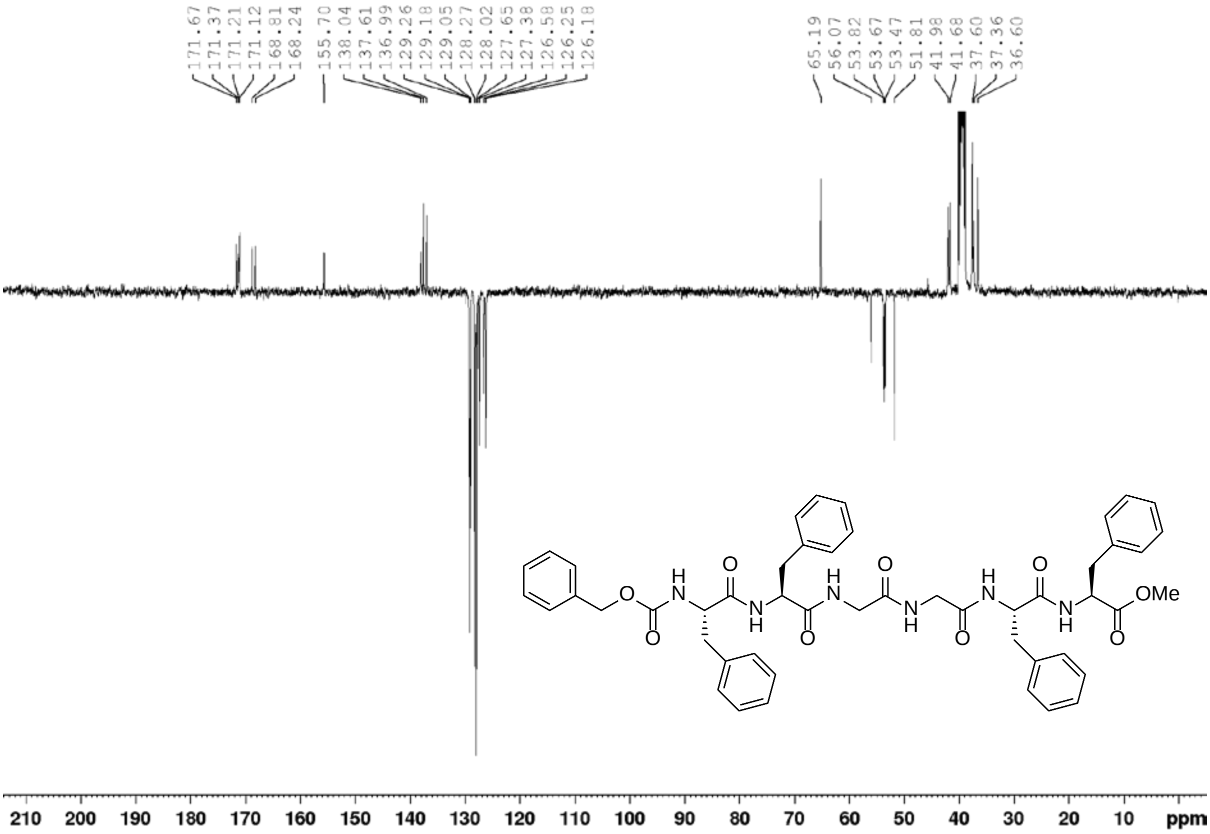

Figure S38. ^{13}C NMR spectrum of compound **12** in DMSO-d_6.

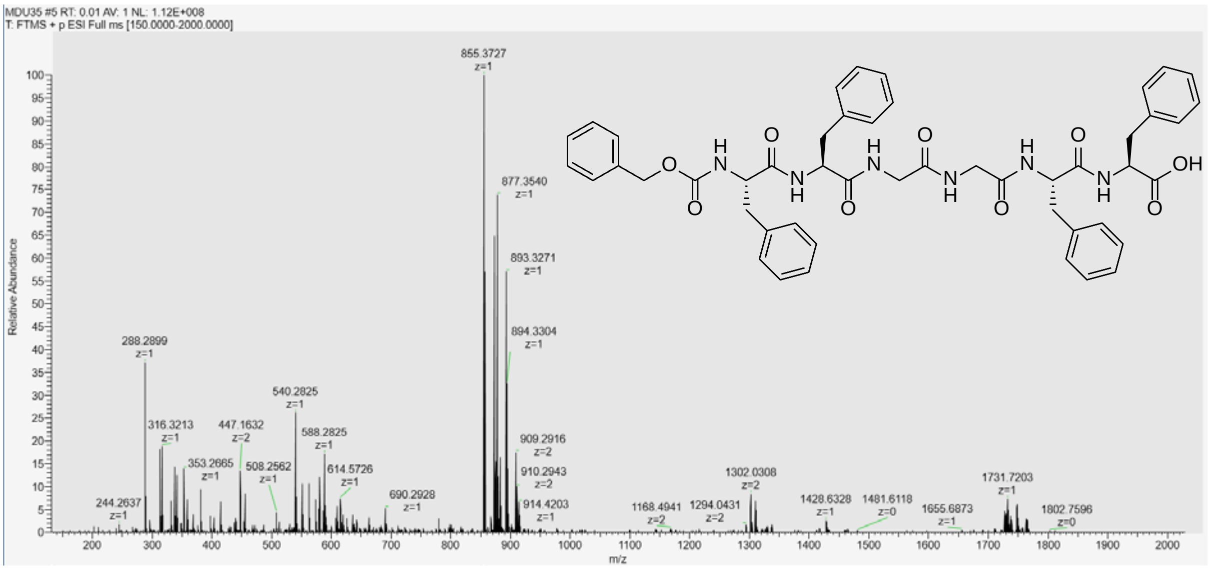

Figure S39. HRMS spectrum of compound **13**.

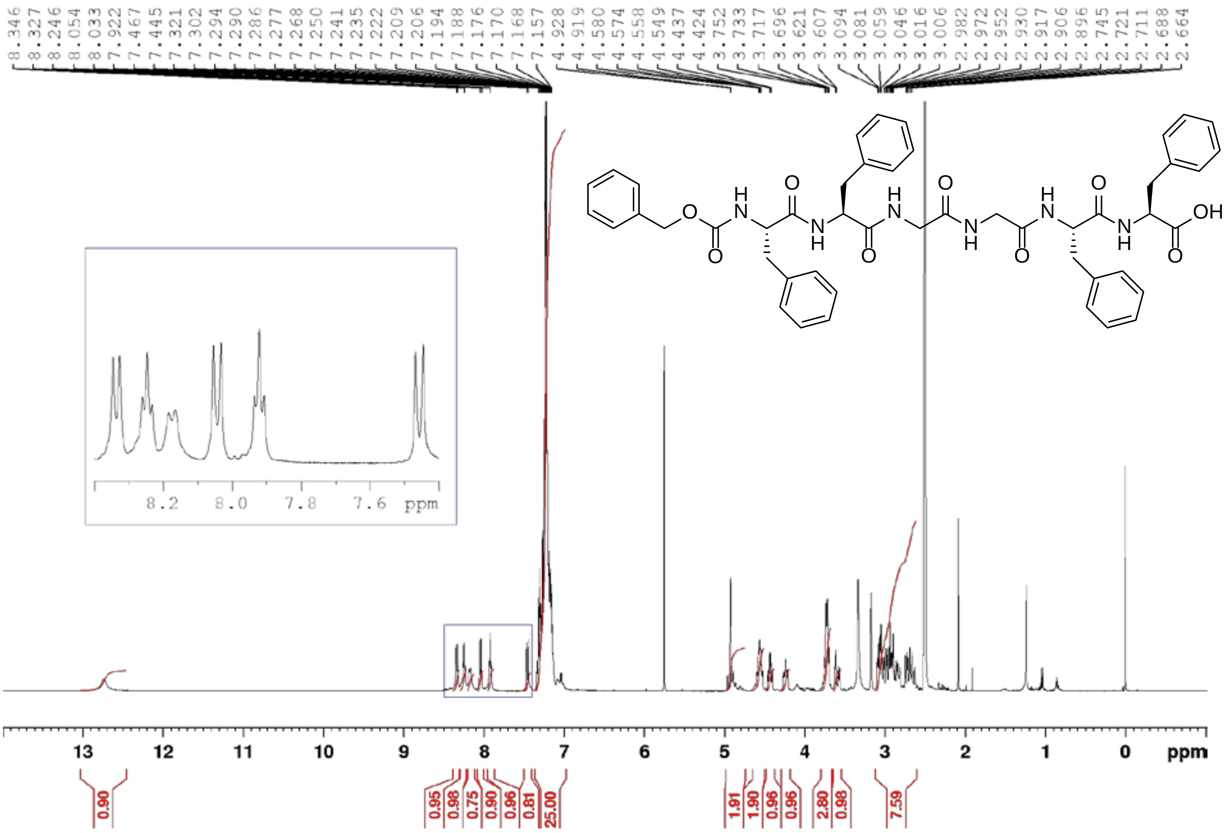

Figure S40. ^{1}H NMR spectrum of compound **13** in DMSO-d_6.

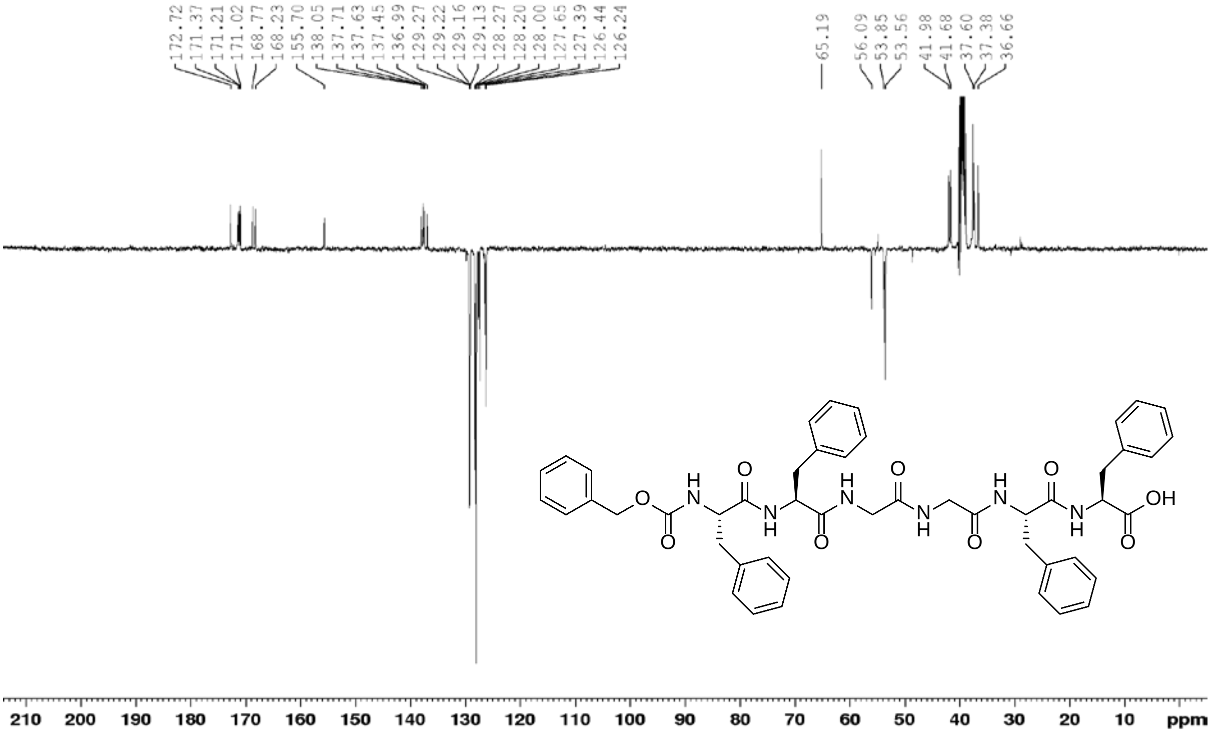

Figure S41. ^{13}C NMR spectrum of compound **13** in DMSO-d_6.

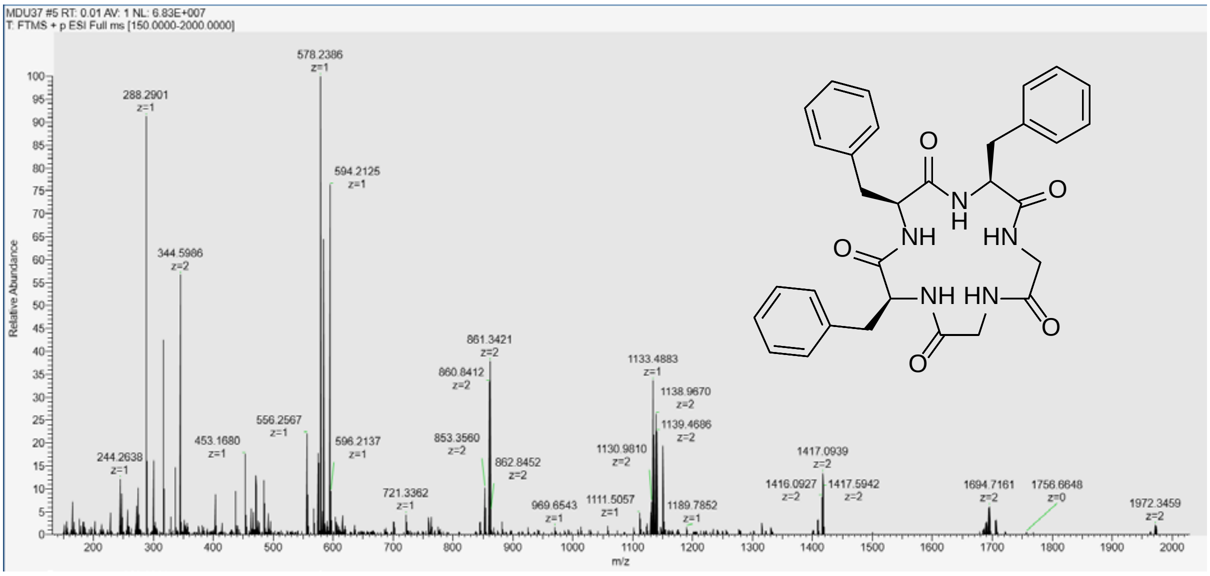

Figure S42. HRMS spectrum of compound **C2**.

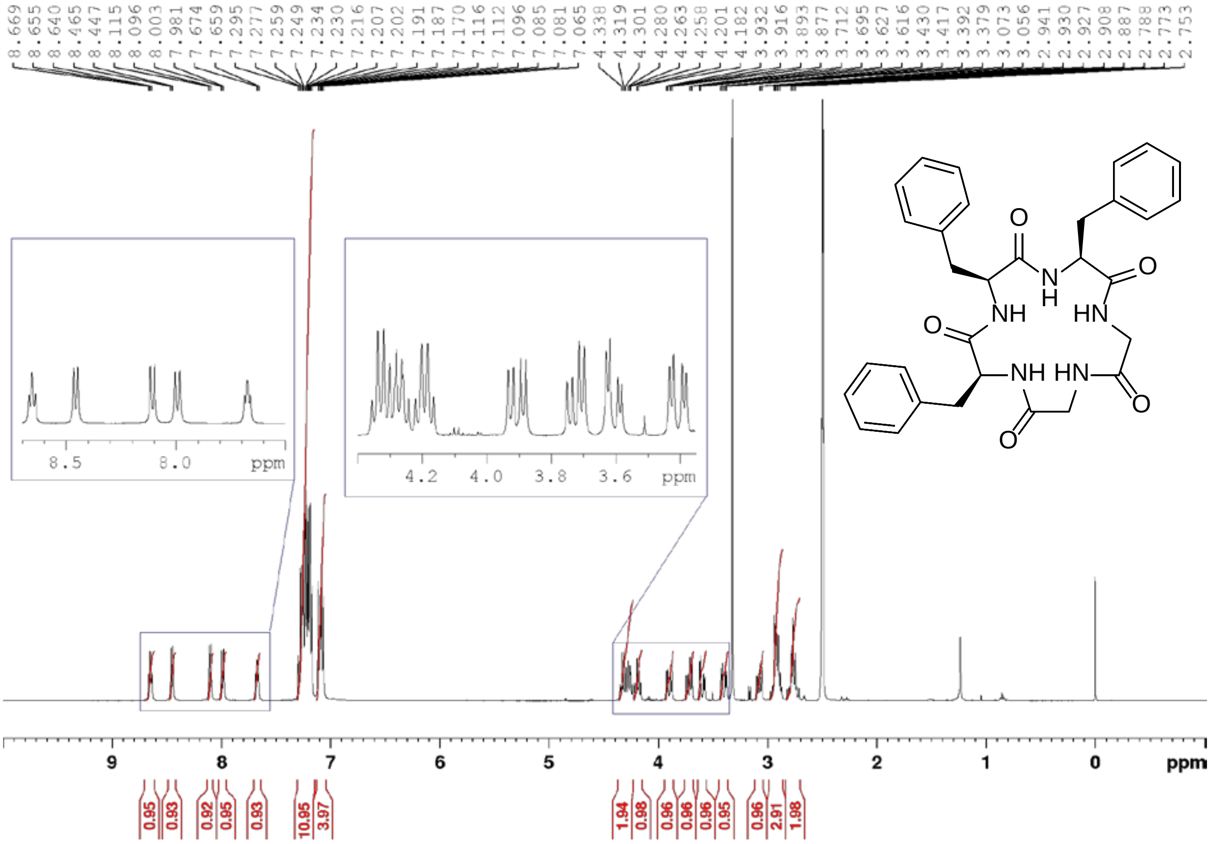

Figure S43. ^{1}H NMR spectrum of compound **C2** in DMSO-d_6.

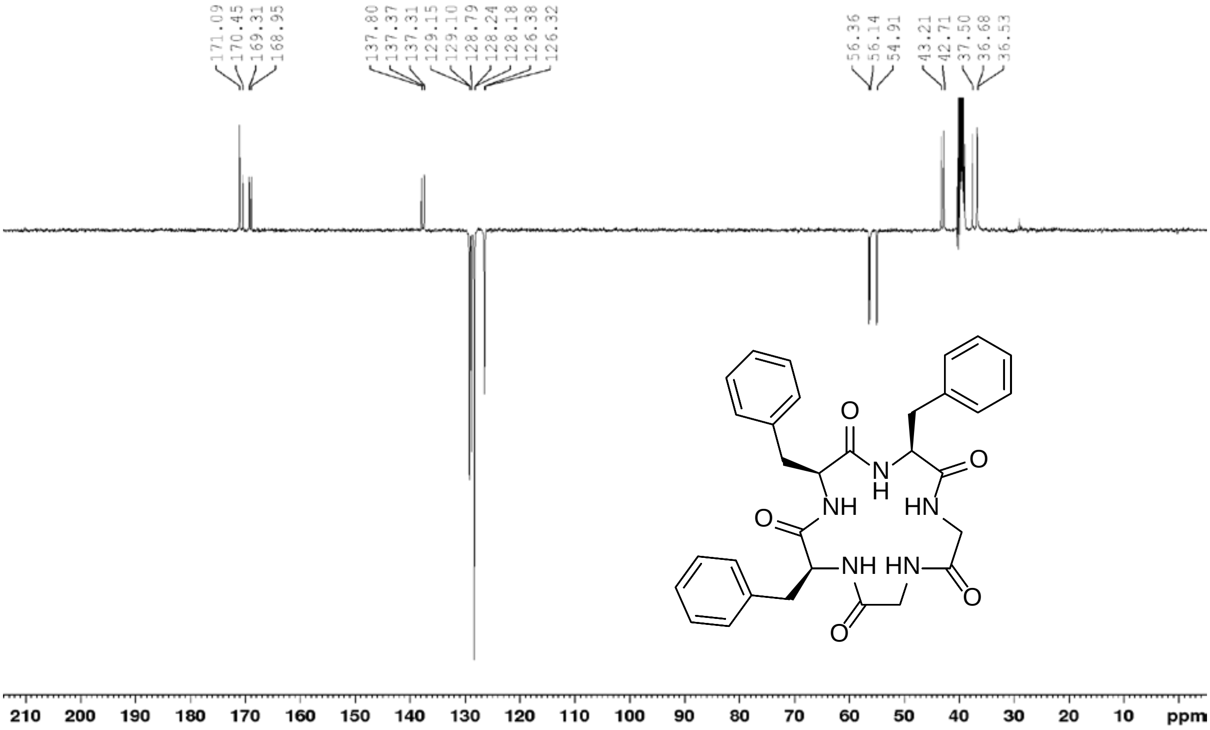

Figure S44. ^{13}C NMR spectrum of compound **C2** in DMSO-d_6.

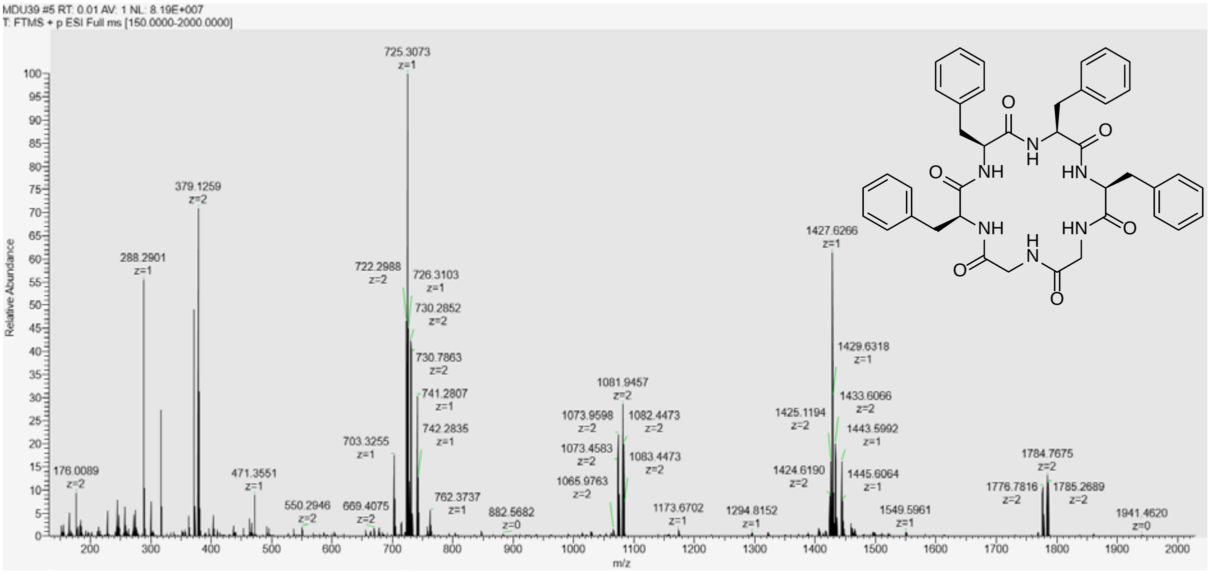

Figure S45. HRMS spectrum of compound **C3**.

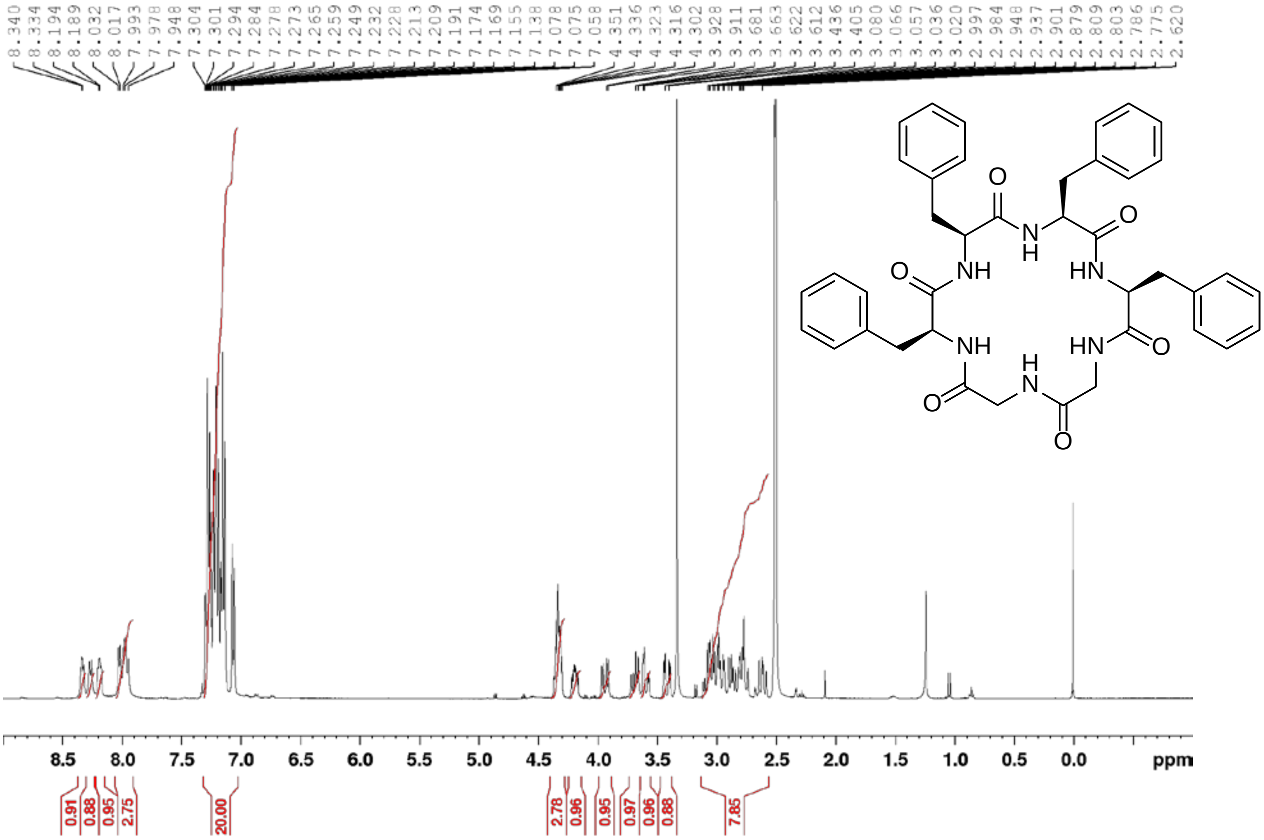

Figure S46. ^{1}H NMR spectrum of compound **C3** in DMSO-d_6.

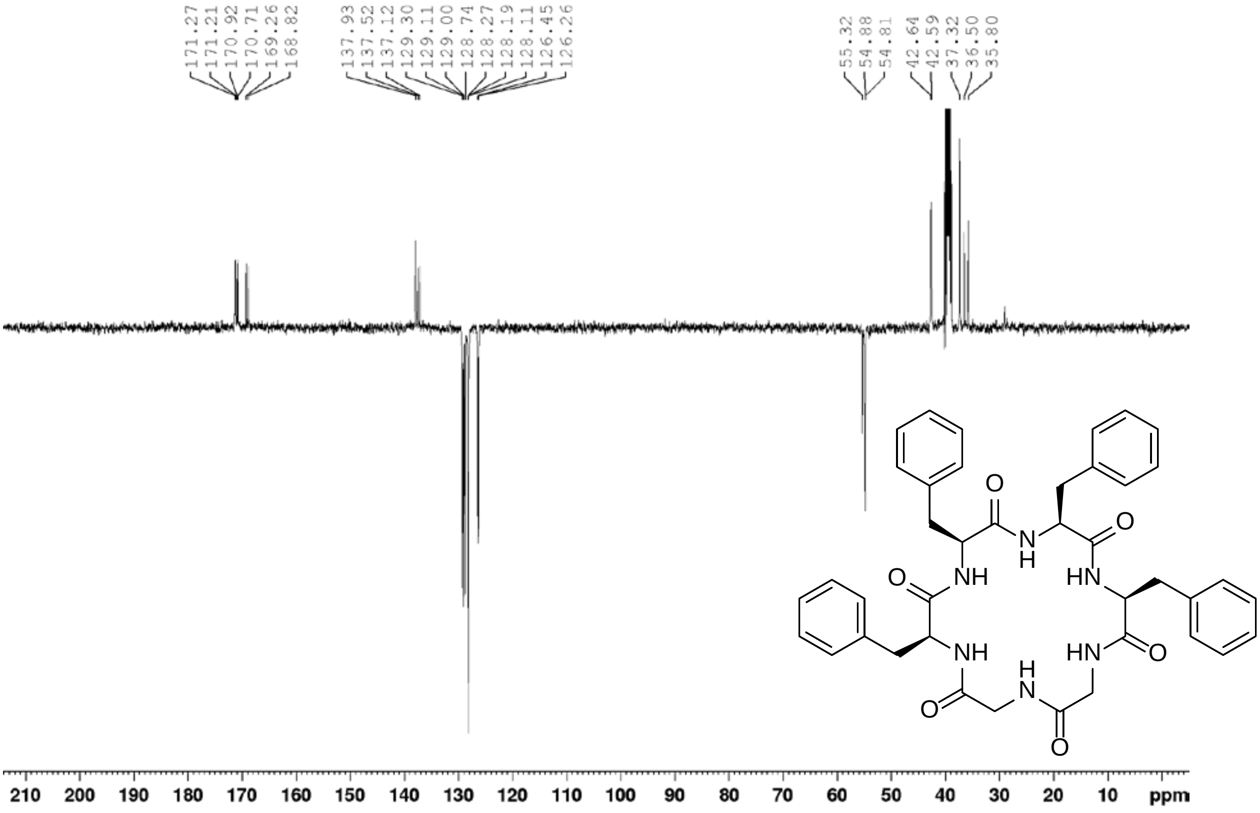

Figure S47. ^{13}C NMR spectrum of compound **C3** in DMSO-d_6.

6. HPLC chromatograms of samples from grinding experiments

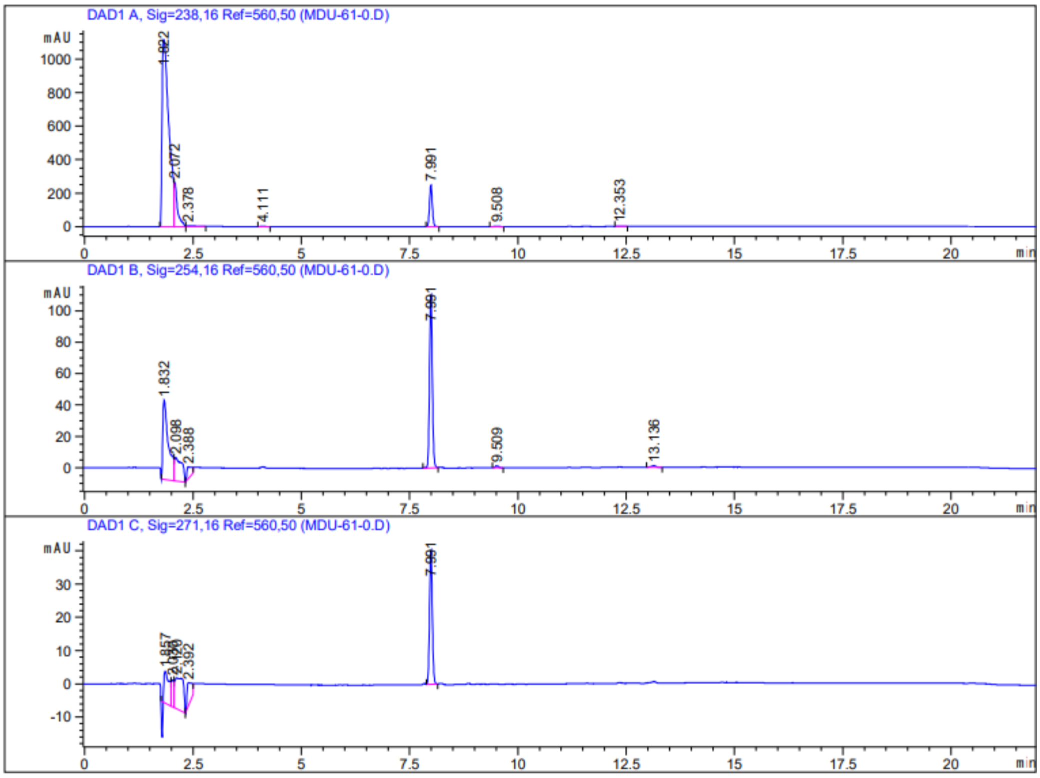

Figure S48. HPLC chromatogram of **C2** (retention time 7.991 min). Signal at 1.822 min corresponds to the solvent.

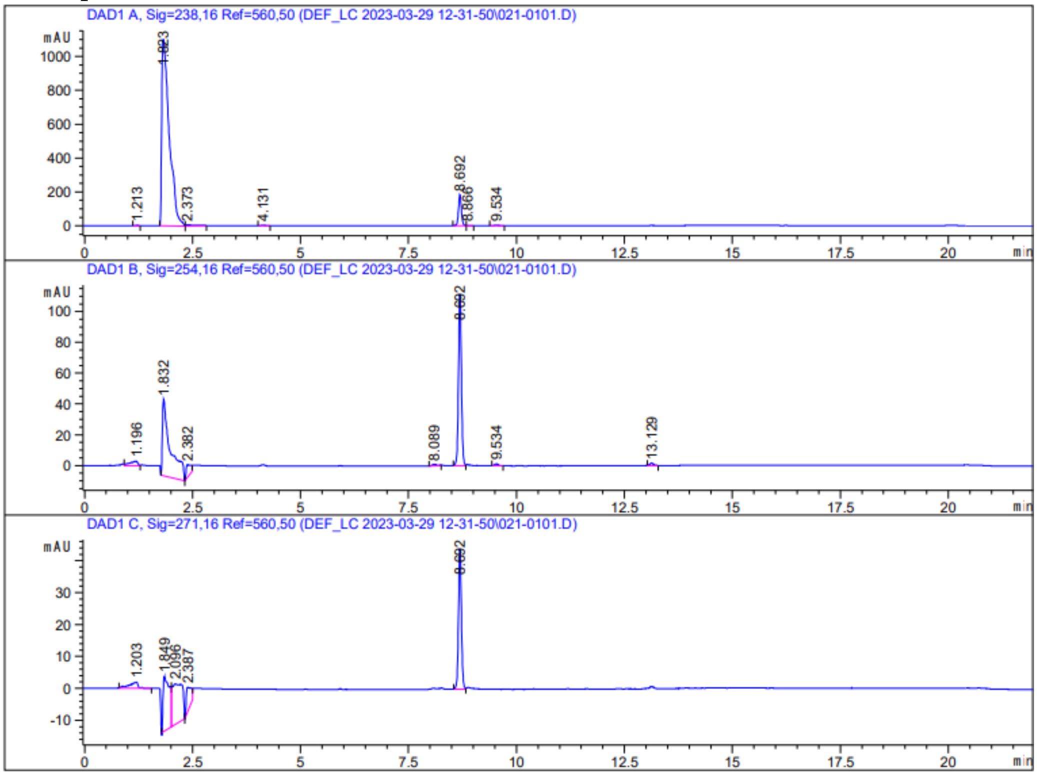

Figure S49. HPLC chromatogram of **C3** (retention time 8.692 min). Signal at 1.832 min corresponds to the solvent.

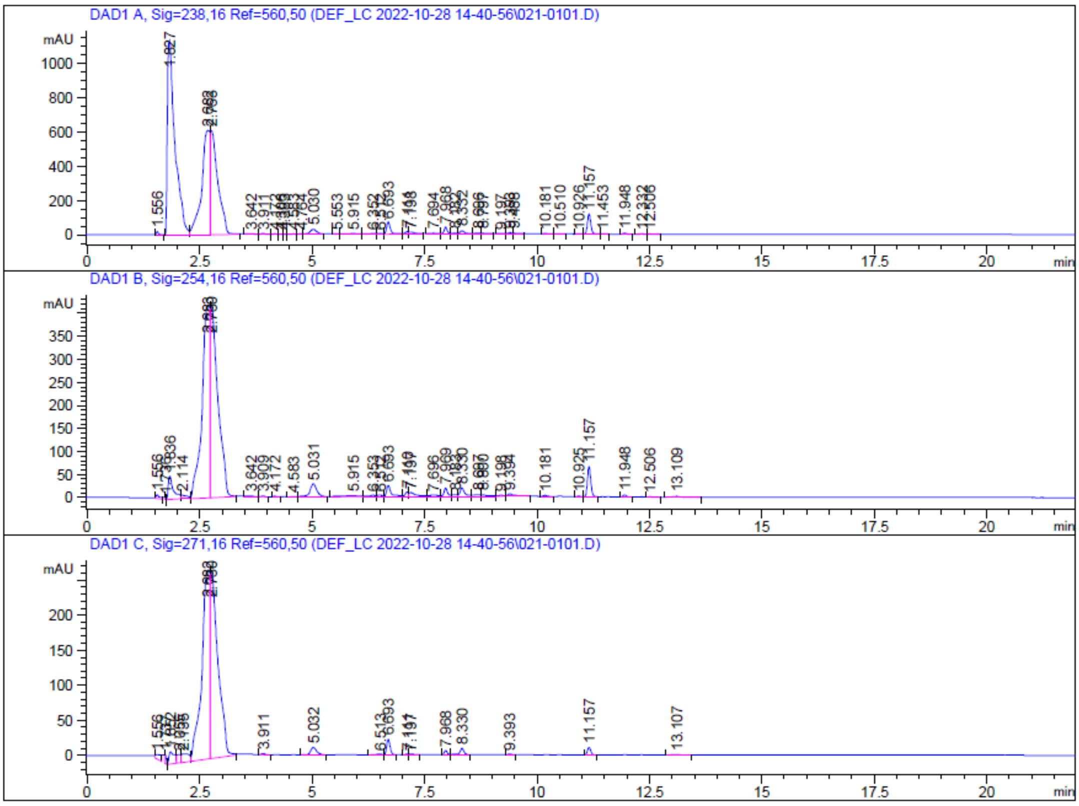

Figure S50. HPLC chromatogram of a sample of grinding experiment E1.

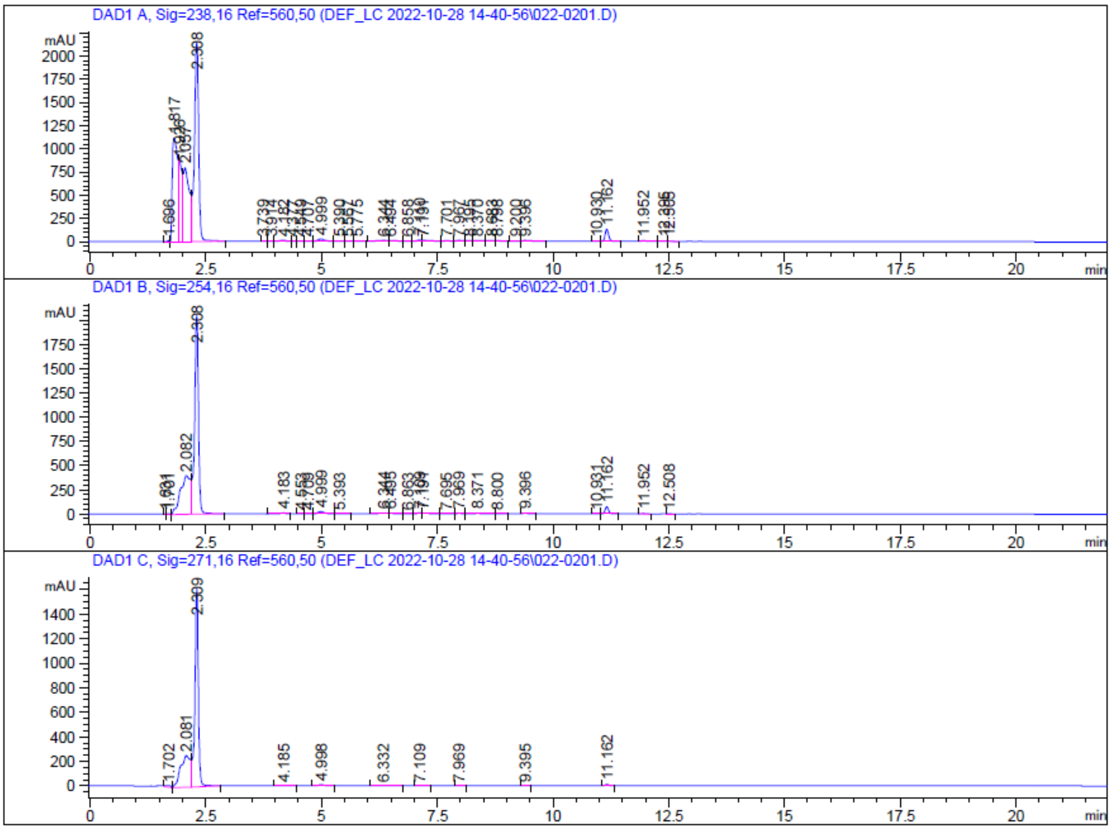

Figure S51. HPLC chromatogram of a sample of grinding experiment E2.

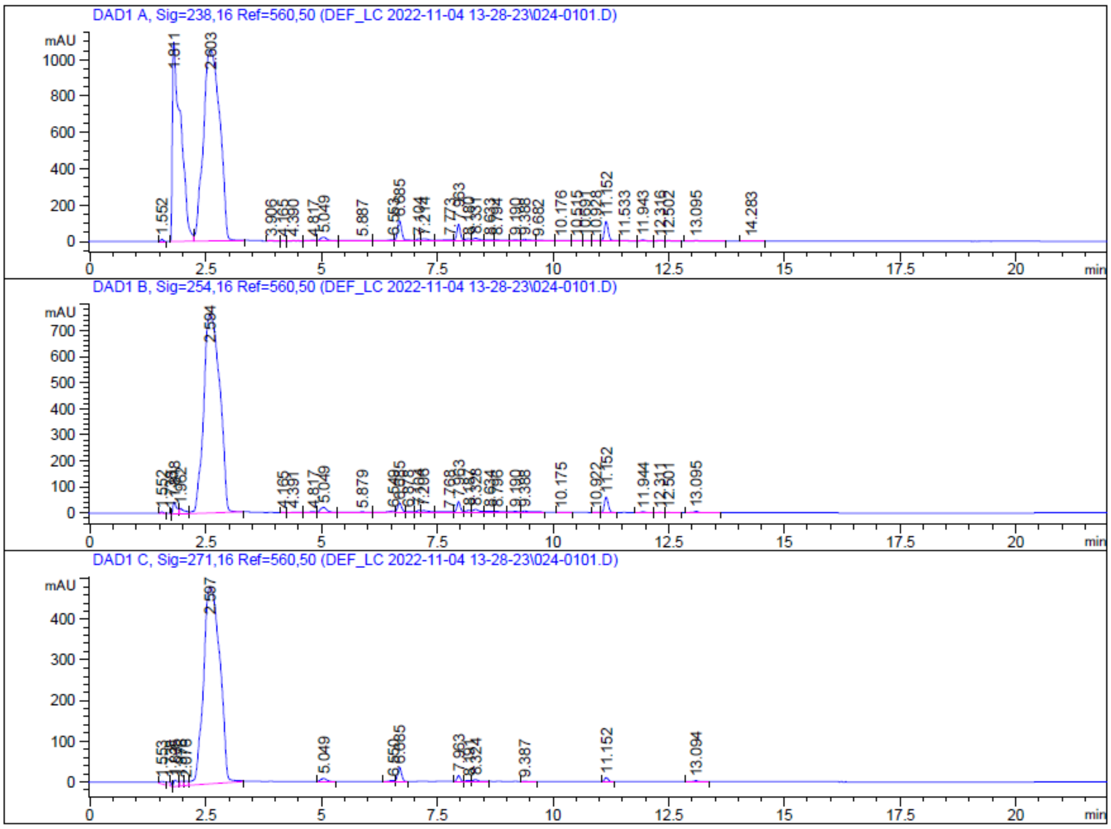

Figure S52. HPLC chromatogram of a sample of grinding experiment E3.

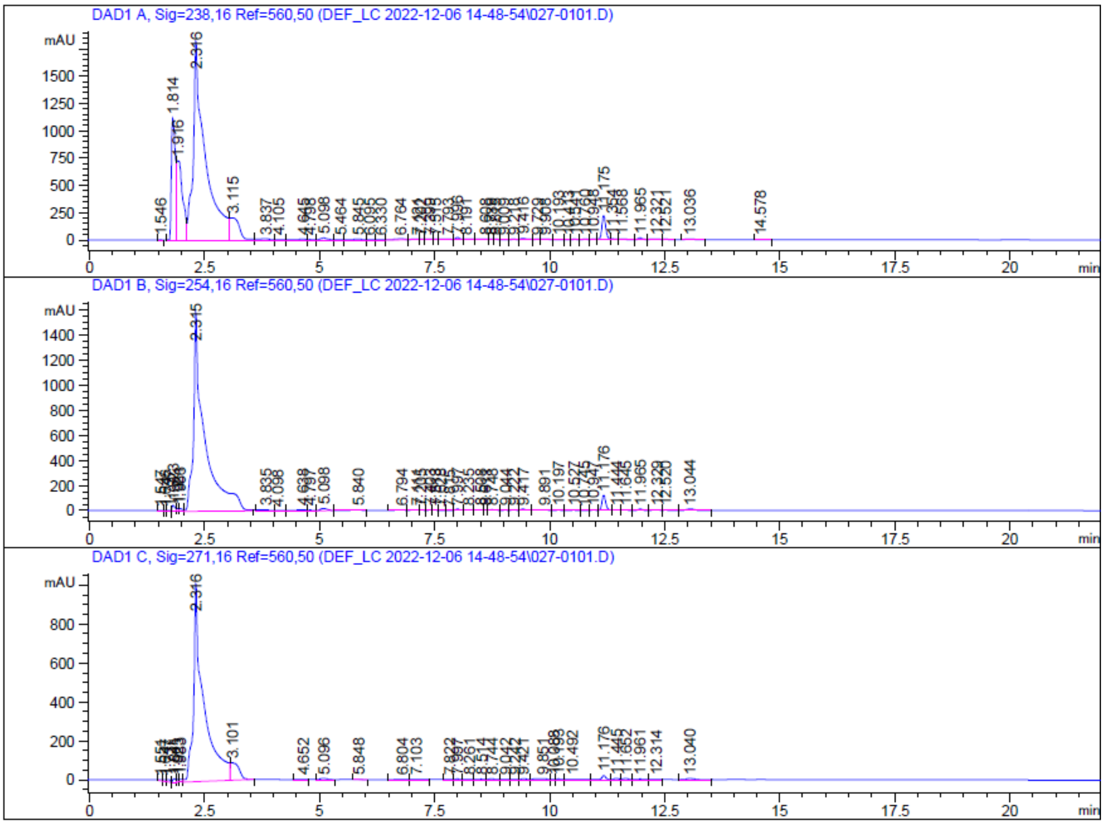

Figure S53. HPLC chromatogram of a sample of grinding experiment E4.

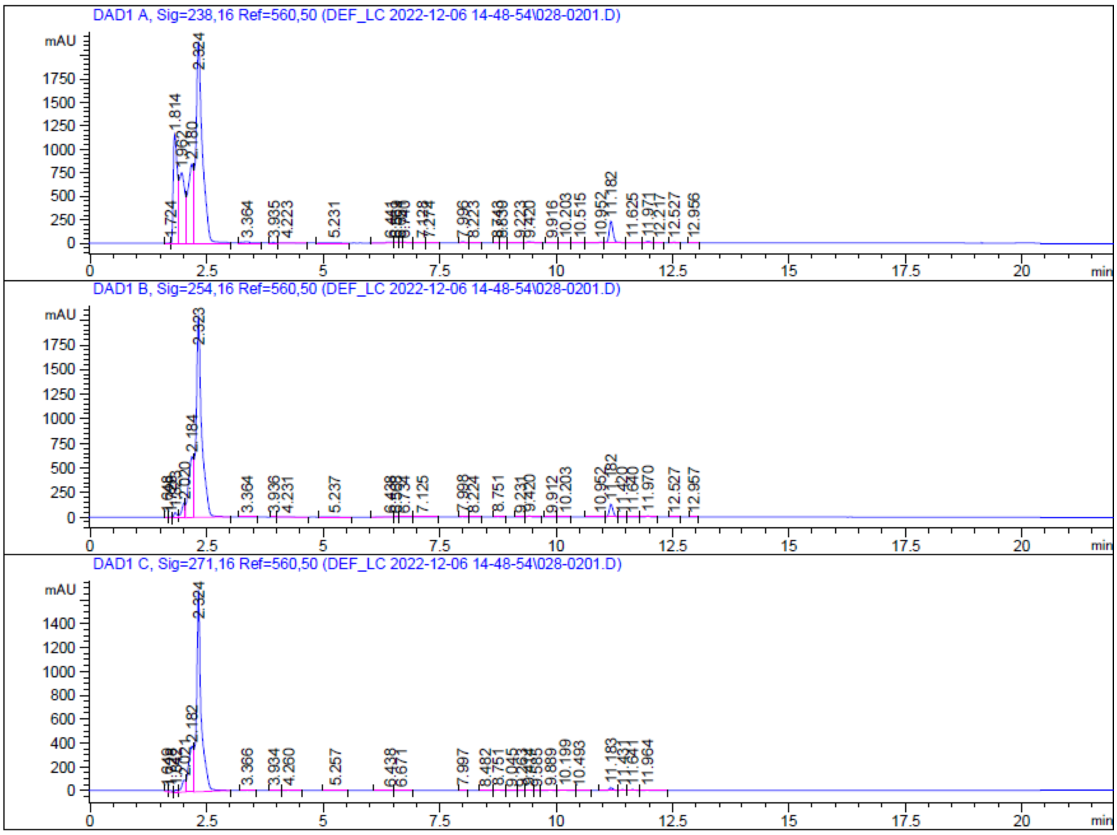

Figure S54. HPLC chromatogram of a sample of grinding experiment E5.

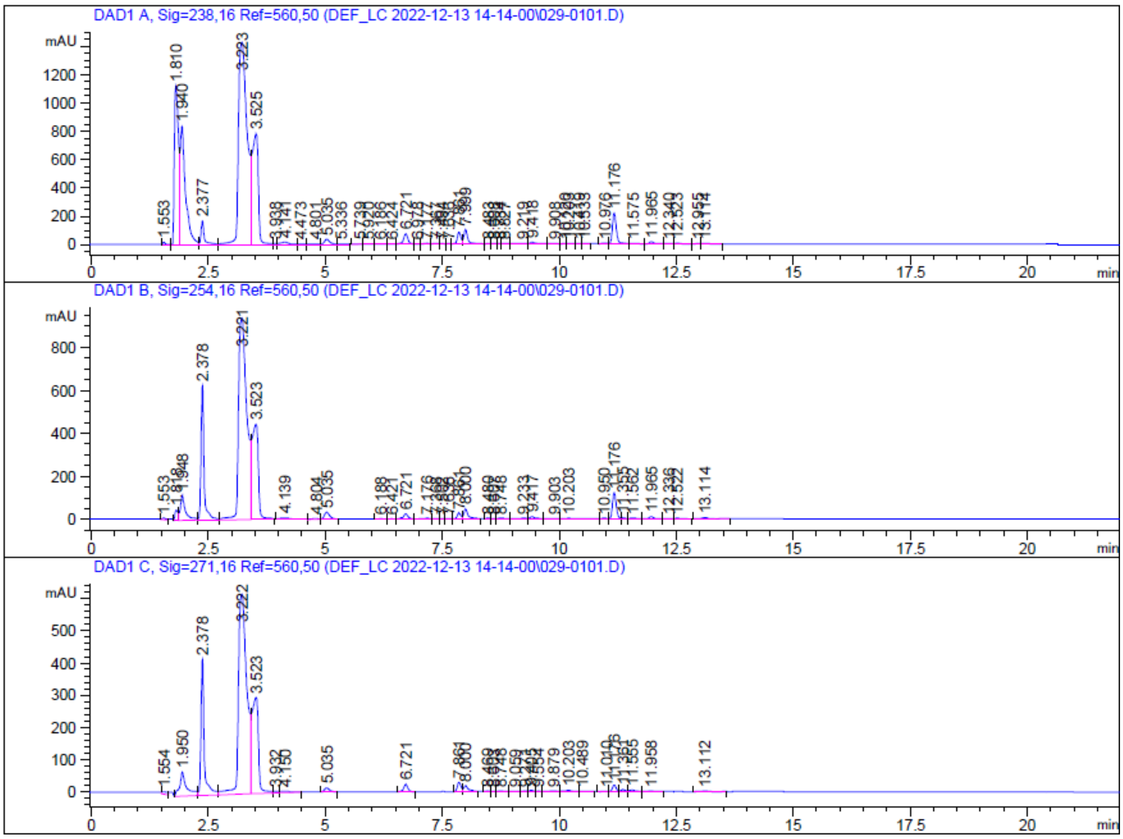

Figure S55. HPLC chromatogram of a sample of grinding experiment E6.

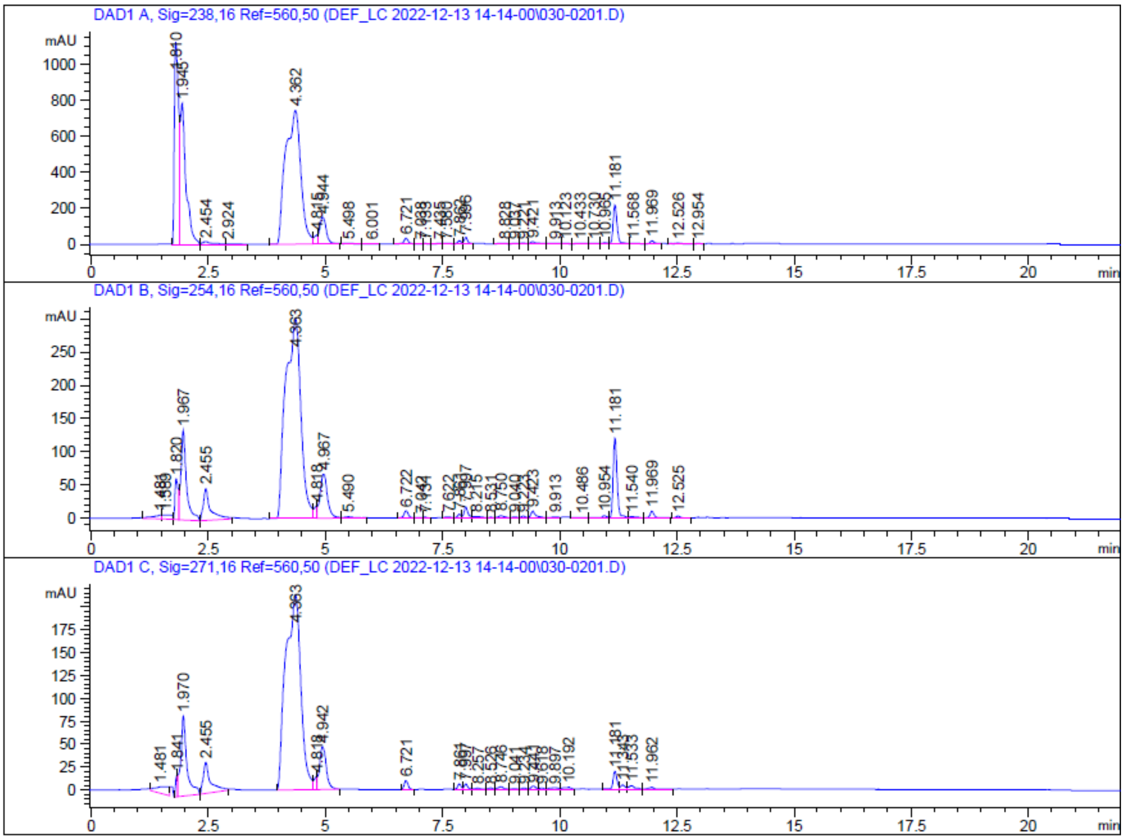

Figure S56. HPLC chromatogram of a sample of grinding experiment E7.

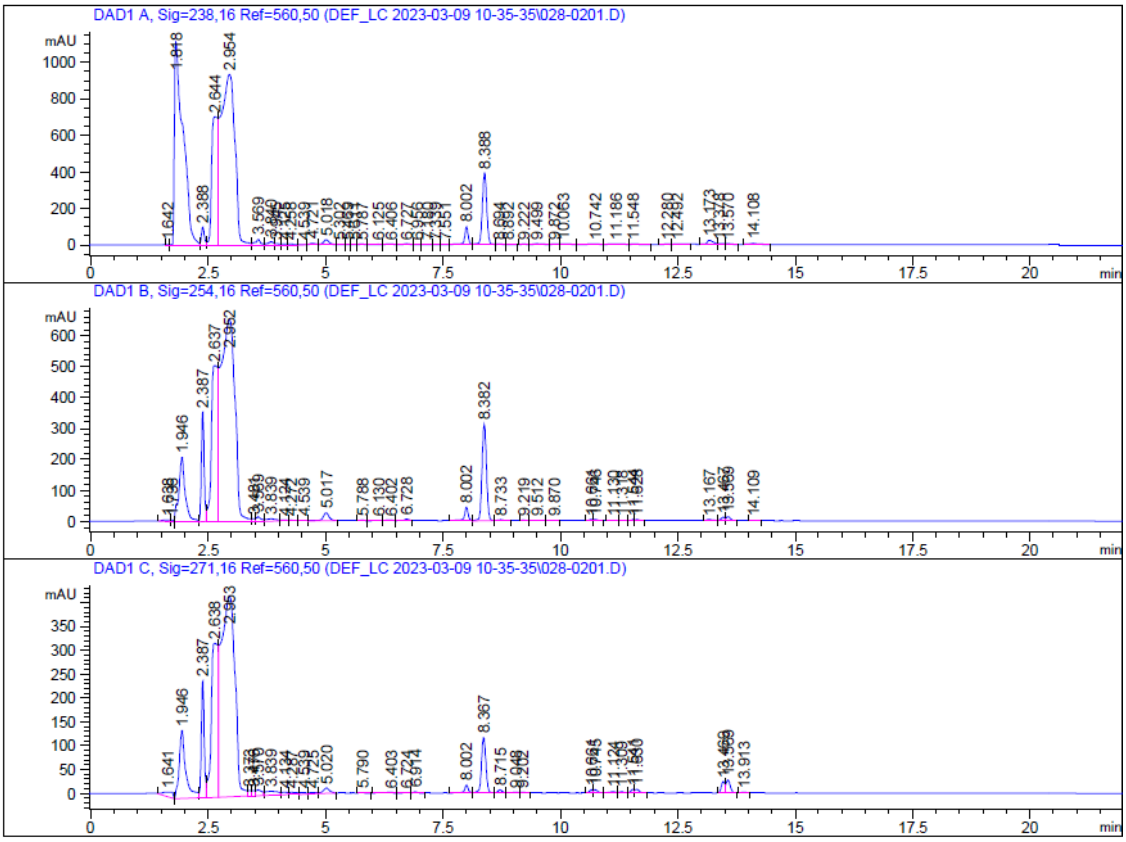

Figure S57. HPLC chromatogram of a sample of grinding experiment E8.

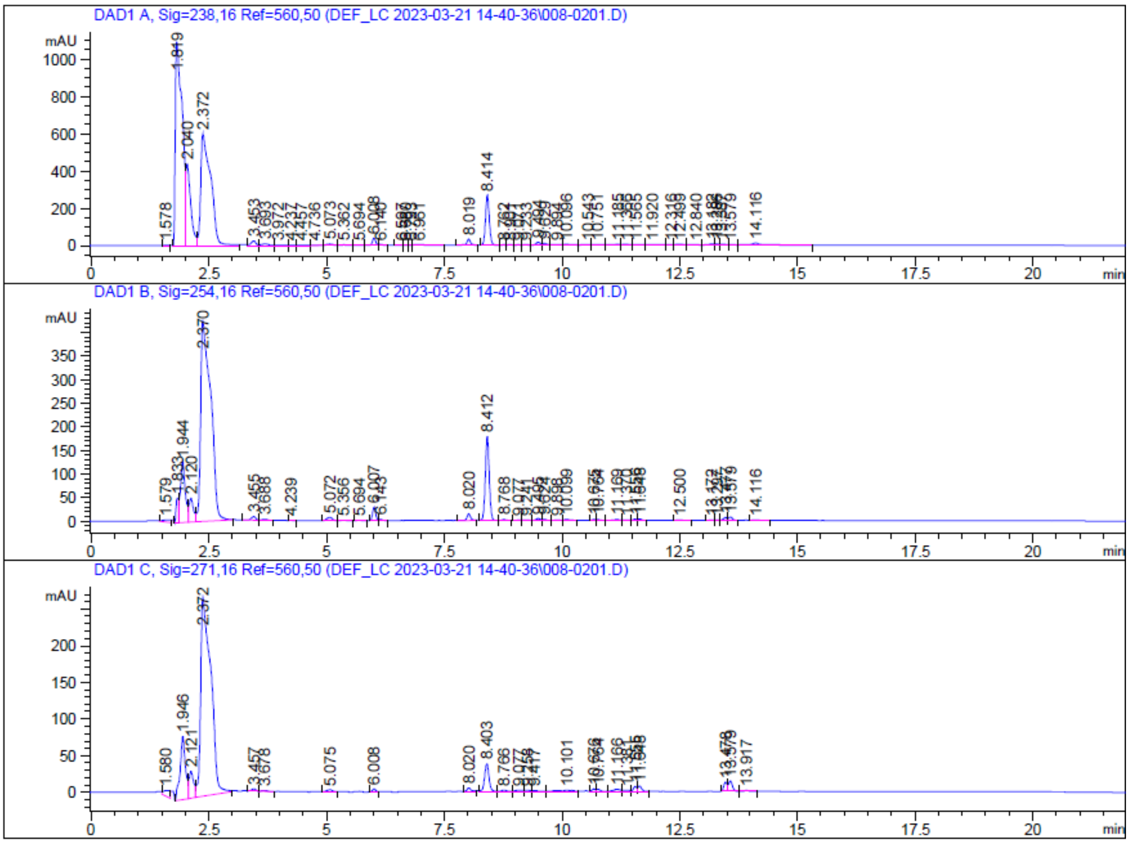

Figure S58. HPLC chromatogram of a sample of grinding experiment E9.

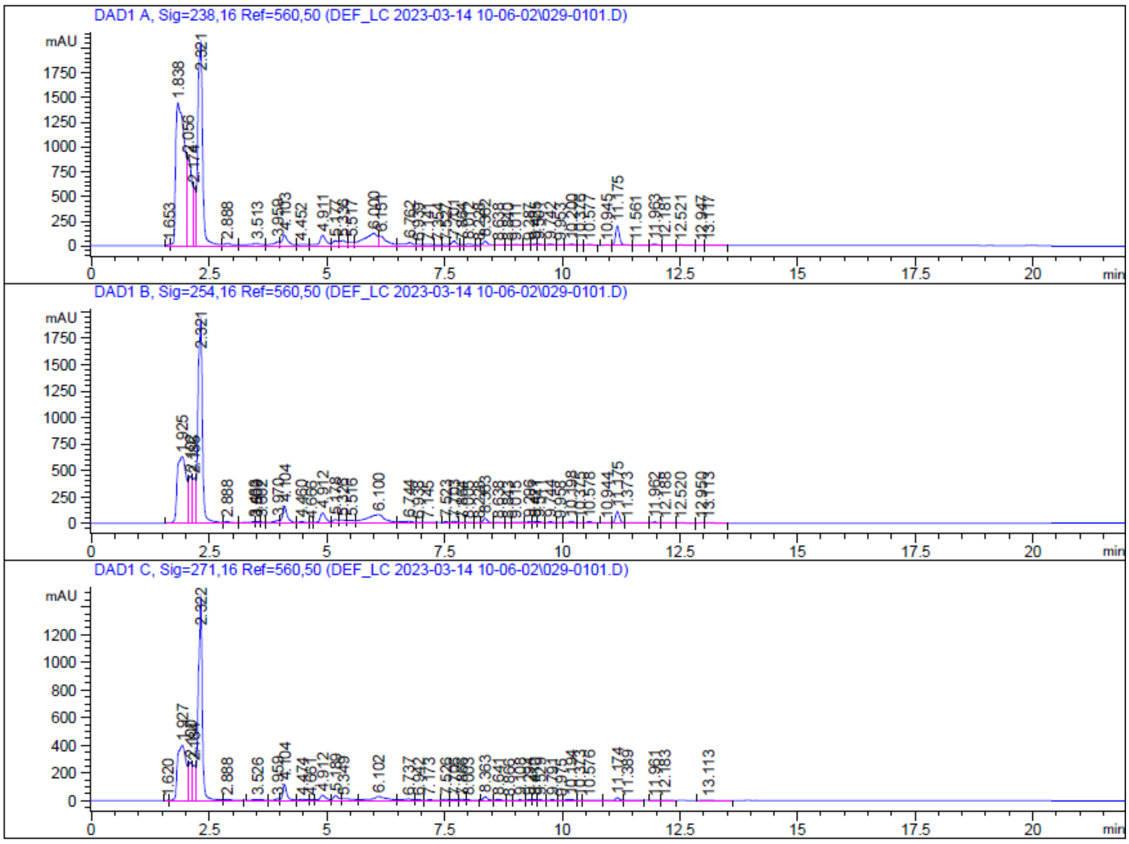

Figure S59. HPLC chromatogram of a sample of grinding experiment E10.

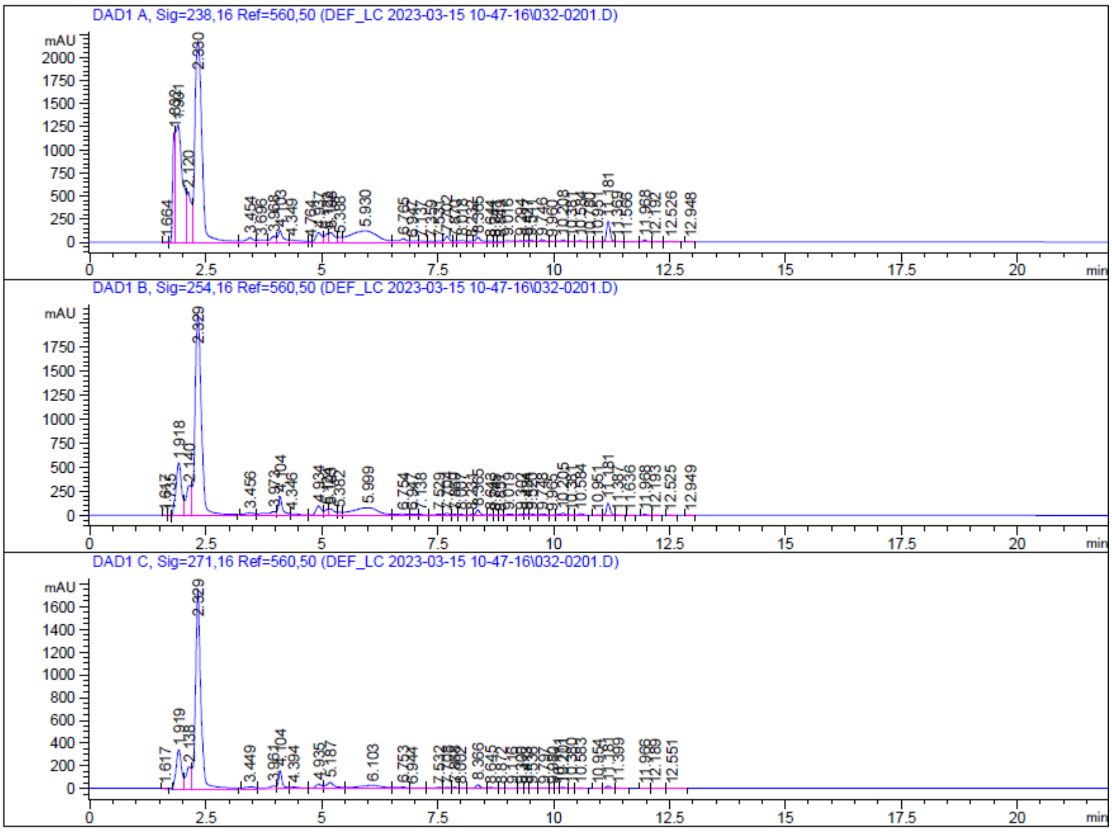

Figure S60. HPLC chromatogram of a sample of grinding experiment E11.

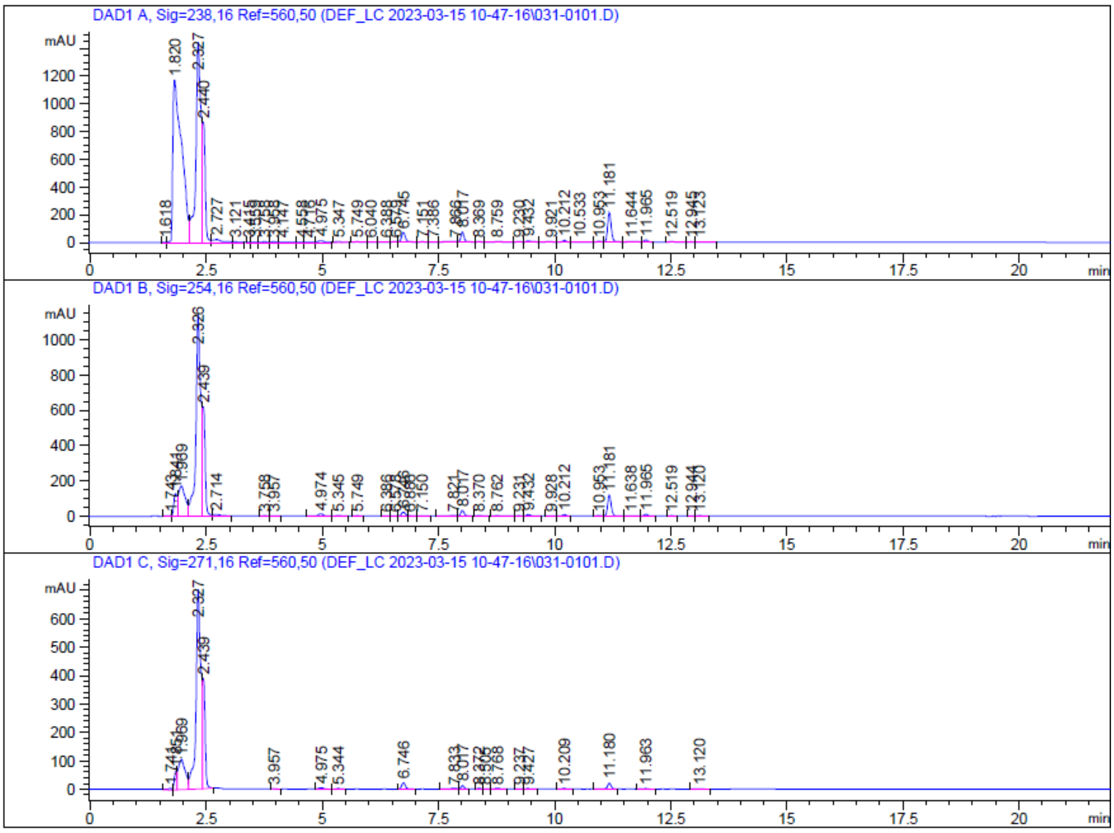

Figure S61. HPLC chromatogram of a sample of grinding experiment E12.

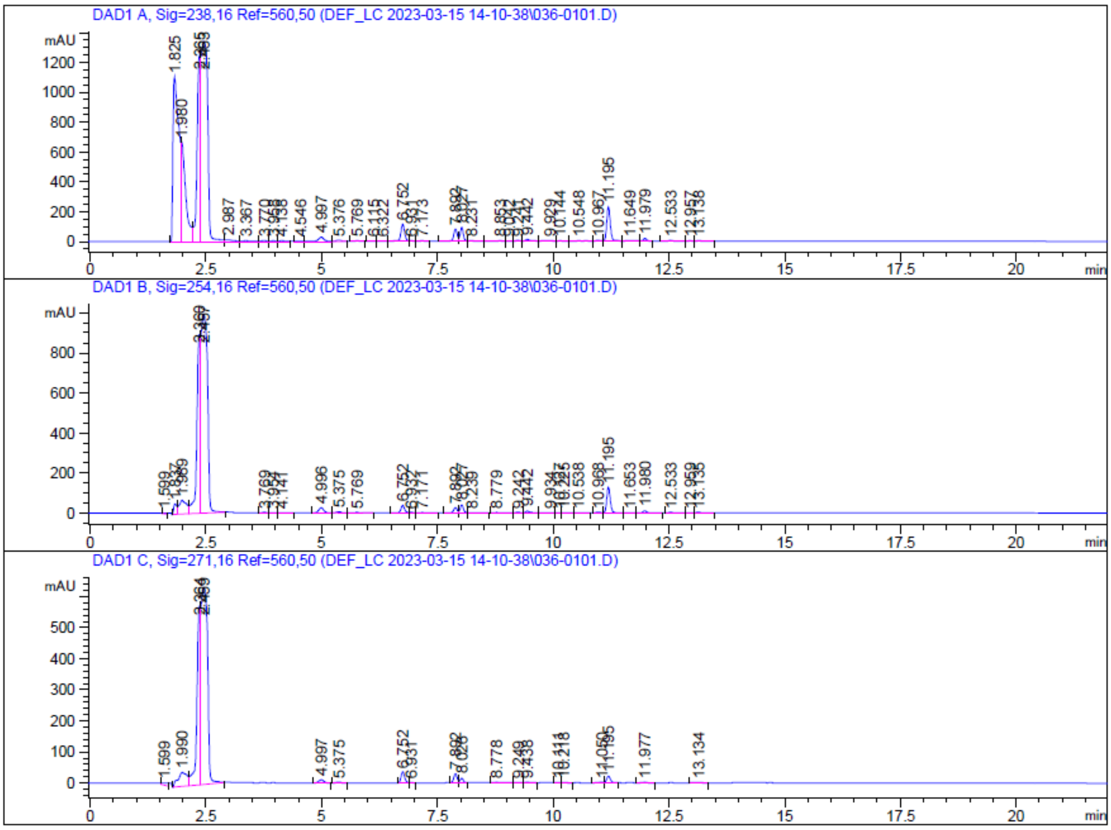

Figure S62. HPLC chromatogram of a sample of grinding experiment E13.

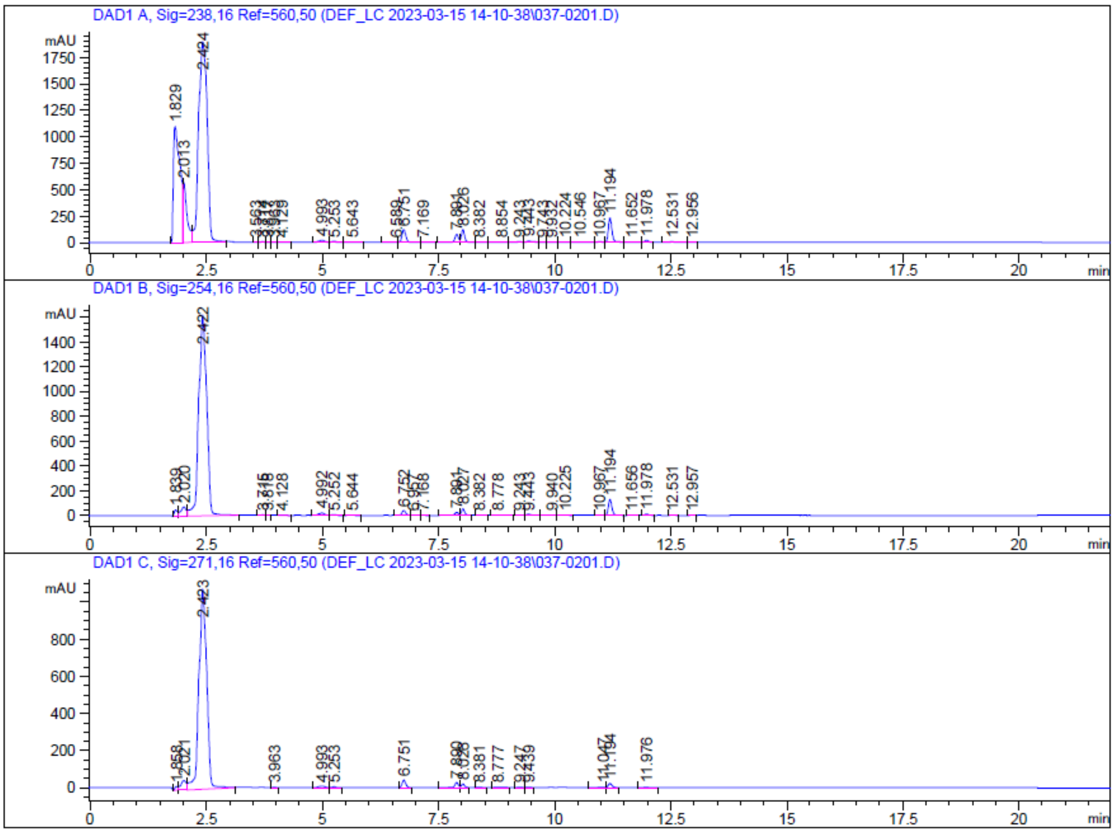

Figure S63. HPLC chromatogram of a sample of grinding experiment E14.

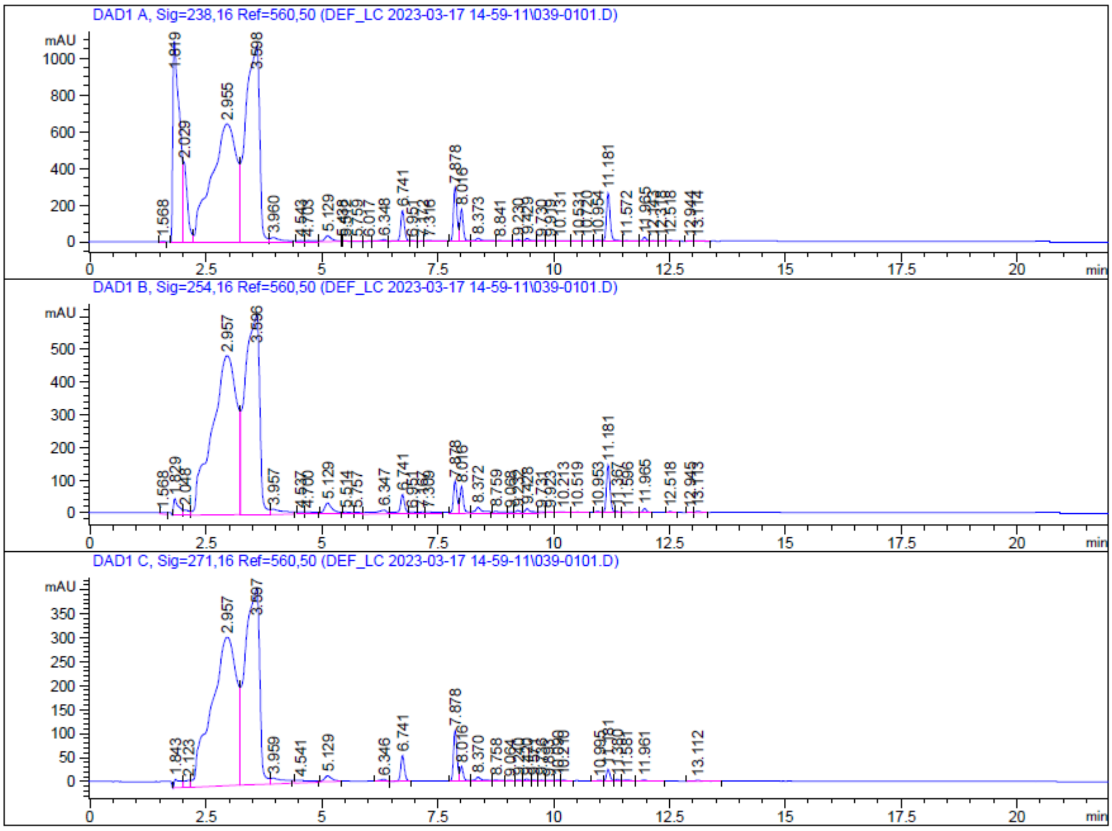

Figure S64. HPLC chromatogram of a sample of grinding experiment E15.

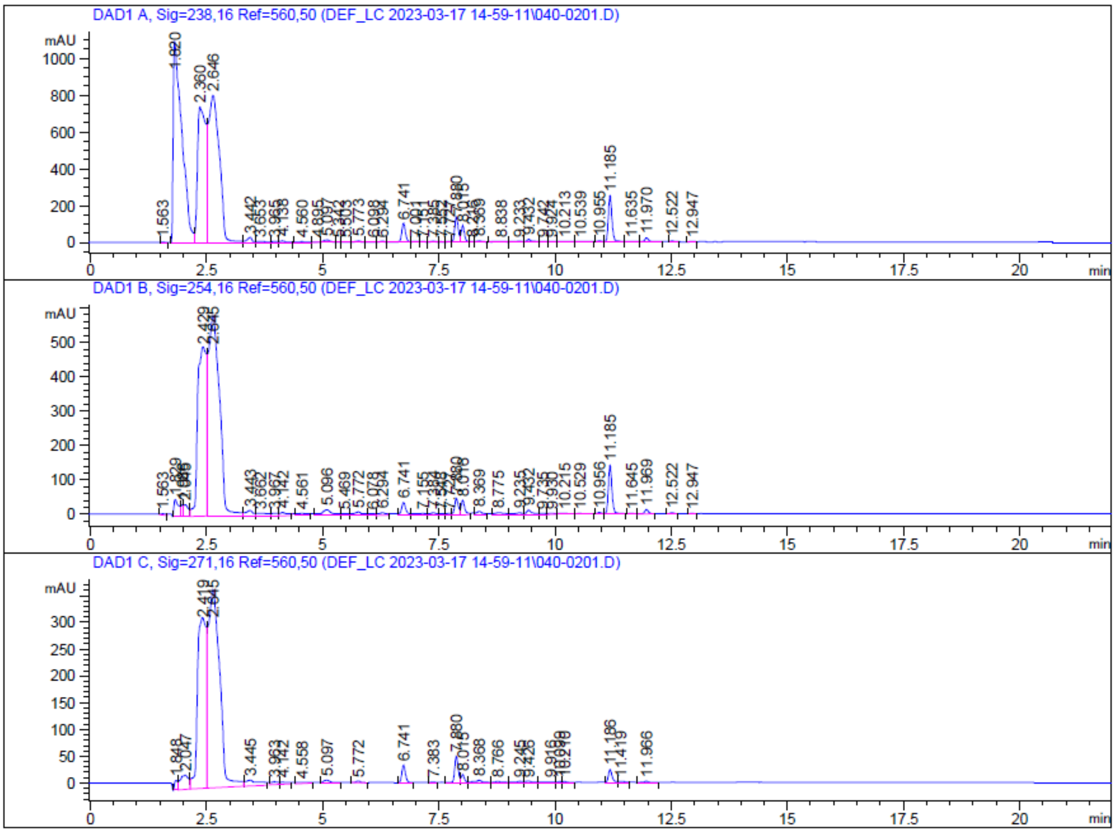

Figure S65. HPLC chromatogram of a sample of grinding experiment E16.

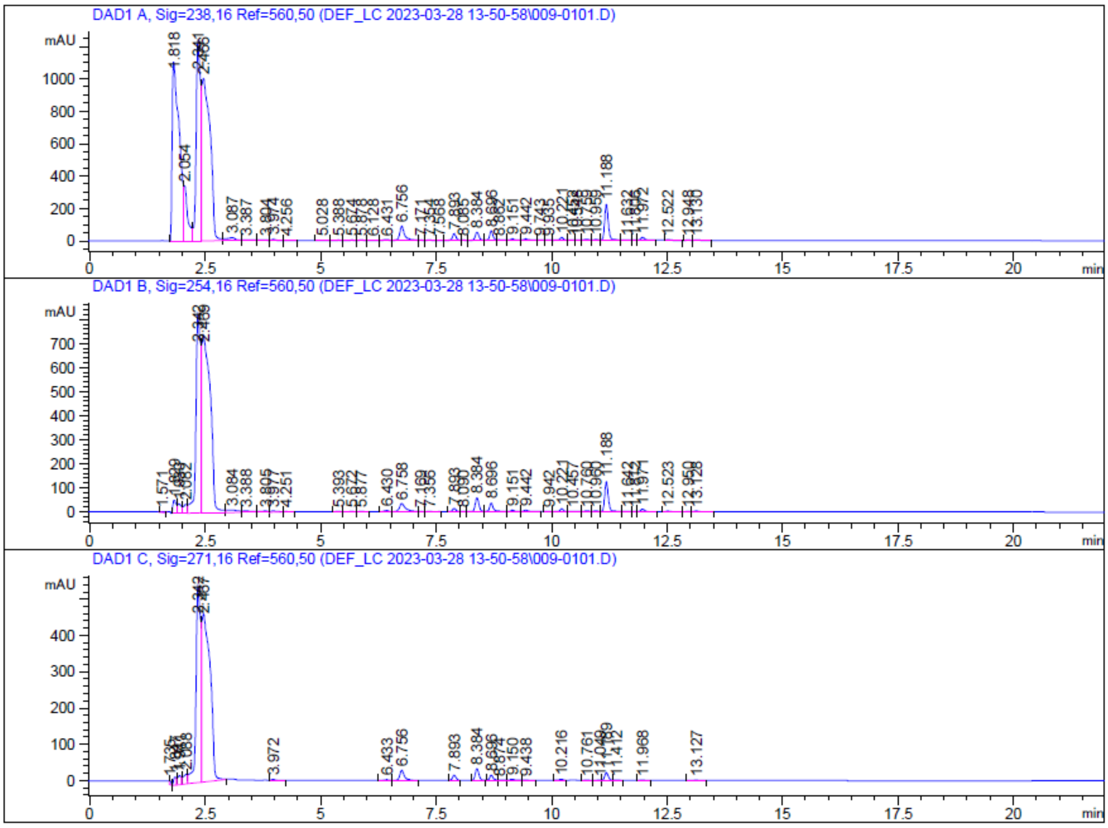

Figure S66. HPLC chromatogram of a sample of grinding experiment E17.

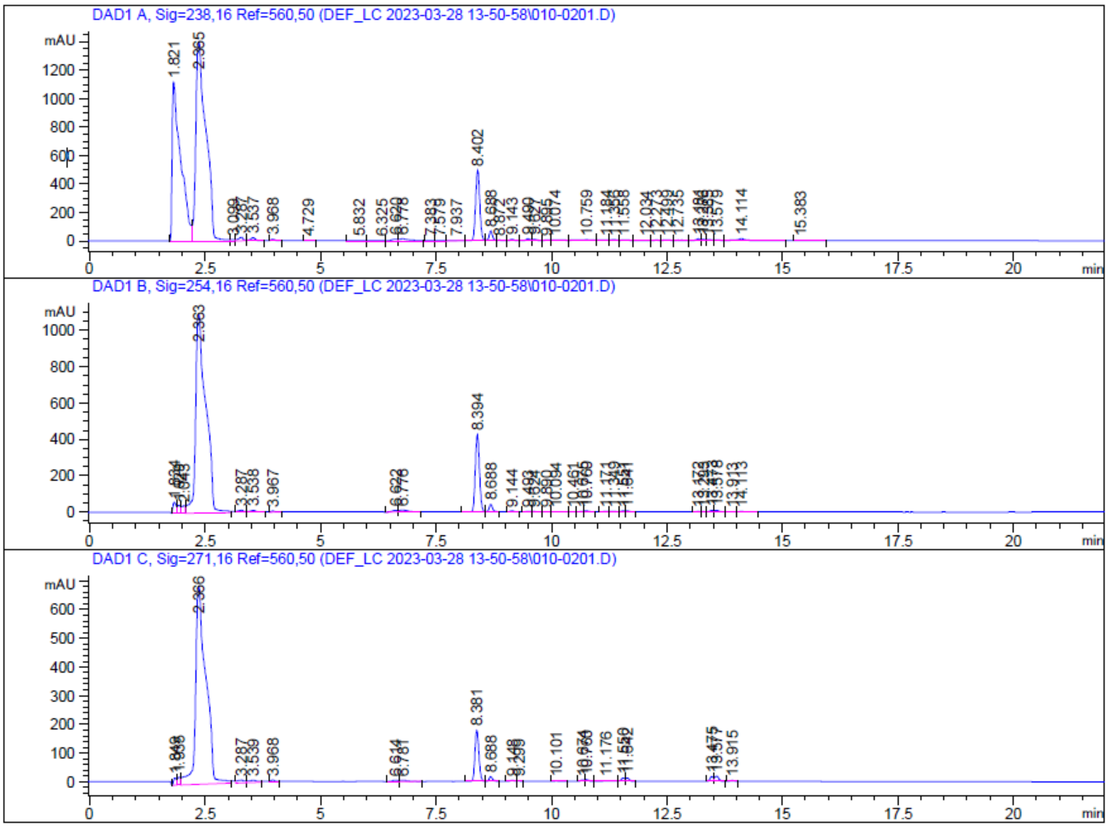

Figure S67. HPLC chromatogram of a sample of grinding experiment E18.

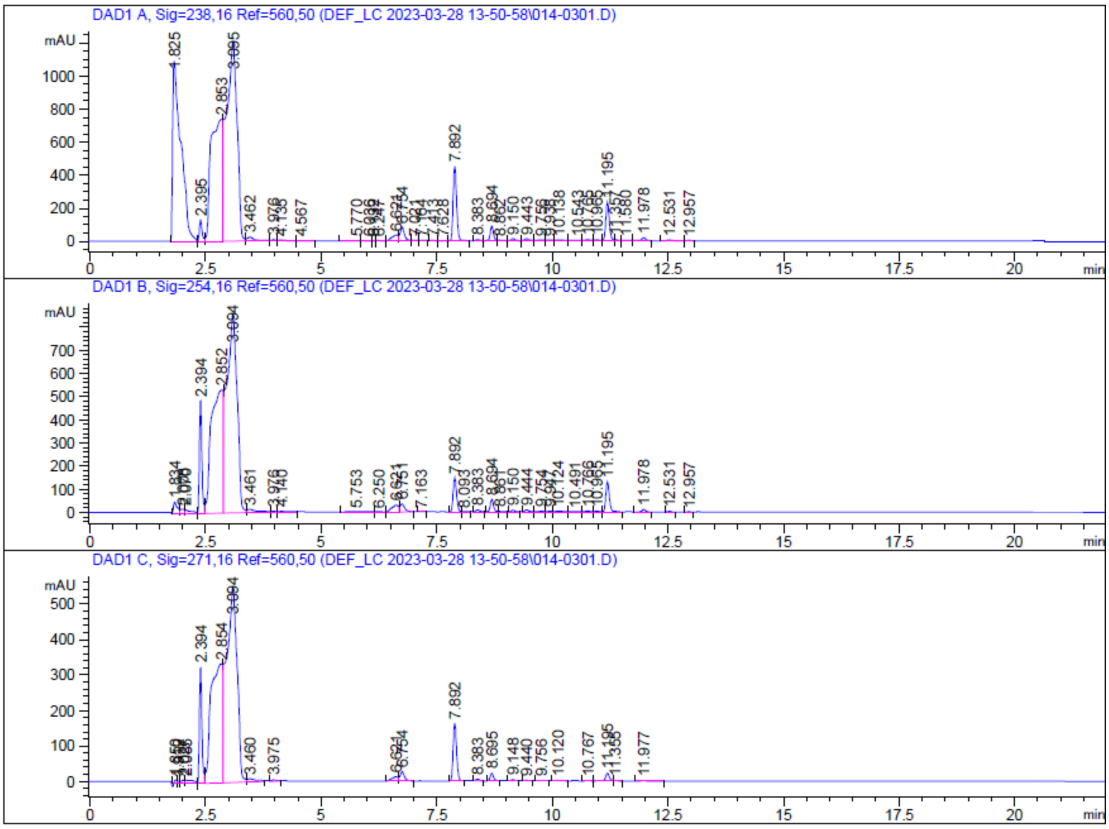

Figure S68. HPLC chromatogram of a sample of grinding experiment E19.

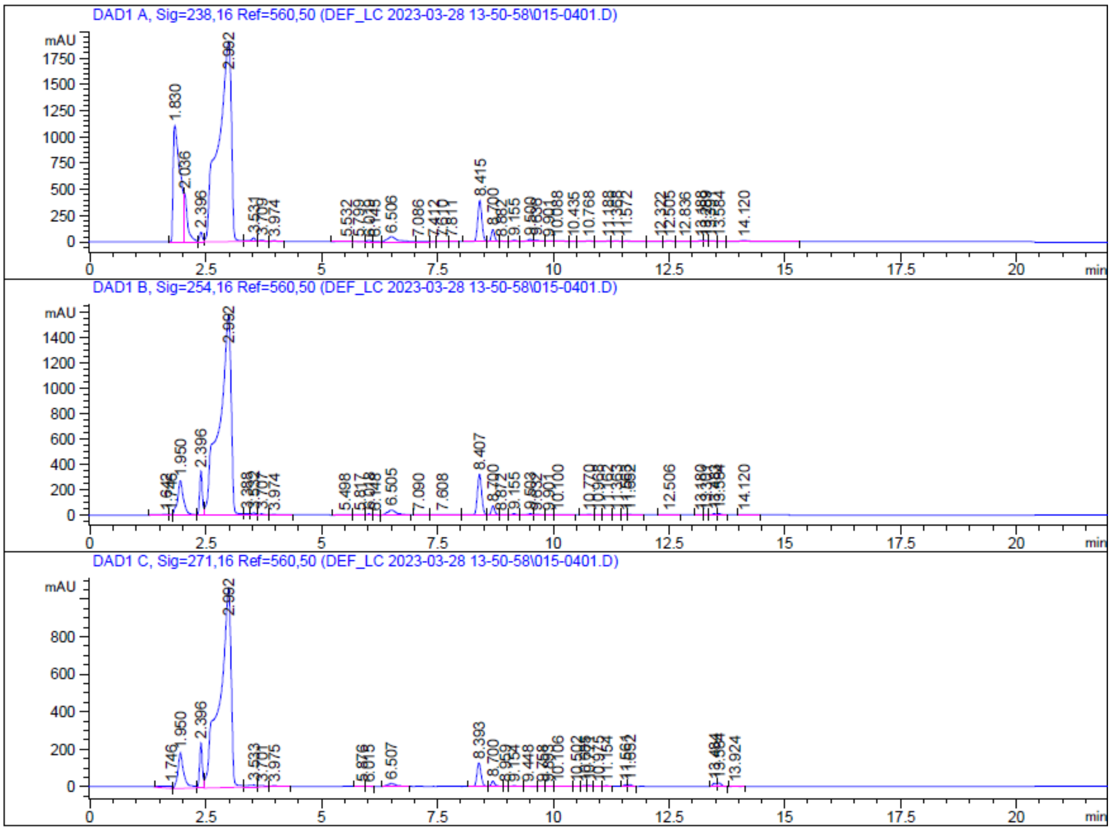

Figure S69. HPLC chromatogram of a sample of grinding experiment E20.

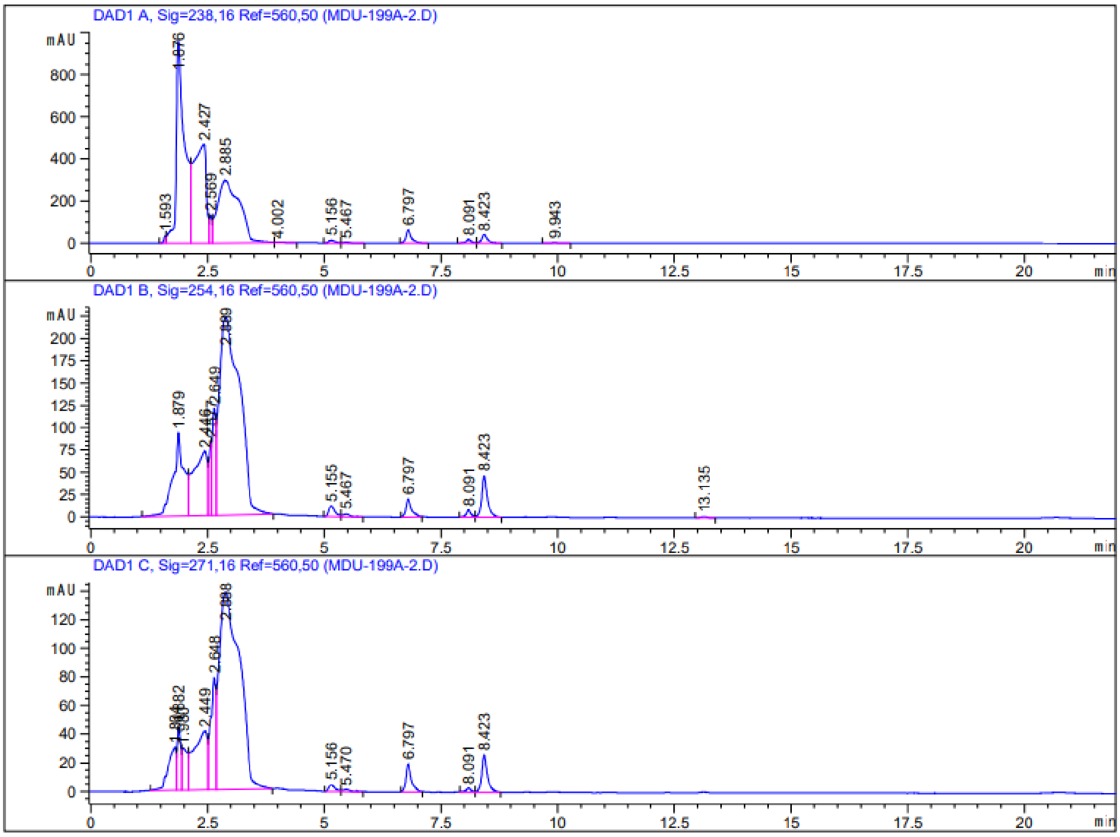

Figure S70. HPLC chromatogram of a sample of grinding experiment E21.

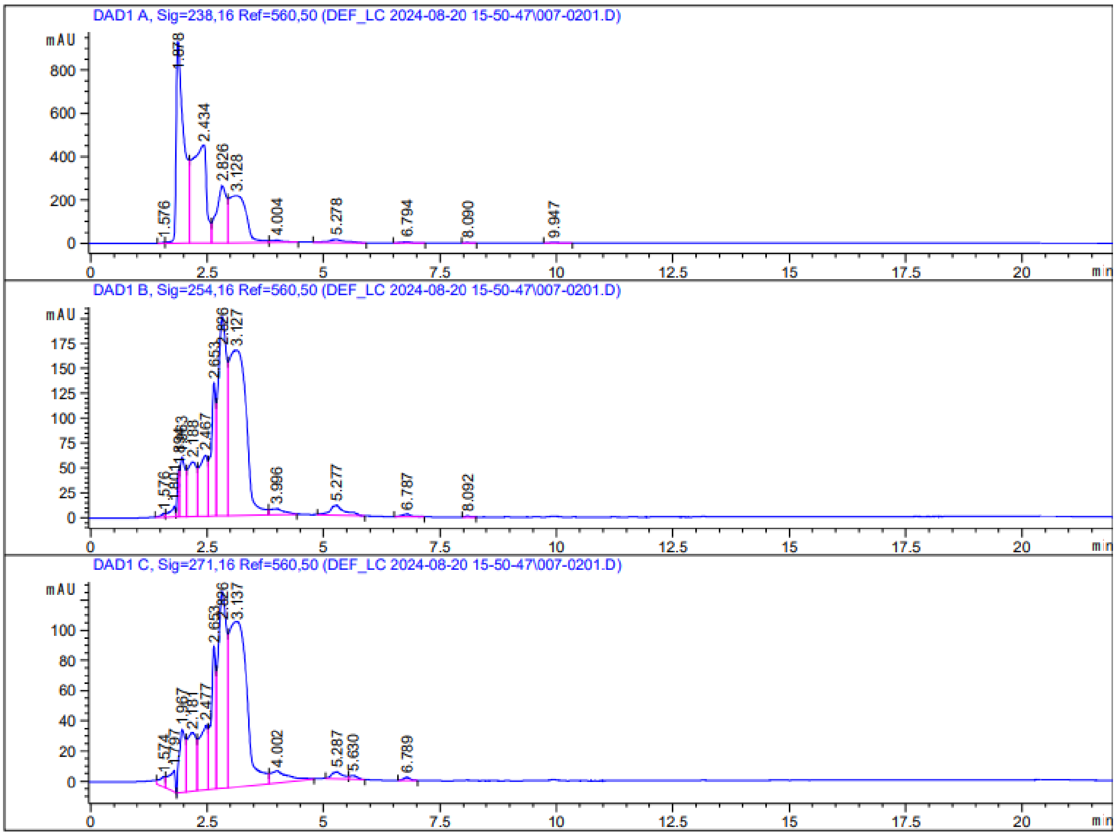

Figure S71. HPLC chromatogram of a sample of grinding experiment E22.

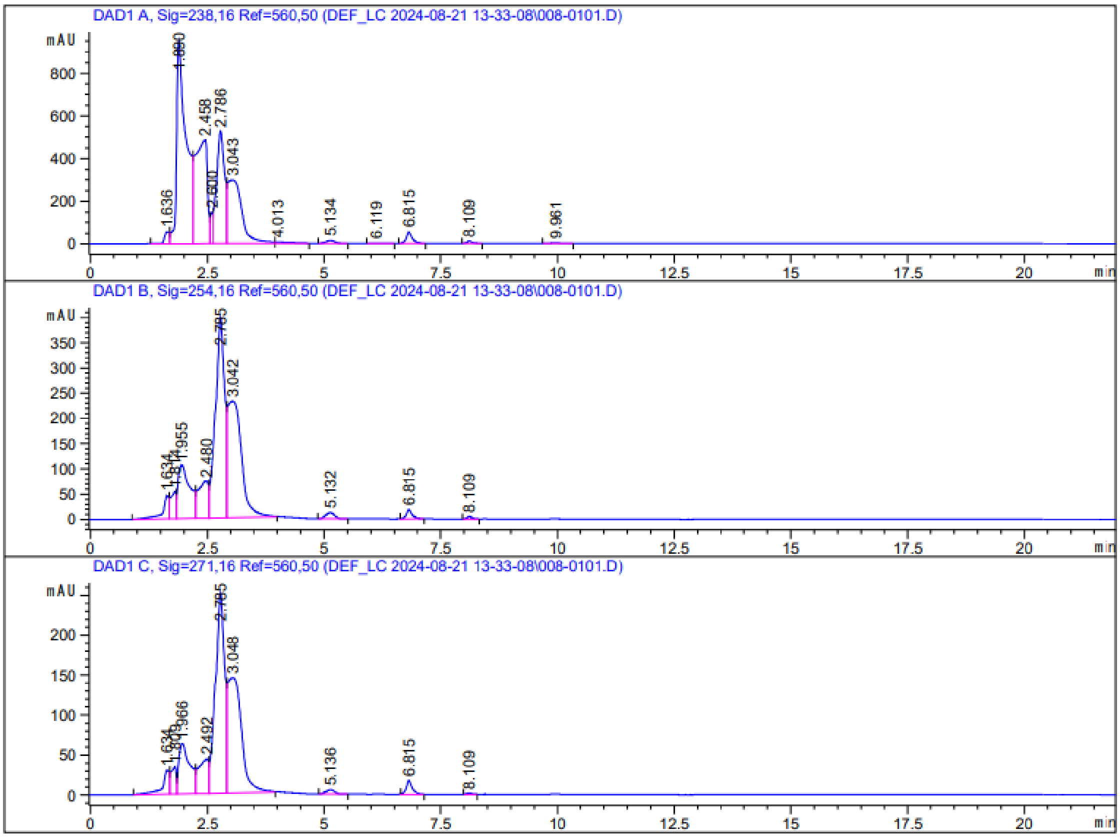

Figure S72. HPLC chromatogram of a sample of grinding experiment E23.

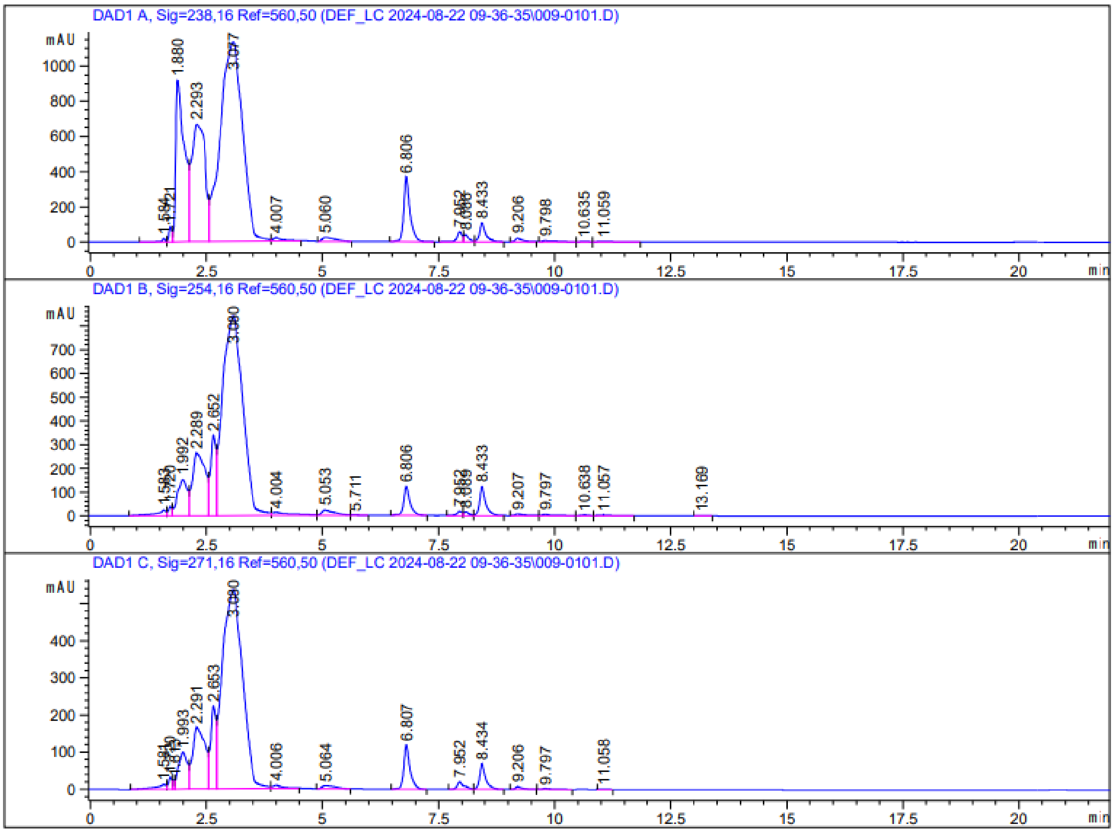

Figure S73. HPLC chromatogram of a sample of grinding experiment E24.

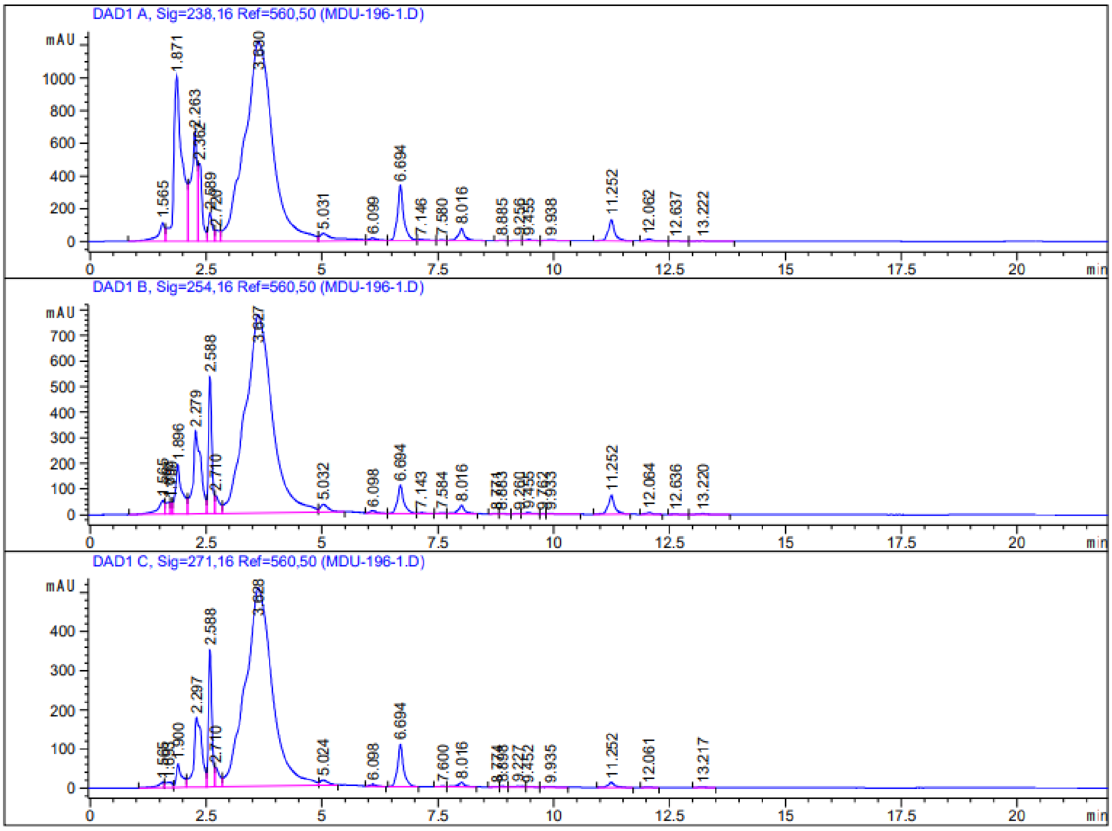

Figure S74. HPLC chromatogram of a sample of the scaled-up ball-milling experiment.
